# An early Pangaean vicariance model for synapsid evolution

**DOI:** 10.1038/s41598-020-70117-8

**Published:** 2020-08-04

**Authors:** Leonidas Brikiatis

**Affiliations:** Unaffiliated, 17564 Palaeo Faliro, Greece

**Keywords:** Palaeontology, Speciation, Carbon cycle, Evolution, Biogeochemistry, Climate sciences, Solid Earth sciences, Sedimentology

## Abstract

Genetic isolation due to geographic separation (vicariance) is the best understood cause of vertebrate speciation. Nevertheless, it has never been demonstrated in the fossil record across a wide range of taxa. Here, by reviewing in-depth the available data of the Late Palaeozoic (~ 350–250 million years ago), I reconstructed an early Pangaean junction-disjunction palaeogeographic model and showed that it coincides strongly with time-calibrated cladograms of the Late Palaeozoic synapsids (the primitive ancestors of modern mammals). The temporal development of the vicariant topology seems to fit closely with the emergence rhythm of the recovered synapsid taxa, suggesting vicariance due to Pangaean separation as the cause of early amniote evolution. The inferred vicariant topology also accounts for the observed pattern in the North American marine biostratigraphic units. Accordingly, the model demonstrates the link between the evolution of life on Earth and palaeogeographic evolution and strongly supports allopatric speciation through vicariance as the prominent mode of amniote evolution. Furthermore, correlations between state-of-the-art biochronostratigraphic charts and this palaeogeographic model suggest that the arido-eustasy model can explain the mid-Permian biotic extinction event and depositional cycles, such as the pre-Zechstein of the Central European Basin.

## Introduction

Vicariance is the geographical separation of previously sympatric populations due to the development of geographical and/or ecological barriers to gene flow^1^. Through vicariance, conspecific populations become genetically isolated and subsequently accumulate different mutations that render them reproductively incompatible resulting in the creation of new species^2^. One common way for temporally heterogeneous geographic barriers to form is via eustatic sea-level changes that produce seaways on low profile intercontinental land bridges^3^. Accordingly, transgressive stages, which occur when sea-levels are high, are expected to coincide with vicariance of the terrestrial biota, whereas regressive stages, which occur when sea-levels are low, are expected to coincide with geodispersals, and the opposite pattern is expected for marine biota^4^.

To verify a vicariance pattern, a junction-disjunction palaeogeographic model must correlate with phylogenetic topologies of multiple taxa^5^. Although vicariance is the best understood mode of speciation^1,2^, due to the absence of detailed scenarios of palaeogeographic changes and sufficiently resolved and representative phylogenetic trees, allopatric speciation has not been confirmed for a wide range of taxa in the vertebrate fossil record. Based on the most apparent episodes of sea-level change that affected the southern connections between the Uralian Seaway (URS) and the Palaeotethys, an early Pangaean junction-disjunction palaeogeographic model was reconstructed and compared to time-calibrated consensus cladograms (“clado-stratigraphic patterns”) of Late Palaeozoic vertebrates to determine whether a vicariance pattern can explain early synapsid evolution.

## Methods

This research started with the question of whether the Pangaea supercontinent constituted a single terrestrial surface or was characterised by fragmentation episodes that divided its biogeographic continuity. If the latter was true, then geographic isolation and allopatric speciation may have occurred in all of the lineages that were unable to cross the biogeographic barriers. The second goal of this research was to define the timing and duration of the exposures of the biogeographic barriers. To answer these questions, a Pangaean junction-disjunction model was designed in the form of an area cladogram. The topology of the cladogram was then compared with the phylogenetic topologies of the biota that were predicted to be affected by vicariance.

### Synthesis of the junction-disjunction model

**Figure 1:**
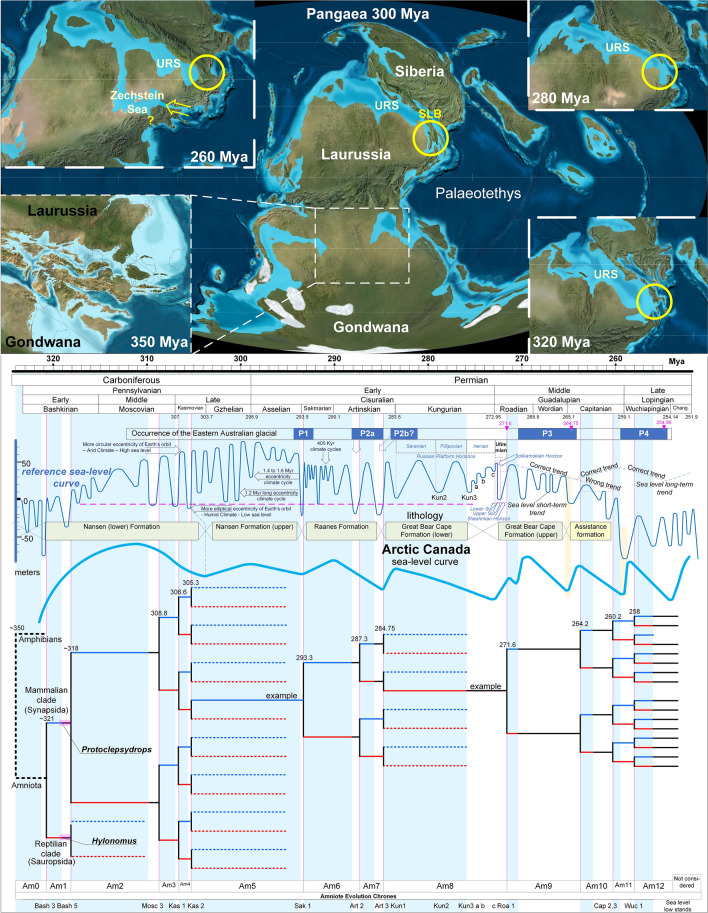
Top: Palaeogeographic reconstructions before (350 Mya) and after the accretion of Pangaea that emphasise the different exposure phases of the SLB (yellow circles) at 320, 300, 280, and 260 Mya. Bottom: Junction-disjunction topology of Pangaea under the influence of the reference sea-level fluctuation curve. The black branches correspond to the geodispersal intervals of the endemically developed lineages of eastern and western Pangaea (Siberia and Laurussia/Gondwana are represented by blue and red branches, respectively) during sea-level highstands. Node numbers correspond to divergence dates (in millions of years). The lithology of the Arctic Canada formations and the related sea-level curve are aligned for comparison. URS = Uralian Seaway, SLB = Siberian Land Bridge, yellow circles = temporal phases of the SLB. The palaeogeographic maps were modified from ref.^12^ with permission.

The Pangaea supercontinent was an assemblage of continental plates that formed a single landmass during the Late Palaeozoic^6^. The most important event in the Pangaean accretion process was contact between the Gondwana and Laurussia plates approximately 350 Mya. During this time, the huge Siberian plate, which included most of today’s Asian terrestrial territory, was located distal to Laurussia and was moving toward the Eastern European Platform^7,8^ (Fig. 1). During the merger of Gondwana and Laurussia in the Late Carboniferous ~ 323 Mya, Serpukhovian-Bashkirian boundary^9^, the Siberian plate was located proximal to the East European Platform of Laurussia, however, it did not fully merge with Laurussia to form Laurasia until the early Triassic (~ 250 Mya)^8^.

Until the merger of Siberia with Laurussia, these two continental plates were separated by the Uralian Seaway (URS), a shallow (100–150 m) seaway that was deepened only at its southern end^10^. The southern connection of the URS with the Palaeotethys was dominated by exposure to a narrow strip of land that connected the continents Laurussia and Siberia in the far eastern part of Pangaea and called the Siberian Land Bridge (SLB) (also called the Precaspian Isthmus^11^).

All of the current palaeogeographic reconstructions agree that the only unstable junction in the huge terrestrial continuity of the Late Palaeozoic multi-continental assembly of Pangaea was the SLB^6,7,11–14^, which consisted of mountains and low profile reliefs that were diachronically subject to sea-level transgression-regression cycles^15^. Therefore, it was important to develop a Pangaean junction-disjunction model that could focus exclusively on the exposures of the SLB to control the terrestrial connection between eastern (Siberian plate) and western (Laurussian and Gondwanan plates) Pangaea.

Recent detailed palaeogeographical reconstructions^12^ show that the Siberian and Laurasian plates have been positioned proximally since the Late Carboniferous (Pennsylvanian), thus it is reasonable to assume possible terrestrial connections between the Siberian and Laurasian plates during sea-level lowstands. Accordingly, a junction-disjunction sequence was reconstructed based on the strictest recorded sea-level lowstand events in the Palaeozoic by Haq and Schutter^16^, which were sampled from (low-subsidence) cratonic basins. For better accuracy, the sea-level curve was biochronostratigraphically recalibrated stage by stage upon the last update (August 2018) of the International Chronostratigraphic Chart (ICS)^17^. The recalibration was important because, relative to the chart in the most recent Geologic Timescale^18^, there were serious differences in the ages of the boundaries within the Permian Epoch, such as the Asselian/Sarkmatian, the Artinskian/Kungurian, the Kungurian/Roadian, and the Capitanian/Wuchiapingian. A characteristic stratigraphic scheme from the Arctic rift area accompanied by a sea-level curve^19^ was also aligned for comparison. Any deviation from the reference sea-level curve of Haq and Schutter^16^ was interpreted to be the result of poor biostratigraphic dating and/or tectonic activity in the Arctic rift area^19^.

The most up-to-date data for the occurrences of the Eastern Australian glacial period were also aligned to better understand the eustatic sea-level change under the terms of glacio-eustasy. The onset of the P3 glacial period is considered younger than the radioisotopic age of 271.6 Ma^20^, whereas the upper end of the P3 glacial period was set at ~ 264.2^21^. The precise age for the onset of the P4 glacial period was set at 259.5 Mya based on the coinciding sharp drop in seawater temperature in the biogenic apatite geochemical record of low-latitudinal sections^22^, whereas the end of the P4 glacial period was set at 254.5 Mya based on radioisotopic dating^20^. The dating of the P2 glacial period is after ref.^22^ and the P1 glacial after ref.^20^.

#### Late Carboniferous–Early Permian palaeogeographic evolution

The proposed junction-disjunction model together with the currently available palaeoenvironmental data suggest a detailed palaeogeographic scenario for the early development of Pangaea, which forms the basis for the vicariant model shown in Fig. 1. Around the Mississippian/Pennsylvanian boundary, the extreme Bash 3 sea-level lowstand resulted in the first ephemeral connection between the Siberian plate and Laurussia ~ 321 Mya. A second ephemeral connection occurred during the remarkable Bash 5 lowstand ~ 318 Mya. Since then, periodic exposures of the SLB are consistent with the lowstands that outstrip a specific sea-level in the reference sea-level curve (magenta dashed line in Fig. 1). Since the Kungurian, the palaeoenvironmental data from the URS deviate from the reference sea-level curve because of orogeny in the SLB area^7^. Thus, after the latest, major Artinskian transgression^23^, which is likely related to glacial melting in the Southern Hemisphere^24^, the marine environment of the URS became more restricted during the mid-Early Kungurian as evidenced by conodont palaeodistributions^25^ and salt deposition in the Peri-Caspian basin^7^. This trend led to the late Kungurian exposure of the SLB and the occurrence of predominantly terrestrial environments in the southern URS area, which were dominated by non-marine ostracods^26^. Davydov^11^ independently demonstrated the exposure of the SLB (Precaspian Isthmus) at the Asselian/Sakmarian boundary through marine faunal distribution data, but he attributed the subaerial exposure to the orogenic initiation. The reference sea-level curve shows, however, that the pre-Kungurian exposures of the SLB were due to eustatic rather than tectonic causes (Figs. 1 and 4).

By the early Roadian (early Kazanian; ~ 273 Mya), a marine transgression invaded from both the north^27^ and the south^26^, which broke up the SLB. However, by the late Roadian (late Kazanian), a marine regression (very likely related to the formation of the P3 glacial cycle) re-established the terrestrial environments in the southern URS^26^. That terrestrial regime persisted until the end of the Wordian^28^.

#### Middle-to-Late Permian palaeogeographic evolution

In the predominantly terrestrial facies of the SLB during the Middle Permian, the allopatric speciation mode of the model changes from “active” to “passive”^3^. Thus, in contrast to the long-lasting vicariance intervals and short periods of geodispersals (during sea-level lowstands) in the Early Permian, during the Middle and Late Permian allopatric speciation was only possible through in situ vicariance during sea-level highstands.

Detailed ostracod studies in the Peri-Caspian area have highlighted the possibility of a Palaeotethyan connection through an SLB break only during the lower part of the Tatarian (Severodvinian). The marine-like ostracod assemblages in the south of the Cis-Ural marginal deflection match the marine ostracods of the Olenekian (Middle Triassic) and contain numerous peculiar endemic cytherid species^29,30^ and smaller types that are morphologically similar to the genus *Paracypris*^15^. Later ostracod studies of the Permian/Triassic boundary confirmed the presence of *Paracypris* in the Late Permian-Early Triassic peri-Palaeotethyan margin (ref.^31^ and references therein).

**Figure 2:**
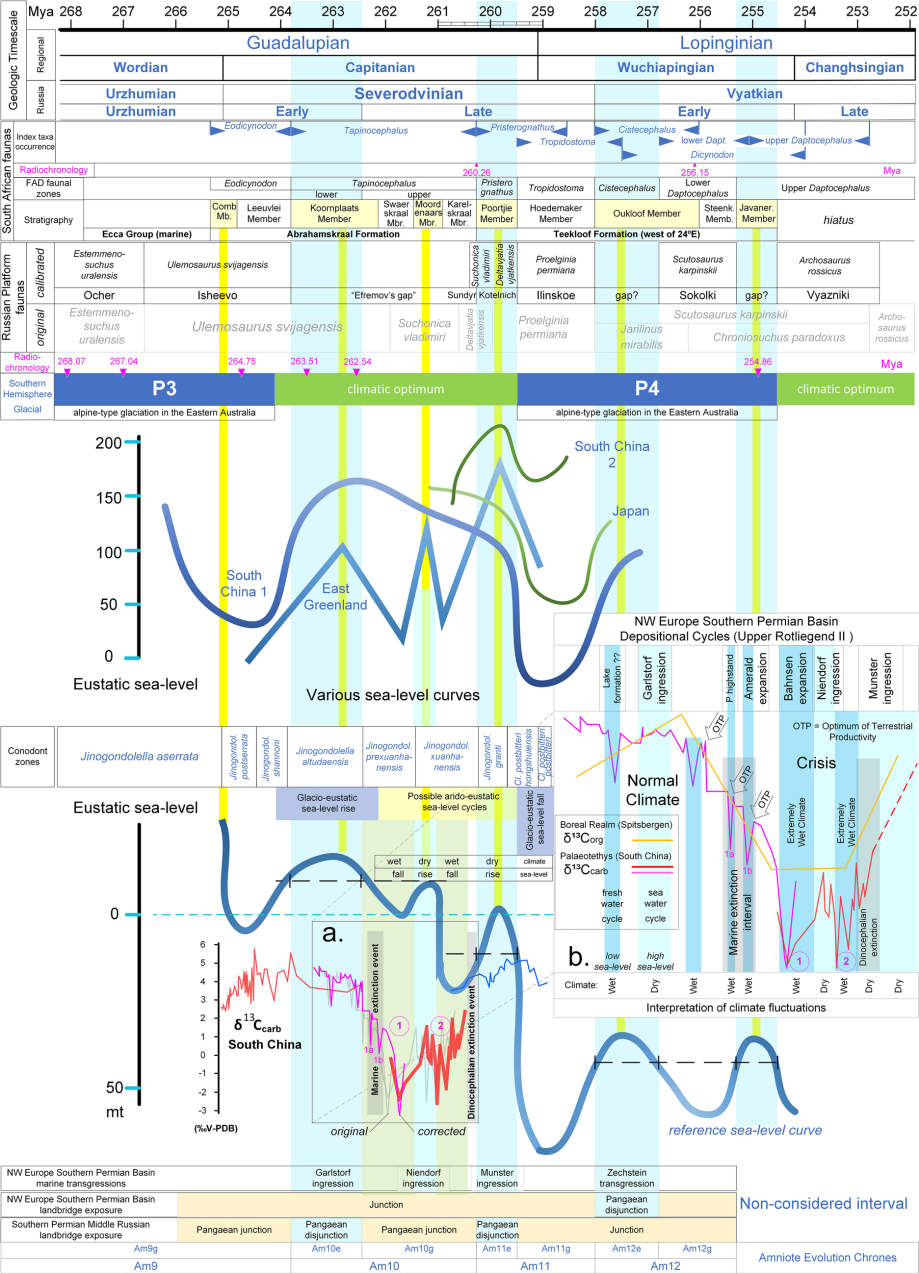
A middle-to-late Permian biochronostratigraphic chart composed from high precision, recently reported datasets. The thick blue sea-level curves are scaled, whereas the thinner green curves are not. Numbers in magenta correspond to radioisotopic dates. The carbon isotope-based interpretation of the climate and the pre-Zechstein depositional cycles (Upper Rotliegend Formation, Central European Basin; frame b) are in accordance with the arido-eustasy model. According to the arido-eustasy model, negative excursions in the carbonate carbon isotope (δ^13^C_carb_) and the terrestrial organic carbon isotope (δ^13^C_org-wood_) records correspond to a wet climate, falling sea-levels, and increasing levels of fresh water in terrestrial aquifers, whereas the increases in δ^13^C_carb_ and δ^13^C_org-wood_ correspond to a dry climate, rising sea-levels, and declining levels of fresh water in terrestrial aquifers.

The marine-like ostracod assemblages occur only within a narrow piedmont band of the marginal deflection, which has been traced from south to north for over 200 km^15^ and corresponds to the narrow seaway that connected the URS with the Palaeotethys from the southeast as shown in detailed Middle/Late Permian palaeogeographic reconstructions^7,13,32^. However, a palaeogeographic configuration of the previous lower Kazanian ingression suggested that an additional seaway from the southwest could have widened this connection^26^. In conclusion, two main transgressions in the Severodvinian broke the SLB leading to two Pangaean disjunction episodes. These two transgressions correlated temporally with the Garlstorf and Munster marine ingression events in the Upper Rotliegend II succession of the NW Europe Southern Permian Basin^33^, whereas the Niendorf ingression correlated with an intermediate transgression (of lower magnitude) that failed to break the SLB (see Fig. 2). As suggested by similarities in marine fauna, all of these ingressions penetrated the NW Europe Southern Permian Basin from the Arctic rift side only and without any Tethyan connection^33^.

The next major sea-level fall, which can be linked to the onset of the Australian P4 Alpine Glacial Period, reunited Pangaea. A break in the P4 glacial period, which was probably related to an abrupt warming period (well-recorded in various sections of the globe)^22,34^, created the Zechstein transgression. This transgression clearly established marine environments in the NW Europe Southern Permian Basin and succeeded the predominantly terrestrial environments of the Upper Rotliegend II succession^33^. The marine faunas with Tethyan similarities, such as foraminifera^35^, bivalves^36^, and conodonts (ref.^37^ and references therein), recovered from the Zechstein successions provide evidence for the exposure of a continuous south-north seaway from the Palaeotethys to the Arctic rift that divided the terrestrial continuity of Pangaea^14^. Although this vicariant event probably had more than one cycle, only the first cycle is considered here.

### Synthesis of the Middle Permian biochronostratigraphic chart

To define the complex palaeoenvironmental and palaeogeographic conditions of the upper Middle Permian, a state-of-the-art biochronostratigraphic chart was reconstructed based on correlations from recently available, highly precise data (Fig. 2). The synthesis of the chart is described below.

The last update (August 2018) of the ICS^17^ was used to provide a high-precision chronostratigraphic framework to evaluate radiochronological dates for biostratigraphic correlations. The sea-level fluctuation curve of Haq and Schutter^16^ was realigned stage by stage resulting in updated absolute ages for the sequence boundaries (sea-level lowstands). Sea-level curves for the upper Middle Permian from South China^38,39^, Japan^40^, and East Greenland^41^ were also aligned on the chart. The East Greenland curve was not originally dated well and was integrated after Legler and Schneider^33^ correlated it with the Upper Rotliegend marine ingressions. Accordingly, the corresponding transgressions are referred to by the names of the Upper Rotliegend marine ingressions (Garlstorf, Niendorf and Munster, see Fig. 2).

Annexation of the most detailed Capitanian δ^13^C_carb_ record available allowed for precise palaeoenvironmental interpretations under the recently proposed arido-eustasy model^42^. Of particular importance is the chronological definition of the upper boundary of the Russian Severodvinian Stage, which currently is not dated precisely^43^. Although this boundary is known to penetrate the Wuchiapingian Epoch of the regional timescale^43,44^, it is often erroneously displayed as coinciding with the upper boundary of the Capitanian Epoch (for example, see the Geological Timescale 2012^45^). Here, it is correlated with the onset of the Zechstein transgression dated at 258 Mya^46^.

### Synthesis of clado-stratigraphic patterns

#### Defining faunal index zones

The most up-to-date index ages of the fauna of the South African Platform and the Russian Platform, which are the areas with the most Palaeozoic vertebrate fossils, were aligned on the chart. Because this study was primarily focused on biogeographic geodispersal events, the first occurrence of a taxon was more important than the last occurrence. Therefore, regarding the well-dated South African faunal zones, the definition that better matched the criteria of the GIS-based biozone map of the Beaufort Group^47^ was adopted. The *Eodicynodon* zone was set at the onset of the marine regression that preceded the Garlstorf transgression because it corresponds exactly to the transition between the marine Ecca Group and the terrestrial Beaufort Group^48^. The boundary of the *Eodicynodon-Tapinocephalus* zones was previously set in the earliest Capitanian based on palynologic correlations and tetrapod ranges^49^, which matches perfectly with the definition used here. The *Tapinocephalus* zone was further divided into lower and upper subzones corresponding to the “Upper *Eodicynodon* Transition Zone” and the main *Tapinocephalus* zone (sensu ref.^50^). The boundary of the *Tapinocephalus-Pristerognathus* zones was set at 260.26 Mya based on the radioisotopic age of the dinocephalian extinction event^48,51^. The *Pristerognathus-Tropidostoma* boundary was set at 259.5 Mya on the basis of radioisotopic ages^52^ and the precise palynological age of the *Tropidostoma* First Appearance Datum (FAD)^48^. Currently, the bottom of the *Cistecephalus* zone is still based on the first appearance of *Aulacephalodon* and *Oudenodon*, although the FAD of the latter is no longer considered a good indicator, because it first occurs in the *Tropidostoma* zone (ref.^48^ and references therein). Hence, in the absence of detailed, palynologically correlated data for the distribution of *Aulacephalodon*, the onset of the *Cistecephalus* zone was set between the *Cistecephalus* FAD and the Last Appearance Datum (LAD) of the *Tropidostoma* zone^48^, which places it between 258 and 257.5 Mya. Finally, a recent proposal for the replacement of the terminal Permian *Dicynodon* zone with two (lower and upper) *Daptocephalus* zones^48,53^ was adopted because the two zones match very well with the Zechstein transgression-regression stages. Accordingly, the lower *Tapinocephalus*, *Pristerodon*, and *Cistecephalus* South African Assemblage Zones appear to be periods of endemic development during the transgressive (Pangaean disjunction) intervals, whereas the upper *Tapinocephalus*, *Tropisostoma*, and upper *Daptocephalus* zones appear to be regressive (Pangaean junction) geodispersal intervals (see Fig. 2).

Furthermore, an even more precise chronostratigraphic division was made within the *Eodicynodon* and *Tapinocephalus* zones based on correlations between the transgression intervals of the reference sea-level curve and deposition of the arenaceous members of the Teekloof^48^ and Abrahamskraal^50^ Formations. Because each of the arenaceous members corresponds to a particular transgression interval (Fig. 2), occurrences of fossilised vertebrates within these zones (previously described in stratigraphic detail^50^ can be dated with high precision.

The ages of fauna from the Russian Platform were reconsidered based on the most recent biostratigraphic charts^54,55^. In the absence of radioisotopic dates, the index faunal zones were initially aligned stage by stage onto the reference timescale and the time between the end of the Severodvinian and the end of the Capitanian was taken into consideration. In light of the very good match between the faunal ages and the transgression-regression occurrences, the next step was to correct slightly the intervals of the Russian fauna so that they coincided absolutely with the transgressions and the ages of the South African fauna (see Fig. 2). Thus, the hiatus between the Inta and Mezen/Isheevo Russian faunal assemblages^54^ corresponded to the early Kazanian transgression. “Efremov’s gap”^54^ covers the interval from the “Garlstorf” transgression to the occurrence of the Sundyr Fauna and is probably where the last dinocephalians existed before their permanent extinction^55^. The onset of the South African *Pristerognathus* zone coincides with the dinocephalian extinction in South Africa, which corresponds to the Russian *Deltavjatia vjatkensis* zone that characterises the Kotelnich Subassemblage^56^. Finally, the Russian *Proelginia permiana* zone characterising the Ilinskoe subassemblage, the *Scutosaurus karpinskii* zone characterising the Sokolki subassemblage, and the *Archosaurus rossicus* zone characterising the Vyazniki assemblage correlate very well with the South African *Tropidostoma* zone, lower *Daptocephalus* zone, and upper *Daptocephalus* zone, respectively.

**Figure 3:**
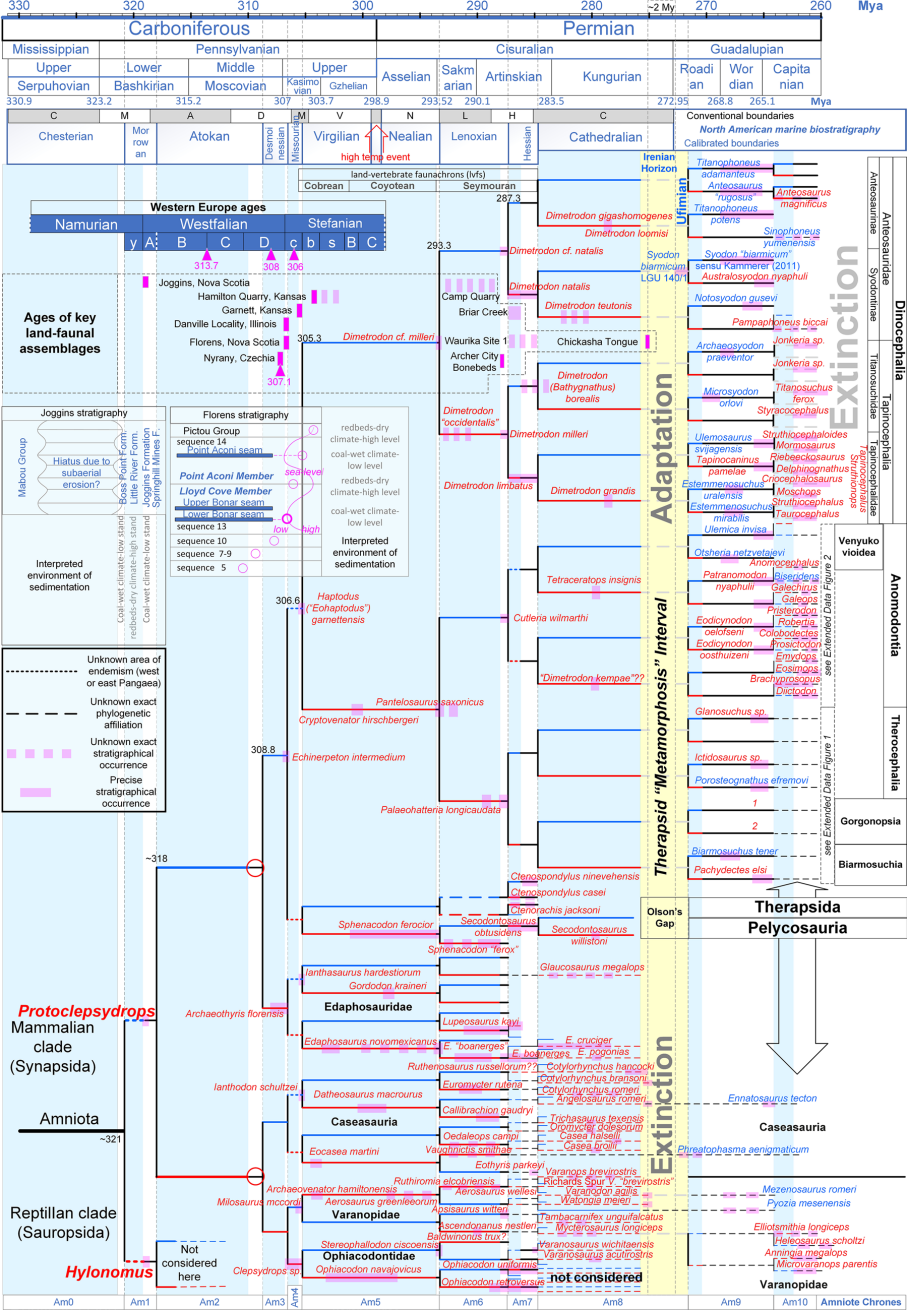
Vicariant clado-stratigraphic pattern of the early evolution of non-mammalian synapsids. According to the displayed vicariant clado-stratigraphic analysis, the 16 suddenly emergent therapsid branches are unlikely to be descended from lineages for which no representative has yet been recovered (as currently is widely believed). Instead, the therapsids should be descended directly from Kungurian pelycosaurian ancestors that evolved into therapsids because of remarkable environmental pressure during a narrow temporal window (2 My), which is here called the Therapsid "Metamorphosis” Interval. Note that the exact ancestor–descendant phylogenetic relationship between the pelycosaurian and therapsid groups is unknown, and the relationship displayed here is just indicative of the principle. The North American marine biostratigraphic zones were calibrated to the vicariant model (previously proposed ranges are shown by the white/grey bars on the top). Nomenclature of the Western European Namurian, Westphalian, and lower Stefanian stages: y: Yeadonian, A: Langsettian, B: Duckmantian, C: Bolsovian, D: Asturian, c: Cantabrian, b: Barruelian, s: Saberian. Taxa in red and blue have been recovered from western and eastern Pangaea, respectively.

**Figure 4:**
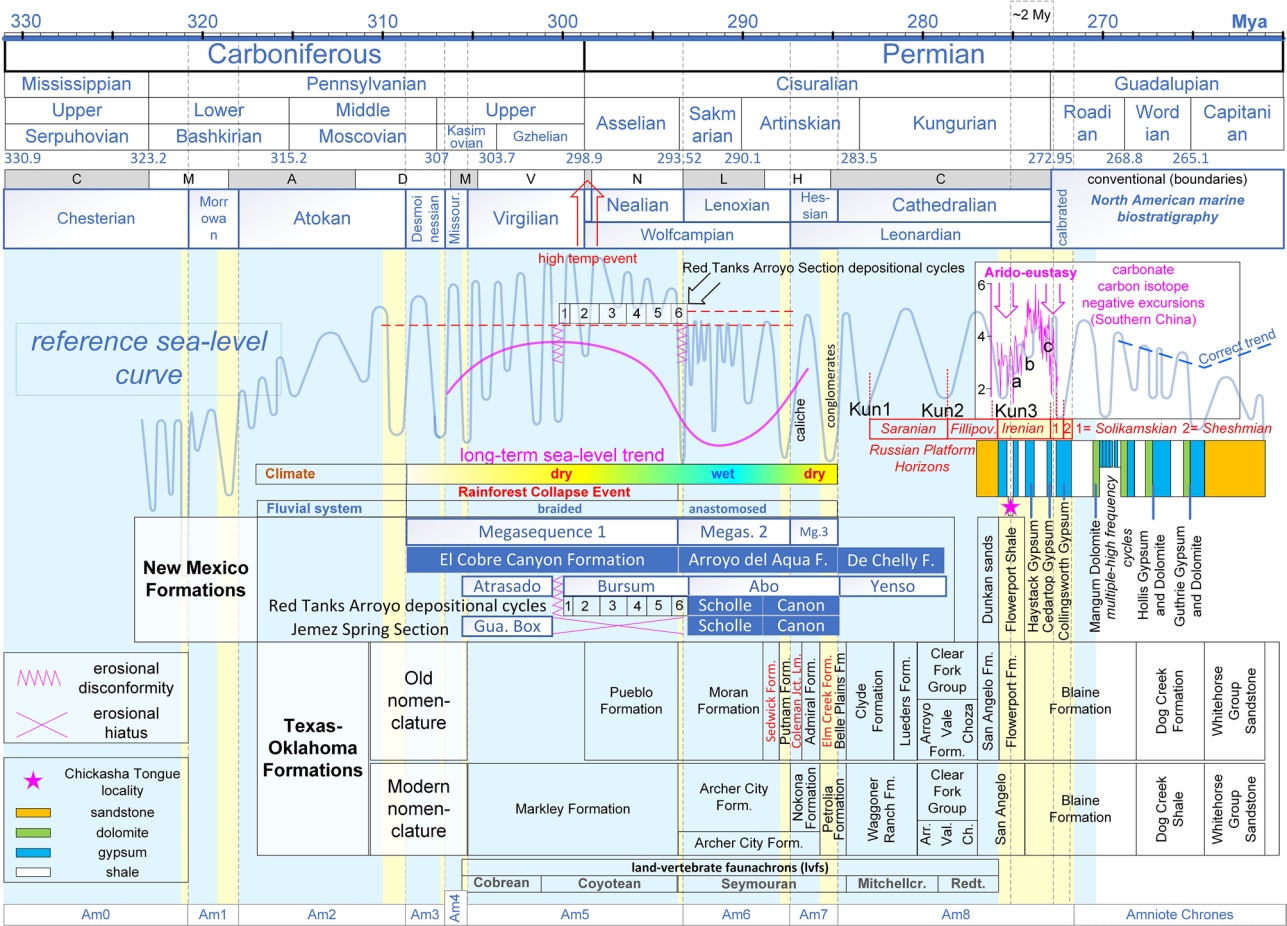
Correlation of the reference sea-level curve with the deposition cycles of various geologic formations. For explanations see “Methods” and the text.

The proposed early land-vertebrate faunachrons (lvfs)^54^ were aligned to the vicariant model (Figs. 3 and 4). The boundary between the Cobrean and Coyotean lvfs was set around the upper/lower Gzhelian as originally proposed^54^ and the Cobrean spans the Virgilian. The Coyotean/Seymouran boundary correlated precisely with the boundary of the El Cobre Canyon and Arroyo del Aqua formations as the lowest occurrence of the *Seymouran* is found within the Arroyo del Aqua Formation^54^ (Fig. 4). The upper end of the Seymouran lvf was set in the Late Artiskian and the earliest Cathedralian marine stage as originally proposed^54^.

#### Dating of the most important land-vertebrate faunal assemblages

Precise dating of the land-vertebrate faunal assemblages is critical for placing the recovered early synapsid taxa upon the correct branch of the vicariant model. The ages of several key faunal assemblages were reconsidered and updated as necessary on the basis of two lines of evidence (Fig. 3). The first line of evidence was the recently reported precise radioisotopic ages for the stages of the Westfalian Western European age, which were extracted from the fossiliferous Nýřany Member in the Czech Republic^57^, and these new ages differed from the previously assumed ages. The second line of evidence was the calibration of the conventional intervals of the North American marine biostratigraphic units^58–60^ upon the vicariant model. Because each biostratigraphic unit corresponds to an evolutionary step among the marine faunas, they were initially aligned on the branches of the vicariant model to test whether the SLB affected evolution of the marine biota, and it was found that the vicariant model accounted for the observed pattern in the North American marine biostratigraphic units. Thus, the units were calibrated on the branches of the vicariant model based on the following rule: as each unit characterises a novel faunal composition, the units should correspond to the marine geodispersal intervals during the periods when sea-levels were high and the role of the SLB as a marine biogeographic barrier was eliminated.

Accordingly, the Nýřany faunal assemblage^61^ found within the Nýřany Horizon in the Nýřany Member of the Kladno Formation^62^ and considered to be of Asturian (Westphalia D) age^63^ was precisely dated at 307.1 Mya^57^.

Fossils from the Joggins locality in Nova Scotia^64,65^ were sampled from the Joggins Formation deposited in the Cumberland sub-basin, which was originally located at least 1,000 km from the open sea^66^. Nevertheless, the sub-basin was connected to open marine waters by a narrow strait during marine highstand phases, which affected the water level and the alkalinity of the flooding surfaces^67^. A regional erosional unconformity underlying the base of the Boss Point Formation is believed to be due to subaerial erosion from a major sea-level lowstand caused by Gondwana glaciation^68^. The Joggins Formation is of early Langsettian (earliest Westphalian) age, whereas the underlying Little River and Boss Point Formations are of latest Namurian (Yeadonian) age^67^. Based on the reference sea-level curve, the age of the regional unconformity at the base of the Boss Point Formation and the magnitude of its erosional hiatus suggest that the unconformity correlates with the remarkable Bash 3 sea-level lowstand. Accordingly, deposition of the Joggins Formation correlates with the Bash 5 lowstand (Figs. 1 and 3).

The Florens fossiliferous locality of Nova Scotia is younger than the Joggins locality as it was deposited above the Lower Bonar coal seam within the Lloyd Cove Member of the Sydney Mind Formation^69,70^, which is considered to be of Cantabrian (early Stefanian) age^71^. In particular, the Lower Bonar coal seam was deposited just after a sea-level lowstand (lower level of sequence 13 in ref.^71^) and was succeeded by a sea-level highstand. A second lowstand occurred during the deposition of the overlying Point Aconi Member (sequence 14 in ref.^71^), which is also of Cantabrian age. Therefore, based on the reference sea-level curve, the Florens fauna correlates with the Kas 1 lowstand and the next Point Aconi lowstand correlates with the Kas 2 sequence boundary (Figs. 1 and 3).

The vertebrate fossils of the Danville locality in Illinois have been collected from a zone ~ 3 m below the Shoal Creek Member (equivalent to the Carthage Limestone Member) of the Bond Formation^72^. The Carthage Limestone Member falls within the earliest Missourian^73^. In Kentucky, the boundary of the Middle-to-Late Pennsylvanian can be set just below the Carthage Limestone Member^74^. These data support the precise definition of the base of the Missourian stage proposed in Fig. 3 and suggest that the Danville locality is contemporaneous with the Florens locality in Nova Scotia.

The fossil assemblage of the Hamilton Quarry in Kansas is thought to be of Stefanian^75^, middle Virgilian (Shawnee Group) age^76^, although some researchers assign it to the Late Kasimovian stage^77,78^. Indeed, after calibration of the North American biostratigraphic units, the lower Virgilian falls within the Late Kasimovian, supporting the Late Kasimovian age.

The fossiliferous sediments at the Garnett locality in Kansas were found upon the Rock Lake Shale Member, which represents the bottom level of incised channels formed through subaerial erosion during a period of marine regression^79^. Detailed geologic analysis showed that the channels are part of the great Tonganoxie palaeovalley that was formed during a remarkable sea-level lowstand^80^. The most recent data indicate that the palaeovalley incision started just before the deposition of the Tonganoxie Sandstone Member (Stranger Formation),the latter filled the palaeovalley at the Missourian/Virgilian boundary^76^. These precise data correlated certainly the age of the Garnett fossils to the Kas 2 sequence boundary of the reference sea-level curve (Figs. 1 and 3).

The fossiliferous section in the Waurika Site 1 locality in Oklahoma is thought to be Leonardian in age based on Olson’s^81^ interpretation that it is part of the Wellington Formation. Correlation of the position of the Waurika Site 1 (Fig. 3 in ref.^81^) with the modern geologic map of the area^82^ shows that the Waurika Site 1 area was dominated by complex sedimentation between the Leonardian Wellington Formation and the latest Wolfcampian Oscar Group^54^. Therefore, the age range of the Wellington Formation proposed by Olson (Fig. 1 in ref.^81^) is incorrect. In particular, Olson^81^ reported that the fossils were excavated from grey shale lying ~ 12 m below the base of the “Ryan-Asphaltum Sandstone” horizon. Later investigations showed that the “Ryan-Asphaltum Sandstone” horizon was actually two non-contemporaneous sandstone units: the Ryan Sandstone that constitutes the base of the Wellington Formation and the Aphaltum Sandstone that constitutes the base of the overlaid Garber Formation^83^. Because there are no depositions of the Garber Formation in the Waurika Site 1 proximal area (see the map in ref.^82^), the “Ryan-Asphaltum Sandstone” horizon described by Olson^81^ most likely corresponds to the Ryan Sandstone. This view is supported by the fact that on the geological map^84^ used by Olson^81^, the “Ryan-Asphaltum Sandstone” zone in the Waurika Site 1 area coincides with the range of the Oscar Group on the modern geologic map^82^. Therefore, the fossils of Waurika Site 1 should be correlated with the upper Oscar Group, which is equivalent to the upper Archer City–Petrolia Formations^54^, and should be considered mid-Artiskian in age.

In the Cutler Group strata of New Mexico, the age of the boundary between the El Cobre Canyon Formation (Late Pennsylvanian-Early Permian) and the overlying Early Permian Arroyo del Agua Formation is not precisely known^85,86^. However, because the succession of the two formations has been interpreted to correspond to a depositional change of fluvial systems, correlation of this transition to a link of eustatic sea-level, fluvial system deposition and long-term climate change may allow for a more precise dating of the boundary. In particular, three megasequences were previously found to dominate the deposition of the Cutler Group: the basal Megasequence 1 characterises a braided fluvial system dominated primarily by mudstone deposits, Megasequence 2 characterises an anastomosed fluvial system dominated by ribbon-like sandstones, and Megasequence 3 characterises a return to a braided fluvial system^86,87^. Megasequence 1 has been correlated with the El Cobre Canyon Formation and Megasequence 3 has been correlated with the upper Arroyo del Agua Formation^88^. Given the great distance of the depositional position (Chama Basin) from the sea at the time of sedimentation^89^, sea-level change can be excluded as a possible factor in the depositional change of the fluvial systems (downstream-controlled fluvial systems). Rather, the depositional change of the fluvial systems may have been driven by tectonic cycles superimposed on a longer-term climatic background or by climatic cycles superimposed on a steady tectonic regime (upstream-controlled fluvial systems)^90^. The former hypothesis is favoured by some authors^86,87^, however, evidence from modern, equivalent riverine systems in Central Australia, which are dominated by an arid climate that matches the ancient climate of the Chama Basin, favours the latter hypothesis. In arid Central Australia, the change from the mud-dominated braided fluvial system to the ribbon-like sandstones that characterise the active anastomosing channels results from the system moving large volumes of water and moderate sediment loads across low-gradient interior basins^91^. In other words, the fluvial system transition corresponds to a long-term increase in the hydrologic seasonal budget during the flooding season.

According to the arido-eustasy^42^ and the aquifer-eustasy^92,93^ models of sea-level change, dry global climates seem to correspond to sea-level rises and wet global climates correspond to sea-level falls. Accordingly, the long-term high sea-level trend that characterised the Carboniferous–Permian transition could coincide with a long-term dry period; this may have caused the reported rainforest collapse event (Fig. 4). Interestingly, the rule that fluvial cycles that are controlled by climate changes are completely out of phase relative to those driven by base-level changes^90^ was also independently confirmed. Consequently, correlations of high and low sea-level intervals with intervals of anastomosed and braided fluvial systems can be used to specify the boundary between the El Cobre Canyon Formation and the overlying Arroyo del Agua Formation (see the long-term sea-level curve in Fig. 4). Furthermore, the return of the braided fluvial system characterising Megasequence 3 and the top of the Arroyo del Agua Formation correlated with the onset of the dryer period, which characterised the deposition of the adjacent and contemporaneous Wellington Formation in Oklahoma^94^. Accordingly, the age of the Camp Quarry faunal assemblage, which was previously estimated to fall within the Megasequence 2 interval^95^, is now assigned to Amniote Chrone 6 (Figs. 3 and 4).

The oldest specimens of the genus *Dimetrodon* are from the upper Bursun Formation in New Mexico^96^,these have been classified as *Dimetrodon cf. Dimitrodon milleri*, and were recovered from the sixth and uppermost depositional cycle of the mid-to-late Virgilian-middle Wolfcampian (Asselian^97^) Bursum Formation in the Red Tanks Arroyo section. Although, the exact age of these important fossils is unknown, they can be dated satisfactorily on the basis of correlations between depositional cycles and the reference sea-level curve. Lithologic analysis of the Bursun Formation suggests that there were six depositional cycles (Fig. 5 in ref.^98^ and Fig. 5 in ref.^97^). Figure 4 shows that all of these depositional cycles correlate very well with the cycles of the reference sea-level curve, and the long-term sea-level curve (magenta colour) correlates very well with the long-term sea-level trend that characterised the marine and fresh-water/terrestrial facies in the sequence of the Attrasado, Bursun, and Abo Formations^99^. Furthermore, the major unconformities that characterise the base and the top of the Bursum Formation^100,101^ can be explained by the two contemporaneous major sea-level falls in the reference sea-level curve (Fig. 4). Accordingly, the *Dimetrodon cf. Dimitrodon milleri* specimens were deposited during the Sak 1 sea-level lowstand (Fig. 3).

The ages of the north-central Texas formations were updated based on the recent temporal definition of the Coleman Junction, the top of which correlates with the Wolfcampian-Leonardian boundary^102^. After the North American marine biostratigraphic zones were calibrated to the vicariant model, the Wolfcampian-Leonardian boundary correlated with the end of the Art 2 sea-level lowstand. However, given that the formations of Northeast Texas are terrestrial (Wichita Group), the boundaries of the formations of the Lower Wichita Group can only be correlated precisely with the Art 2 lowstand using the equivalent marine formations in South Texas. Thus, according to Hentz et al.^102^, the deposition of the Coleman Junction Limestone Member corresponds to a transgression system that followed a lowstand between depositional cycles 16 and 17. This lowstand appears in the middle of two regressive sandstones that were deposited between the Coleman Junction Limestone and the Sedwick Limestone (see Table 2 in ref.^102^). Accordingly, in the Geological Atlas of Hentz and Brown^103^, the interval of the Art 2 sea-level lowstand correlates very well with the Sandstones SS8 and SS6 of the Archer City Formation (formerly the Putnam Formation). Therefore, all of the fauna excavated south of the Archer City Formation (Bonebeds 1, 2, 3, and 4)^104^ falls within the Art 2 sea-level lowstand. Because, currently, there is no equivalent sequence stratigraphic analysis for the interval above the Coleman Junction, the interval of the former Elm Creek Formation^103^ can be correlated with the Art 3 sea-level lowstand. This hypothesis explains the occurrence of the striking heterozoan carbonate system within the Elm Creek Limestone Member^105^ and the development of the prerequisite cold-water conditions which coincided with the Art 3 sea-level lowstand and the P2a glacial period (Fig. 1). Accordingly, the transgression that followed that glacio-eustatic lowstand correlated with the subsequent exposure of the former Belle Plain Formation (Fig. 4).

Of particular importance is defining the age of the San Angelo Formation and the overlain Blaine Formation, which are placed chronologically just before the appearance of the therapsids (Figs. 3 and 4). Although a late Leonardian age can safely be suggested, in the absence of good marine trace fossils, it is difficult to determine the age of those formations with great precision^106^. Therefore, the characteristic evaporitic depositional cycles of the interval between the Dunkan Sandstone of the lower San Angelo Formation and the Whitehorse Group Sandstone (Fig. 3 in ref.^107^) were correlated with the reference sea-level fluctuation curve. Because the evaporitic depositional cycles are characterised by alternating gypsum, shale, and dolomite depositions, the correlation was made according to the following rules: the evaporitic gypsum deposition corresponds to sea-level lowstands,the shale deposition corresponds to sea-level highstands,and the dolomite deposition corresponds to the regression of system tracts (due to diagenesis forced by the progressive increase of fresh water in the underground aquifers. Thus, according to Reference Section C (Fig. 3 in ref.^107^), the first two gypsum layers in the Flowerpot Shale correlated with the Kun3 and the Kun3a lowstands, whereas Kun3b correlated with the base of the Blaine Formation (Figs. 3 and 4). Because the Chickasha Tongue and the Chickasha Formation are equivalent to the middle Flowerpot Formation^108,109^, their important fossiliferous layers can be also be dated very well using this approach.

Given that transgression/regression (T/R) cycles create distinct depositional layers, by evaluating the reference sea-level curve and by corresponding each horizon to a T/R cycle, the controversial chronostratigraphy of the Russian Platform horizons of both the Ufimian and the global Kungurian stage^46^ could be defined with better accuracy (Figs. 1, 3 and 4). Thus, the marine Lower Solikamskian Horizon represents a narrow window of time close to the Kungurian-Roadian boundary (and most likely within the Roadian), whereas the Sheshmian Horizon falls clearly in the early Roadian as previously suggested^54^. This correlation is strengthened by the correlation between the depositional environmental data of the Ufimian Horizons^110^ and the arido-eustasy model. Thus, the arid/wet cycles of the Irenian Horizon seem to have culminated in the maximum transgression and aridity represented by the marine Lower Solikamskian Horizon, whereas the succeeding regression (Roa1 lowstand) likely corresponded to a wet climate mode, which matched the terrestrial Upper Solikamskian Horizon. Thus, the aquatic environment of the Inta faunal assemblage from the Russian Platform appears to be Upper rather than Lower Solikamskian^111^ in age. Similarly, correlation of the Sheshmian Horizon with the succeeding transgressive interval (Figs. 1 and 4) corresponded to a return to an arid climate as proposed by recent palaeoenvironmental interpretations^112^.

#### Constructing the clado-stratigraphic trees

The current representative taxa were aligned with the vicariant topology based on their geological age and their taxonomic evaluation in published phylogenetic analyses (see the Supplementary Information). Importantly, the taxa included were from taxonomic groups with sufficient representative lineages in terms of density and validity in recent phylogenetic analyses. In cases in which different authors presented different taxonomic views, an effort was made to find as much consensus as possible with the help of the vicariant topology. Taxa that occur in multiple localities were documented mainly on the basis of data available in online databases such as the Paleofile (https://www.paleofile.com/) and the Paleobiology Database (https://paleobiodb.org/#/). The ages of the relevant localities were reconsidered and updated as needed according to the data presented here.

The various taxa were arranged on the clado-stratigraphic trees using the following rule: given an assumed absolute geographical isolation between the eastern and western Pangaean biogeographic territories, the fossilised taxa recovered from disjunction (high sea-level) intervals can be exclusively attributed to the branch of the Pangaean territory where they were discovered (periods of endemism), whereas the taxa recovered from the junction intervals can be attributed to either of the two Pangaean territories (geodispersal periods). Therefore, during the disjunction intervals, the number of lineages characterising a specific taxonomic level and geologic time should be less than or equal to the number of available vicariant branches at that taxonomic level and geologic time. Any conflict with that rule may indicate a fault in the vicariant model, a fault in the dating of the taxon, and/or a fault in the phylogenetic evaluation of the taxon. The possibility of the latter case is lower when the conflict involves taxa that are well represented at the genus level (e.g. when two candidate genera claim a single vicariant branch). However, species-level conflicts might occur for various reasons, such as the occurrence of chronospecies that belong in the same chronocline.

Construction of the clado-stratigraphic trees requires robust phylogenies for the examined lineages. Although such phylogenies are available for many early synapsid groups, they are lacking for other groups. For example, because the *Ophiacodon* species have never been considered in a formal phylogenetic analysis, only the older representatives (*O. navajovicus, O. uniformis* and *O. retroversus*) were considered here. The same is true for the tapinocephalid dinocephalians. In a preliminary report, Güven and colleagues^113^ noted that the diversity of tapinocephalid dinocephalians in the *Tapinocephalus* Assemblage Zone was overestimated and should be re-examined, thus they reduced the number of recognised genera of tapinocephalids from 18 to 9; a complete report of their results is still pending.

### Palaeoenvironmental data

The occurrence of several faunal assemblages during periods of low sea-level coincides with distinct intervals of wet climate (reflected by coal deposition) that punctuated longer periods of relatively dry climate (reflected by red beds deposition). In particular, at the Joggins locality in Nova Scotia, the deposition of the lower Boss Point Member upon a regional unconformity (correlated here with the Bash 3 lowstand) signals the transition from a dry climate to a wet climate, which is evidenced by the presence of coal and thick sandstone deposits from large river systems (ref.^65^ and references therein; Fig. 3). In the upper Boss Point Member, the presence of calcareous paleosols and red floodplain deposits and the absence of coal and limestone beds mark the beginning of semi-arid conditions that continue into the overlying Little River Formation. This dry interval corresponds to a sea-level highstand. Subsequently, the Joggins Formation, which contains extensive coal and limestone beds reflecting wetland conditions, was deposited during a period of low sea-level and wet climate (ref.^65^ and references therein). In the younger Florens locality of Nova Scotia, the fossiliferous Lower Bonar and Upper Bonar coal seams (Lloyd Cove Member) were deposited during a wet climate^71^ (correlated here with the Kas 1 lowstand). Red beds were deposited over the Lloyd Cove Member before another coal seam was deposited in the Point Aconi Member (correlated here with the Kas 2 lowstand) again signalling a transition to a wet climate^114^. Above that level, the Pictou Group consists of more than 300 m of red beds without coal^71^, which corresponds to a long-lasting transgression interval that coincides with the rainforest collapse event at the Carboniferous–Permian transition^115^ (Figs. 3 and 4).

Thus, as recently proposed by the aquifer-eustasy^92,93^ and the arido-eustasy^42^ models of sea-level change, dry global climates correspond to sea-level rises and wet global climates correspond to sea-level falls (due to excess accumulation of fresh water in continental aquifers).

Building on the aquifer-eustasy model, the arido-eustasy model posits that extreme (> 1.5‰) and abrupt negative excursions in the marine carbon isotope record (δ^13^C) of sediments containing organic matter from terrestrial vegetation (δ^13^C_org-wood_) resulted from orbitally forced periods of extremely wet climate. Such hydroclimatic intensification, in turn, has led to environmental crises, sea-level falls, and carbon cycle perturbations, which are reflected by negative excursions in δ^13^C_carb_ values of marine sediments^42^. During this stage, marine biotic extinctions occurred through perturbation of the chemical, physical, and biological properties of the oceans.

As long as the dynamic of a changing climate is smoothed and stabilized in a wet mode (although temporarily), uptake of the light carbon isotope from the atmosphere for terrestrial photosynthesis is increased and primary terrestrial productivity is maximised. This leads to a decrease in the atmospheric carbon isotope concentration (δ^13^C_air_), which then causes the δ^13^C_DIC_ to fall because of air-sea CO_2_ exchange between the oceans and the atmosphere^116^. Thus, the net continental deposition of carbon leads to a characteristic positive culmination in the δ^13^C_carb_ value of marine sediments, which is called the Optimum of Terrestrial Productivity (OTP)^42^. As the orbital anomaly develops and the climate becomes wetter, whole biomes adapt to higher water use,the δ^13^C_org-wood_ values further decrease and the δ^13^C_carb_ values display new negative excursions and new OTPs.

Under the terms of the arido-eustasy model, the poorly understood hydrological cycles of the Upper Rotliegend Formation of the NW Europe Southern Permian Basin^33^ can be explained and dated (see Fig. 2b). Although there is currently no detailed contemporaneous δ^13^C_org-wood_ record available to confirm the arido-eustasy model during this period, a contemporaneous marine organic carbon isotope (δ^13^C_org_) record from Svalbard (the source of the organic material, marine or terrestrial, is not known^117^) displays a negative excursion (yellow line in Fig. 2b) during the same interval. Under the arido-eustasy model, a climatic crisis is not expected to affect δ^13^C_org_ values of purely marine organic material, thus the Svalbard record is assumed to consist predominantly of terrestrial material.

## Results

### The palaeogeographic model

Given the wide terrestrial connection between the URS and rest of Pangaea, exposure to the SLB and the associated sea-level fluctuations^16^ were the only factors contributing to the vicariance pattern in the Pangaean junction-disjunction model (Fig. 1).

The rhythms and magnitudes of the oscillations in the reference sea-level curve match the junction-disjunction topology of Pangaea very well with the exception of the negative long-term trend during the Capitanian. The Capitanian correlated with a warm period and the end of the P3 glacial period^22^, which would have led to a global sea-level rise, and based on this correlation, the validity of the long-term regressive trend in the reference sea-level curve seems doubtful. The alternative trend proposed in Fig. 2 is more likely because it is in agreement with the precise occurrences of the P3 and P4 glacial periods, which were only recently dated with high accuracy. This alternative trend is also supported by recent global mantle flow models, which suggest that the Late Permian sea-level curves were affected by subsidence through dynamic topography changes^118^.

The strong resemblance between the “conventional” intervals of the North American marine biostratigraphic zones and the branches of the vicariant model suggests that the new species of marine invertebrates that characterised each zone resulted from vicariance due to the effects of the SLB (Fig. 3). Only the Newwellian zone (at the Virgilian/Nealian boundary) was found to be unaffected by SLB-related vicariance. Rather, the Newwellian zone reflects the prominent extension of the high latitude range of the Tethyan fauna during the high temperature event at the boundary of the Carboniferous/Permian period^119^ and the subsequent amphi-Pangaean distribution of the fauna when the climate cooled.

### The phylogenetic patterns

By fitting the vicariant model on the taxonomically and stratigraphically evaluated non-mammalian synapsid lineages that are sufficiently represented in the fossil record (see the Appendixes S1 and S2 in Supplementary Information), clado-stratigraphic patterns were identified (Fig. 3 and Fig. S1 and S2 in Supplementary Information). Examination of the clado-stratigraphic patterns shows that there is no numerical conflict between the predictions of the vicariant model and the currently recovered fossilised taxa (at the generic level). For example, the vicariant model predicts that a maximum of eight synapsid genera should be recoverable from the western Pangaean Garnett locality, and seven genera have been recovered from there to date (Fig. 3). On the other hand, any intrageneric variation expressed through the occurrence of different species in the same vicariant branch could be attributed to anagenesis of a single lineage (chronospecies) or to a misunderstanding of intraspecific morphological variation (e.g. a case of nomen dubium).

A slight but noteworthy discrepancy is the occurrence of tapinocephalid dinocephalian genera in the *Tapinocephalus* Assemblage Zone (Amniote Chrone 10 in Fig. 3); the vicariant model predicts a maximum of eight taxa, but 11 taxa are currently described in the literature (see the Supplementary Information). However, Güven and colleagues^113^ concluded that the diversity of tapinocephalid dinocephalians in the *Tapinocephalus* Assemblage Zone is overestimated and that the taxonomy should be re-examined in a formal phylogenetic study. Therefore, until such a study is conducted, the model cannot be considered inconsistent with the tapinocephalid dinocephalians.

Because the vicariant model fits closely with the temporal rhythm of emergence of various synapsid taxa, vicariance due to Pangaean separation appears to be the cause of vertebrate speciation during the separation period. Current knowledge of evolution of the amniote and amphibian clades^120,121^ suggests that the amniote-amphibian split occurred ~ 350 Mya. This split may be attributed to an early ephemeral terrestrial connection between Laurussia and Gondwana, which preceded the permanent tectonic contact between Laurussia and Gondwana ~ 323 Mya^9^ (Fig. 1 in this study and Fig. 9.1a in reference^6^). Fitting of the oldest known reptile and synapsid fossils, *Hylonomus* and *Protoclepsydrops*, respectively^122^, onto the vicariant model indicates a precise divergence date of 321 Mya for the reptile-mammal split. Both fossils are from the Langsettian (Westphalian A) age and from the Joggins locality in Nova Scotia, which correlates perfectly with the Bash 3 lowstand with regards to timing and duration (Figs. 1 and 3). Although *Hylonomus* and *Protoclepsydrops* developed separately in the preceding transgression interval, they geodispersed to both sides of Pangaea during the lowstand, and thus, have been found together on the West Pangaean side (Joggins locality, North America). This is a characteristic example of how synapsid biogeographic distribution can be interpreted under the proposed vicariance model.

## Discussion

### Biogeographic perspective

The URS separated Pangaea into eastern and western biogeographic regions. Although most of the supposed early East Pangaean lineages (blue lines in the clado-stratigraphic patterns) have not been recovered from the East Pangaean fossil record (see Figs. S3 to S8 in Supplementary Information), the concordance between the vicariant palaeogeographic model and the phylogenetic patterns suggests that all of the vertebrate lineages were able to disperse to both sides of the URS. The view of a continuant Pangaean bioregion finds support from a recent study of the changes in beta diversity of Palaeozoic tetrapods^123^ that rejects previous suggestions^78^ that the rainforest collapse might have caused an “island biogeography” effect (see also ref.^124^).

**Figure 5:**
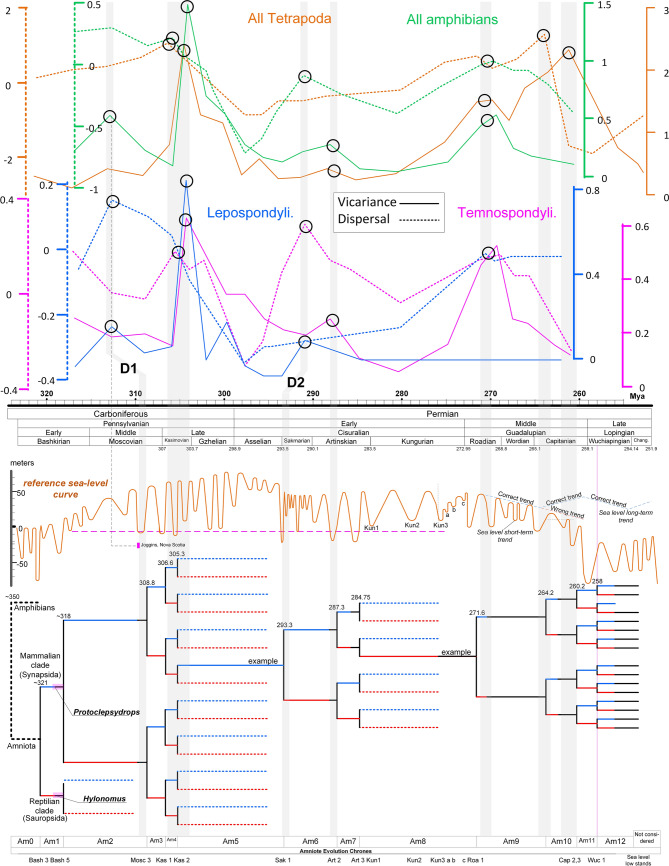
Correlation of the vicariant model of this study with the independent results of another vicariant-dispersal analysis^125^. Circles denote congruence between the junction-disjunction events concluded in the current study and the dispersal-vicariance events of ref.^125^ (indicated by the positive graph peaks). D1 and D2 are temporal deviations from the congruence (grey vertical bars), probably owing to different adopted absolute ages, especially for the Langsettian age of the high fossiliferous site of Joggins, Nova Scotia (D1) and the various North American faunas in general (D2).

When the supposed East Pangaean lineages were found in the West Pangaean territory during sea-level lowstands, they usually displayed a primitive evolutionary status (e.g. *Eothyris* and *Glaucosaurus*) and morphological differences that distinguished them at the genera level (e.g. *Stereophallodon*, *Lupeosaurus*, and *Bathygnathus*). Further, these lineages contributed to an abrupt and previously unexplained increase in the diversity of the fossil record (e.g. the upper *Tapinocephalus* Assemblage Zone of South Africa). This sudden increase in diversity may be attributed to the geodispersal interval and the favourable (wet) climatic conditions that dominated the periods during which the sea-level lowstands and geodispersals occurred. Indeed, this view finds support in the results of a recent dispersal-vicariance analysis on Late Palaeozoic tetrapods^125^, which are in a good agreement with the topology of the current vicariant model (see Fig. 5). That study, however, has attributed the obtained vicariance to orogenic activity and increasing climate heterogeneity, instead of the sea-level change suggested here.

The agreement between the predictions of the vicariant model and the distribution of taxa recovered from the fossil record suggests that primitive vertebrates were not able to cross the URS, a relatively narrow marine barrier, via “sweepstake” dispersals. Thus, most of the strange biogeographic conclusions regarding “sweepstake” transoceanic dispersals of terrestrial vertebrates elsewhere (e.g. trans-Atlantic^126^), which resulted from poorly understood palaeogeographic evolution and/or inaccurate dates for clade divergences due to incorrectly calibrated molecular clocks, should be reconsidered. The results presented here strongly support the conclusion that allopatric speciation through vicariance was the predominant mode of vertebrate evolution^1,2^.

### Taphonomic perspectives

The proposed vicariance model is characterised by an apparent lack of documentation of the postulated East Pangaean lineages, as there is a striking absence of relevant pelycosaurian records from the east side of the SLB (see Fig. S8 in Supplementary Information). Reasonably, one could ask whether this is a case of taphonomic bias or a result of a real absence of pelycosaurians from the East Pangaea. Because sloping Pangaean areas underwent erosion and weathering, terrestrial fossils should be recovered mostly from areas of cratonic basins that were usually covered by epicontinental seas but became exposed during sea-level lowstands. Thus, the apparent absence of early synapsids from the Russian Platform can be explained by the rarity of such narrow regressive intervals before the latest Early Permian, whereas the discoveries of synapsids on the Russian Platform since the Middle Permian resulted from the onset of longer terrestrial exposures (see Figs. 1, 2, and 3). In accordance with this conclusion, a tight relationship between observed richness and sampling was inferred by a study that reassessed early tetrapod diversity and biogeography for spatial and temporal sampling biases^124^. Another study, by testing the early tetrapodomorph biogeography for geographic sampling bias in macroevolutionary and phylogenetic analyses, showed that the association between high formation counts in specific regions and high paleobiodiversity in those regions is likely not a coincidence and has a clear impact on how we interpret dispersal history^127^.

The report of cf. *Dimetropus* trace fossils from the North Caucasus provides indirect evidence for the presence of non-therapsid synapsids in the SLB area^128^. The next question is whether there are currently a sufficient number of Late Carboniferous–Early Permian East Pangaean sites with representative fossilised fauna from which pelycosaurian fossils are missing. The answer is no, as the only known fossil sites containing tetrapod fauna from this time period are the latest Early Permian (early Ufimian) Inta Fauna in Russia^111^ and the Early Permian fauna of Junggur basin in China^129^. Both of these sites contain only continental aquatic fauna. Thus, the current pelycosaurian fossil record of the East Pangaea should be considered as biased, because the number of fossil sites is small and represent habitats that are not typical for pelycosaurians. Heterogeneous sampling of aquatic-dominated and terrestrial-dominated faunal assemblages was recently suggested^130^ to have been the general trend characterizing the Carboniferous-Permian transition accompanied by a west-to-east Pangaean gradation of aridity,however, the area eastwards of the SLB (eastern Pangaea) was not considered in that study.

### Phylogenetic perspectives

The vicariant topology presented here helps explain the phylogenetic evolution of vertebrates and resolves some of the previous deadlocks in cladistic analyses. For example, the vicariant model supports the recent conclusions that therapsids descended from sphenacodont ancestors, and that edaphosaurians are a sister clade to sphenacodonts^131,132^. Furthermore, the model suggests that the reason the early non-therapsid synapsid (pelycosaurian) tree is not well resolved is that two key lineages of *Protoclepsydrops* descendants, which are predicted to be found together within the Mosc 3 sequence boundary, are currently absent from the fossil record (red circles in Fig. 3). On the other hand, the ophiacodontids are not older than the other pelycosaurians as has long been supposed^133^, but rather they maintain the ancestral ophiacodondian status that characterised all of the earliest pelycosaurians after the evolution of *Protoclepsydrops*. Also, Reisz^134^ associated the *Protoclepsydrops* with the Ophiacodontia. Thus, *Archaeothyris* is currently considered to be the most basal ophiacodontid^13^ because it is the most ancient well-known pelycosaur and is evolutionarily very near the two *Protoclepsydrops* descendants.

The phylogenetic trees developed using the vicariant model do not conclusively resolve phylogenetic relationships, as alternatives are possible, but they do demonstrate the phylogenetic topology under which evolution occurred. For example, the remarkable correspondence between the pelycosaurian and therapsid clado-stratigraphic patterns shown in Fig. 3 suggests that the advanced therapsids should be descended directly from known Kungurian pelycosaurian ancestors and not from more derived, yet-unrecovered (ghost) lineages, as is widely believed. This indicates that great phenotypic change took place over just two million years within a period referred to here as the Therapsid “Metamorphosis” Interval. This falls within a hiatus in tetrapod evolution known as Olson’s gap^135^. In light of the traditional phylogenetic beliefs, this is a controversial scenario, which can be validated only by future fossil discoveries. Therefore, it would be worthwhile to search for the ancestors of the therapsids and their intermediate morphological stages within particular stratigraphical layers, such as the uppermost Flowerport Formation and the lowermost Blaine Formation of Texas, and the Irenian and Solikamskian Horizons in the Russian Platform (see Fig. 4). During the Therapsid “Metamorphosis” Interval, the pelycosurian taxa that adapted to assume a therapsid-like posture by extending their four limbs vertically beneath the body survived (*Haptodus’s* descendants), whereas most of the other reptile-like pelycosaurian taxa with sprawling posture went extinct (e.g. ophiacodontids, caseids) (Fig. 3). Clearly, some exceptional evolutionary change led to that event and is discussed below.

### Palaeoenvironmental interpretations

The occurrence of several faunal assemblages during the periods of low sea-level coincides with distinct intervals of wet climate (reflected by coal deposition) that punctuated longer periods of relatively dry climate (reflected by deposition of red beds) (Figs. 3 and 4). Thus, as recently proposed by the aquifer-eustasy^92^ and arido-eustasy^42^ models of sea-level change, dry global climates correspond to sea-level rises, and wet global climates correspond to sea-level falls (due to excess accumulation of fresh water in continental aquifers). Such 1.4 to 1.6 million years Milankovitch-forced sedimentary cycles correlating very well with the eccentricity oscillations of earth's orbit^136^ and the oscillations of the reference sea level curve (see Fig. 1).

An analogous observation can be made in the fossiliferous Karoo Basin in South Africa where the deposition of the distinct arenaceous Poortjie Member has been linked to increased aridification, reduced vegetation cover, and increased weathering of the source region (ref.^48^ and references therein). Because the occurrences of the Poortjie Member and the other arenaceous members of the Teekloof Formation and the Abrahamskraal Formation coincide very well with the transgression intervals (see the yellow strips in Fig. 2), they indicate a link between an arid climate and marine transgression. In particular, the Poortjie Member coincides with a transgression interval that was preceded by cycles of sea-level change that cannot be explained by glacio-eustasy (climatic optimum zone in Fig. 2). On the other hand, the cycles can be explained very well in terms of arido-eustasy on the basis of carbonate carbon isotope (δ^13^C_carb_) data (see Fig. 2).

In the arido-eustasy model of palaeoenvironmental change, an orbitally forced transition to a remarkably wet climate mode initially leads to natural selection favouring plant genotypes that are adapted for higher water use. This is reflected in a decrease in the δ^13^C_org-wood_ value (~ 2%) of plant organic material within marine organic sediments of predominantly terrestrial origin. Dynamically changing climate caused extreme weathering of inland areas and transfer of excessive terrestrial organic material and nutrients into the stratified Palaeozoic and Mesozoic epicontinental seas. This resulted in a strong drop in the relative concentration of dissolved inorganic carbon (δ^13^C_DIC_) (through a decline in the ^13^C isotopic content of DIC relative to ^12^C). The repeated occurrences of Violent Hydroclimate Perturbations (VHP) caused euxinic ocean conditions and biotic crises in both marine and terrestrial ecosystems, with the former occurring primarily during the transition to a wet climate mode and the latter occurring during the transition to an arid mode (Brikiatis in preparation).

Indeed, in Fig. 2a, the calibrated δ^13^C_carb_ graph from South China^137^ shows that the two main δ^13^C_carb_ negative shift intervals (circles 1 and 2) corresponded to sea-level falls, and that a marine extinction event known as the Middle Permian extinction event^117,137^ occurred during the first two δ^13^C_carb_ negative excursions (1a and 1b; ~ 2%) of the first δ^13^C_carb_ negative shift (circle 1). Accordingly, Fig. 2 shows that the Middle Permian marine extinction event and the dinocephalian extinction event^51^ do not chronologically coincide and, in fact, under the arido-eustasy model, occurred for opposite reasons: the former was due to marine euxinia caused by an extremely wet climate, whereas the latter was due to the transition to a dryer climate that dramatically reduced the vegetation cover. This in turn deprived the predominantly herbivorous dinocephalians of food and habitat. Indeed, in South Africa the dinocephalian extinction was contemporaneous with the deposition of the distinctly arenaceous Poortjie Member, which is linked to increased aridification, reduced vegetation cover, and increased weathering of the source region (references in ref.^48^). More recently, stable oxygen and carbon isotope compositions of dentine apatite from 28 specimens of the dicynodont therapsid *Diictodon feliceps* independently demonstrated that the extinction coincided with a positive excursion of δ^13^C values (in agreement with Fig. 2) and an unusual increase of aridity but not of temperature^138^. Further evidence for the remarkable aridity is the floral extinction event observed in the contemporaneous middle Upper Shihhotse Formation in North China, in which 56% of the plant species were lost^139^.

Furthermore, environmental perturbations due to arido-eustasy cycles may also explain the exceptional Therapsid “Metamorphosis” Interval (Fig. 3), which coincides with the sabkha facies of the Irenian Horizon in the Russian Platform and the unique sequence of the four, small-to-moderate magnitude, T/R cycles (Kun3, Kun3a, Kun3b and Kun3c) of the reference sea-level curve (see Figs. 1 and 4). Under the arido-eustasy model, all of those cycles are interpreted as successive wet/arid cycles leading progressively to aridity. This interpretation is in accordance with the floral extinction event in North China in which 45% of the plant species were lost in the contemporaneous Lower Shihhotse Formation^139^. In addition, contemporaneous δ^13^C_carb_ negative excursions have been recorded for the Tieqaio section in Laibin in the Guangxi Province of North China^140^ (see Fig. 4). Further research on this important interval is needed, because the currently available geochemical studies do not include samples of terrestrial organic material and, therefore, cannot provide evidence regarding the arido-eustasy model.

### Biochronostratigraphic perspectives

A new, unified biochronostratigraphic unit – the Amniote Chrone – is proposed here for amniote evolution and biogeography. The Amniote Chrones can be further subdivided into endemism (Ame) and geodispersal (Amg) Chrones.

## Conclusions

During the Late Palaeozoic, the Pangaea supercontinent was characterised by fragmentation episodes that divided its biogeographic continuity through sea-level fluctuation. The resultant vicariance affected the evolution of both terrestrial vertebrates and marine invertebrates with consistency.

Detailed vicariance models can help resolve the divergence times and phylogenetic relations of vertebrate evolution. They can also account for the observed diversity in the vertebrate fossil record. In particular, the vicariant analysis of this study suggests that the advanced therapsid vertebrates descended directly from Kungurian pelycosaurian ancestors. The mysterious therapsid “metamorphosis” interval in which the primitive reptile-like pelycosaurian lineages developed into the mammalian-like therapsids lasted only two million years and remains unexplored within a specific, thin stratigraphical horizon. Although these phylogenetic conclusions contradict traditional phylogenetic hypotheses regarding the origin of the therapsids, the vicariant analysis provides exact forecasts of where and when the missing synapsid lineages are likely to be recovered. Thus, its validity can be directly tested in the near future.

Environmental perturbations due to orbitally forced arido-eustasy cycles may also explain the mid-Permian biotic extinction events and depositional cycles, such as the pre-Zechstein of the Central European Basin.

## Supplementary information

Supplementary Information.
