# Supplementary material for: An early Pangaean vicariance model for synapsid evolution
Source: Sci Rep. 2020 Aug 4;10:13091. doi: 10.1038/s41598-020-70117-8 (PMC7403356; doi:10.1038/s41598-020-70117-8)
Supplement: Supplementary file 1 — Supplementary Information. [file 41598_2020_70117_MOESM1_ESM.docx]

SUPPLEMENTARY INFORMATION

**An early Pangaean vicariance model for synapsid evolution**

**Author:** Leonidas Brikiatis^ᵃ^

ORCID iD: https://orcid.org/0000-0002-9157-523

**Affiliation:**

^ᵃ^non-affiliated

Correspondence to: [lb@aegeanman.com](mailto:lb@aegeanman.com)

Address: Ioannou Fix 5, 17564, Palaeo Faliro, Greece.

**Index**

1. **Appendix S1:** Pelycosaurian stratigraphy and phylogenetic relations.
2. **Appendix S2:** Early therapsid stratigraphy and phylogenetic relations.
3. **Figure S1:** Vicariant clado-stratigraphic pattern of the synapsid groups Gorgonopsia, Biarmosuchia, and Therocephalia.
4. **Figure S2:** Vicariant clado-stratigraphic pattern of the synapsid group Anomodontia.
5. **Figure S3:** Distribution map of Gorgonopsia.
6. **Figure S4:** Distribution map of Biarmosuchia.
7. **Figure S5:** Distribution map of Therocephalia.
8. **Figure S6:** Distribution map of basal anomodonts.
9. **Figure S7:** Distribution map of anomodont clade Dicynodontia.
10. **Figure S8:** Distribution map of Early Permian Sphenacodonts.

**Appendix S1: Pelycosaurian stratigraphy and phylogenetic relations**

**Synapsida, Pelycosauria**

**Amniote chrone 1 (Am1)**

1. ***Protoclepsydrops haplous***

**Where:** Joggins locality, Canada [1].

**When:** Joggins Formation, Westfalian A (see the Methods).

**Phylogenetic relation:** Crown synapsid [2, 3].

**Amniote chrone 3 (Am3)**

1. ***Archaeothyris florensis***

**Where:** Northern Hemisphere:

1. Florens locality, Nova Scotia, Canada (*Archaeothyris florensis*).
2. Nýřany locality, Czech Republic (*Archaeothyris sp.*).
3. Linton locality, Ohio, USA (*Archaeothyris sp.*) [4].

**When:**

1. Florens locality, earliest Missourian (see the Methods).
2. Nýřany locality, latest Westphalian D (301.7 Ma) (see the Methods).
3. Linton locality, immediately below the Upper Freeport Coal Member (ref. [4]: Ohio link). The precise age of the Upper Freeport Coal Member is the latest Westphalian D [5].

**Phylogenetic relation:** *Archaeothyris florensis* has been shown to be the most basal ophiacodontid by several phylogenetic studies [6, 7, 8, 9, 10] (but see the Discussion).

1. ***Echinerpeton intermedium***

**Where:** Florens locality, Nova Scotia, Canada [11].

**When:** Florens locality, earliest Missourian (see the Methods).

**Phylogenetic relation:** A morphologically intermediate between the sphenacodontians and the ophiacodontians [3]; ophiacodontid [7, 9]; ophiacodontid or a stem sphenacomorph [8].

1. ***Clepsydrops coletti***

**Where:** North America:

1. Florens locality, Nova Scotia, Canada (*Clepsydrops sp.*).
2. Danville Locality, Illinois, USA (*C. collettii*; *C. vinslovii*) [12].

**When:**

1. Florens locality, earliest Missourian (see the Methods).
2. Danville Locality, earliest Missourian (see the Methods).

**Phylogenetic relation:** *Clepsydrops* has long been considered to be related to the ophiacodontids [13]. However, the fragmentary nature of the *Clepsydrops* remains has not allowed this relationship to be established in a formal phylogenetic study.

**Notice:** Three species of *Clepsydrops* have been reported so far: *C. collettii*, *C. vinslovii* [13], and *C. magnus* [14]. *C. vinslovii* is probably a junior synonym of *C. collettii* [15, and references therein].

**Amniote chrone 4 (Am4)**

1. ***Clepsydrops magnus***

**Where:** Casselman Formation, Pennsylvania, USA [12].

**When:** *C. magnus* was excavated from sandstone just above the Ames limestone [14], which constitutes the uppermost member of the Glenshaw Formation and the boundary with the overlaid Casselman Formation. This boundary is considered to be of the earliest Virgilian age [5]. Stratigraphically, the *C. magnus* deposition corresponds to the 9^th^ 400-Kyr cyclotherm, whereas the *C. collettii* and *C. vinslovii* (Danville Locality) correspond to the 5^th^ cyclotherm of ref. [5]. Therefore, their time difference is ~1.5 Ma.

**Phylogenetic relation:** *Clepsydrops* has long been considered to be related to the ophiacodontids [13]. However, the fragmentary nature of the *Clepsydrops* remains has not allowed this relationship to be established in a formal phylogenetic study.

**Notice:** The younger age of the *C. magnus* suggests that it may be a chronospecies of the *Clepsydrops* lineage.

1. ***Ianthodon schultzei***

**Where:** Garnet locality, Kansas, USA [16].

**When:** Garnet locality, Missourian/Virgilian boundary (see the Methods).

**Phylogenetic relation:** A taxon with sphenacodont affinities, but found outside Sphenacomorpha (Edaphosauridae and Sphenacodontia) [7, 17] and near Caseasauria [6].

1. ***Milosaurus mccordi***

**Where:** Falmouth Locality, Jasper County, Illinois, USA [18].

**When:** Mattoon Formation (McLeansboro Group) [18], Missourian age [19].

**Phylogenetic relation:** *M. mccordi* was considered to be a varanopid by DeMar [18]. Spindler et al. [9] concluded that is the sister taxon to *Varanosaurus acutirostris* within the Ophiacodontidae. However, another recent phylogenetic study that exclusively examined the relations of *M. mccordi* [7] concluded that *M. mccordi* is sister to *Ianthodon schultzei*, and this is the classification adopted in this study.

1. ***Xyrospondylus ecordi***

**Where:** Garnet locality, Kansas, USA [20].

**When:** Garnet locality, Missourian/Virgilian boundary (see the Methods).

**Phylogenetic relation:** A poorly represented taxon with some edaphosaurian affinities, however, it is possible that it is a non-synapsid [17]. Thus, it is not included in the current phylostratigraphic trees.

1. ***Haptodus garnettensis* (*= “Eohaptodus” garnettensis*)** [17]

**Where:** Garnet locality, Kansas, USA [21].

**When:** Garnet locality, Missourian/Virgilian boundary (see the Methods).

**Phylogenetic relation:** *Haptodus garnettensis* was redefined as *Eohaptodus garnettensis* [17] and is considered to be a basal sphenacomorph (e.g. ref. [9, 17]).

1. ***Ianthasaurus sp.***

**Where:** USA [22]:

1. Garnet locality, Kansas, USA (*Ianthasaurus hardestiorum*).
2. Fremont County, Colorado (*Ianthasaurus*).

**When:**

1. Garnet locality, Missourian/Virgilian boundary (see the Methods).
2. Fremont County, Sangre de Cristo Formation, Missourian. The recognized age of this locality is based on its vertebrate assemblage (ref. [22]: Colorado link).

**Phylogenetic relation:** *Ianthasaurus* is considered to be the most basal edaphadaurian [6, 7, 8, 17, 23].

1. ***Kenomagnathus scotti***

**Where:** Garnet locality, Kansas, USA [17].

**When:** Garnet locality, Missourian/Virgilian boundary (see the Methods).

**Phylogenetic relation:** According to Spindler [17], *Kenomagnathus scotti* is a poor representative sphenacomorph with a questionable phylogenetic position. Thus, it is not included in the current phylostratigraphic trees.

1. ***Tenuacaptor reiszi***

**Where:** Garnet locality, Kansas, USA [17].

**When:** Garnet locality, Missourian/Virgilian boundary (see the Methods).

**Phylogenetic relation:** According to Spindler [17], *Tenuacaptor reiszi* is a poor representative sphenacomorph with a questionable phylogenetic position. Thus, it is not included in the current phylostratigraphic trees.

**Amniote chrone 5 (Am5)**

1. ***Ophiacodon navajovicus***

**Where:** USA [24]:

1. Cañon del Cobre, New Mexico, USA.
2. Halgaito Tongue horizon, Utah, USA.

**When:**

1. Cañon del Cobre, (El Cobre Canyon Formation), Cobrean lvf [25].
2. Halgaito Tongue horizon [26] corresponds to the Halgaito Formation, which is of the latest Virgilian-early Wolfcampian age [27].

**Phylogenetic relation:** The oldest member of the family Ophiacodontidae.

1. ***Stereophallodon ciscoensis***

**Where:** Clay County, Texas, USA [28].

**When:** *Stereophallodon ciscoensis* was first described by Romer [28] and the exact position of its fossiliferous location was described by Romer [29: “locality 24”]. Accordingly, the fossils were recovered from the red clay beneath the sandstone horizon around the boundary (and probably down from the boundary) of the Moran-Pueblo Formations. However, based on the modern Geologic Atlas of Texas [30], “locality 24” (about 6.5 km south-southeast of Windthorst) can be placed definitively above the boundary of the Moran-Pueblo Formations (Markley-Archer Formations boundary in the updated nomenclature), which is within the earliest Archer Formation (formerly Moran). Therefore, correlation with the chart in Fig. 4 assigns *Stereophallodon* an age around the Asselian–Sakmarian boundary (see also ref. [6]).

**Phylogenetic relation:** It has been concluded that *Stereophallodon* is the sister taxon to *Ophiacodon* [6, 7, 8, 31].

1. ***Archaeovenator hamiltonensis***

**Where:** Hamilton Quarry, Kansas, USA [32].

**When:** Hamilton Quarry, lower Virgilian, Late Kasimovian (see the Methods).

**Phylogenetic relation:** *Archaeovenator* is the most basal and oldest member of the family Varanopidae [9, 32, 33].

1. ***Aerosaurus greenleeorum***

**Where:** Cañon del Cobre, New Mexico, USA [13, 28]

**When:** Romer and Price [13] noted that the specimen was collected from the “lower level” (the floor) of the Cañon del Cobre without any other specific information. The “lower level” probably corresponds to the El Cobre Canyon Formation [34]. Thus, the age may range from the Gzhelian to the Asselian (Fig. 4).

**Phylogenetic relation:** *A. greenleeorum* is a member of the family Varanopidae. Spindler et al. [9] concluded that the genus *Aerosaurus* is paraphyletic with *A. greenleeorum* more closely related to *Ruthiromia elcobriensis* than *Aerosaurus wellesi*, thus, it was suggested that these taxa be renamed*.* Fig. 3 shows that their closer relationship is very likely due to their proximal age. *A. greenleeorum* is most likely a chronospecies between *Archaeovenator* and *Ruthiromia elcobriensis*. Thus, based on currently available data regarding the stratigraphical occurrence and the phylogenetic relations of these related taxa, the following ancestor-descendant relation is concluded here: *Archaeovenator hamiltonensis* - *Aerosaurus greenleeorum* and *Ruthiromia elcobriensis* - *Aerosaurus wellesi*.

1. ***Eocasea martini***

**Where:** Hamilton Quarry, Kansas, USA [35].

**When:** Hamilton Quarry, lower Virgilian, Late Kasimovian (see the Methods).

**Phylogenetic relation:** *E. martini* is the most basal and oldest member of Caseasauria [8, 9, 35].

1. ***Datheosaurus macrourus***

**Where:** Nowa Ruda, Poland [36, and references therein].

**When:** Ludwikowice Formation, Stephanian C [36, and references therein].

**Phylogenetic relation:** *Datheosaurus* belongs to Caseasauria [8, 9].

1. ***Edaphosaurus mirabilis***

**Where:** Kounov, Czech Republic [37].

**When:** Kounov Member, Slany Formation [37]. The Kounov Member is deposited in the uppermost Slany Formation and is dated at the Stefanian B (Saberian)/C boundary (301.6 Ma) [38].

**Phylogenetic relation:** *E. mirabilis* is known only from a single vertebra [13], thus its exact phylogenetic relation is questionable. It is not included in the current phylostratigraphic trees.

1. ***Edaphosaurus colohistion***

**Where:** Elm Grove locality, West Virginia, USA [39].

**When:** Upper Pittsburgh Formation of the Monongahela Group [39], which is of the late Virgilian age [5, 40].

**Phylogenetic relation:** *E. colohistion* is known only from a series of 14 presacral vertebrae. It is undoubtedly considered an edaphosaurid, however, its taxonomic position is problematic because “it shares no apomorphies with other Texan edaphosaurids”, such as the contemporaneous *E. novomexicanus* and the younger *E. boanerges* [23, 41). Thus, it is considered here as a chronospecies of *E. novomexicanus*.

1. ***Edaphosaurus novomexicanus***

**Where:** New Mexico, USA [42].

**When:**

1. Cañon del Cobre (El Cobre Canyon Formation), Cobrean lvf [25].
2. Northern Sacramento Mountains (*Edaphsaurus cf. novemexicanus*), Bursum Formation and lowermost levels of the Coyote Hills Member (very close to the base of the Abo Formation [43, and references therein]), earliest Sakmarian (see Fig. 4).

**Phylogenetic relation:** *E. novomexicanus* is considered to be a basal edaphosaurid related to the most basal edaphosaurian, the *Ianthasaurus* [7, 23, 41). Romer and Price [13] noted that without good cervical vertebrae material, it is difficult to distinguish *E. novomexicanus* from *E. boanerges*. Therefore, younger specimens that have been referred to as *Edaphosaurus cf. Edaphsaurus novemexicanus* could fall within the taxon *E. “boanerges”* (see Amniote Chrone 6).

1. ***Cryptovenator hirschbergeri***

**Where:** Rhineland Palatinate, Germany [44].

**When:** Middle Remigiusberg Formation [44]. The middle Remigiusberg Formation corresponds to the boundary of the *Autunia conferta- Sphenophyllum angustifolium* macroflora biozones [45], which falls within the boundary of Stefanian B and C that is dated at 300.4 Ma [38].

**Phylogenetic relation:** A sphenacodontid more closely related to the *Dimetrodon* and *Sphenacodon* [6], the *Cryptovenator* [44], or the *Secondotosaurus*, *Cutleria* [8], and *Ctenorhahis* [7].

1. ***Macromerion schwartzenbergii***

**Where:** Kounov, Czech Republic [46].

**When:** Kounov Member, Slany Formation [46]. The Kounov Member was deposited in the uppermost Slany Formation and was dated at the Stefanian B (Saberian)/C boundary (301.6 Ma) [38].

**Phylogenetic relation:** Romer [47] concluded that it is a typical sphenacodont with a maxilla very similar to *Dimetrodon*. However, due to the fragmentary nature of the remains, he did not assign it to a specific genus. *Macromerion* has never been included in a formal phylogenetic study. Thus, it is not included in the current phylostratigraphic trees.

1. ***Neosaurus cynodus***

**Where:** Les Gorges, Franche-Comté Region, France [48, and references therein].

**When:** Unnamed stratigraphic unit of the late Gzhelian–Asselian age [48, and references therein].

**Phylogenetic relation:** Falconnet [48] reviewed the data for *N. cynodus* and considered it a nomen dubium of an indeterminate sphenacodontid. Therefore, it is not further considered here.

1. ***Pantelosaurus saxonicus***

**Where:** Döhlen Basin, Dresden, Germany [50, and references therein].

**When:** Six fully articulated *Pantelosaurus* skeletons were found in a single slab of sediment deposited just above the uppermost or first seam of the Döhlen Formation [17, 49, 50]. Recently, the Unkersdorf Formation underlying the Döhlen Formation was aged at 294 ± 3 Ma and 293 ± 5 Ma [51] suggesting that the horizon of *Pantelosaurus* correlates with the Sak1 sea-level lowstand (Asselian–Sakmarian boundary; see Fig. 1 and 3).

**Phylogenetic relation:** *Pantelosaurus* is considered to be a stem spenacodontid [17, 44] and/or a stem therapsid [6, 7, 8, 52], which is closely related to *Eohaptodus* (“*Haptodus”*) *garnettensis* [6, 7, 8, 52] and *Palaeohatteria longicaudata* [17, 44, 50]. Although Romer and Price [13] placed *Pantelosaurus* and *Palaeohatteria* within the genus *Haptodus*, they noted that both are very closely related to the genus *Dimetrodon*.

1. ***Hypselohaptodus (“Haptodus”) grandi*** [17]

**Where:** Kenilworth area, West Midlands, England (ref. [17] and references therein).

**When:** The fossils were found within the Kenilworth Sandstone Formation in a layer of Asselian age (ref. [17] and references therein).

**Phylogenetic relation:** *H. grandis* has been redefined based on the specimen Gz 1071, an isolated maxilla, which was previously attributed to *Haptodus baylei* (ref. [17] and references therein). According to Spindler [17], *H. grandis* is a poor representative sphenacomorph with a questionable phylogenetic position. Thus, it is not included in the current phylostratigraphic trees.

1. ***Sphenacodon britannicus***

**Where:** Kenilworth area, West Midlands, England [53].

**When:** The fossils were found together with *Hypselohaptodus (“Haptodus”) grandi* within the Kenilworth Sandstone Formation of Asselian age [53].

**Phylogenetic relation:** Spielmann et al. [54] concluded that current data cannot distinguish the material referred to *S. britannicus* from the other *Sphenacodon* species. Thus, this taxon is not further considered here.

1. ***Sphenacodon ferocior***

**Where:** New Mexico, USA [55].

**When:**

1. Cañon del Cobre, (El Cobre Canyon Formation), Cobrean lvf [25], Virgilian [56].
2. San Diego Canyon, Sandoval County, Cantalupe Box Formation [57], Virgilian [56].

**Phylogenetic relation:** *S. ferocior* has been analysed primarily at the genus level in several phylogenetic studies, and it has been shown to be closely related to *Ctenospondylus* [58, 59], *Dimetrodon* [6, 7: however, in the absence of *Ctenospondylus*], both *Ctenospondylus* and *Dimetrodon* [17, 44], and *Cryptovenator* and *Cutleria* [8].

1. ***Dimetrodon cf. milleri***

**Where:** NMMNH locality 4640, central New Mexico, USA [60].

**When:** The sixth depositional cycle of the upper Bursun Formation correlated with the Sak 1 sea-level lowstand at the Asselian–Sakmarian boundary (see the Methods).

**Phylogenetic relation:** These *Dimetrodon* specimens were assigned to the *Dimetrodon cf. Dimitrodon milleri* taxon based on the dimensions of two of the preserved specimens [60].

1. ***Gordodon kraineri***

**Where:** Locality 8967, Otero County, New Mexico, USA [61].

**When:** Basal Bursun Formation, early Asselian [61] (see Fig. 4).

**Phylogenetic relation:** A specialized edaphosaurid with characteristics distinct from all of the other edaphosaurid genera [61].

**Amniote chrone 6 (Am6)**

1. ***Sphenacodon ferox***

**Where:** New Mexico and Utah, USA [54].

**When:** Upon reviewing the data for the two currently accepted species of the genus *Sphenacodon* (*S. ferocior* and *S. ferox*), Spielmann et al. [54] acknowledged the morphologically identical postcrania and the few, subtle cranial differences between the two species, which some others have interpreted as merely reflecting size and/or ontogeny. Nevertheless, the existence of two species was determined on the basis of the most complete *S. ferox* skull known (NMMNH P-55367), which provided new and critical information. However, NMMNH P-55367 was recovered from the Arroyo del Agua Formation, which is of Seymuran faunal age [56]. Currently, it appears that the two *Sphenacodon* species existed during the intervals of the Coyotean and Seymuran lvfs.

**Phylogenetic relation:** *S. ferox* has been analysed primarily at the genus level in several phylogenetic studies, which identified close relationships with *Ctenospondylus* [58, 59], *Dimetrodon* [6, 7: however, in the absence of *Ctenospondylus*], both *Ctenospondylus* and *Dimetrodon* [17, 44], and *Cryptovenator* and *Cutleria* [8].

1. ***Cutleria wilmarthi***

**Where:** Placerville Locality, Colorado, USA [62].

**When:** *C. wilmarthi* was described based on the holotype USNM-22099 and the specimen NCZ-2987 [62]. The former was found 80-90 feet beneath the top of the upper beds of the Cutler Formation, whereas the latter was found 100-200 feet deep. The upper beds (~1000 feet) of the Cutler Formation are considered to be of Wolfcampian age [63], including the upper Wolfcampian [64]. Therefore, the age of the *Cuttleria* horizon (uppermost Cutler Formation) can be considered of upper Wolfcampian (Lenoxian) age. Based on the vicariance model described here (Fig. 1 and 3), it is proposed to be of the uppermost Lenoxian age, which correlates with the Art2 sea-level lowstand.

**Phylogenetic relation:** *Cutleria* is considered to be a sphenacodontid [6, 8], a stem sphenacodontid [17, 44, 52], or a stem therapsid [58].

1. ***Palaeohatteria longicaudata***

**Where:** Döhlen Basin, Dresden, Germany [50, and references therein].

**When:** It was found within the lacustrine Limestone Member in the uppermost Niederhäslich Formation [65]. The Niederhäslich Formation correlates with the *Melanerpeton pusillum*–*M. gracile* zone of the Permo-Carboniferous European Amphibian biostratigraphic unit [66] of mid-Sakmarian age [67]. However, recently reported radioisotopic dates from the Döhlen Basin [51] suggest an early Artiskian age (~289 Ma).

**Phylogenetic relation:** *Palaeohatteria* is considered to be a stem spenacodontid closely related to *Pantelosaurus saxonicus* [17, 44] and *Eohaptodus* (“*Haptodus”*) *garnettensis* [44].

1. ***Haptodus baylei***

**Where:** Les Télots, eastern Autun Basin, France (ref. [17], and references therein).

**When:** Within the Black shales of the Millery Formation at Les Télots, which are considered to represent a lacustrine depositional environment [17]. The major lake horizons in the Millery Formation are considered to be of early Artiskian age (see the correlation chart of ref. [68]).

**Phylogenetic relation:** Currently, the exact age and the phylogenetic position of *Haptodus baylei* are not well defined [17]. Thus, it is not further considered here.

1. ***Dimetrodon milleri***

**Where:** Texas, USA:

1. Locality IIIf of Romer and Price [13], later known as Archer City Bonebed 1 [69].
2. Locality IIIb (“Fireplace”) of Romer and Price [13], locality 4 of Romer [70], locality 10 of Romer [29].
3. Locality IIa of Romer and Price [13].
4. Archer City Bonebed 2 [69], locality 11 of Romer [29].
5. Table Branch, locality IIIe of Romer and Price [13].

**When:**

1. Archer City Bonebed 1 [69], former Putnam Formation, mid-Artiskian age (see Fig. 4).
2. “Fireplace”. Based on the map in Romer [70] correlated with the Geological Atlas of Hentz and Brown [30], this site falls within the former Putnam Formation, mid-Artiskian age (see Fig. 4).
3. Locality IIa: one mile northwest of Padgett, Young County. Based on the Geological Atlas of Hentz and Brown [30], this site probably falls within the former Putnam Formation, mid-Artiskian age (see Fig. 4).
4. Archer City Bonebed 2, former Putnam Formation, mid-Artiskian age (see Fig. 4).
5. Table Branch, locality IIIe of Romer and Price [13], former Putnam Formation, mid-Artiskian age (see Fig. 4).

**Phylogenetic relation:** It is considered to be the stratigraphically oldest known species of *Dimetrodon* [58] and the most primitive [13]. Based on different tooth morphologies, it has been concluded that *D. milleri* from the Archer City Bonebed is a distinct species from *D. limbatus* and *D. grandis* [58].

1. ***Dimetrodon occidentalis***

**Where:** New Mexico, USA [71].

**When:** Jemez Springs, lower Abo Formation, Assemblage C [57], Sakmarian age (see Fig. 4).

**Phylogenetic relation:** As acknowledged by Berman [71], *D. occidentalis* and *D. milleri* are almost identical with regards to their dimensions (see the tables in ref. [72]). *D. occidentalis* differs from *D. milleri* only in the structure of the vertebral neural spines, which are unique among the *Dimetrodon* species. However, as demonstrated here, *D. occidentalis* is quite a bit older than *D. milleri*, thus the morphological differences could be interpreted as differences among chronospecies. Therefore, the older *Dimetrodon* cf. *milleri, D. occidentalis*, and *D. milleri* are considered to be from the same lineage (see also *Dimetrodon cf. limbatus* below).

1. ***Dimetrodon cf. limbatus***

**Where:** New Mexico, USA (*Dimetrodon cf. limbatus*).

**When:** Caballo Mountains locality [73], Scholle Member, lower Abo Formation, middle Wolfcampian [74], most likely of Sakmarian age (see Fig. 4).

**Phylogenetic relation:** Based on the apparent greater dimensions of preserved vertebrae, the *Dimetrodon* remnants have been attributed to *Dimetrodon cf. limbatus* instead of *Dimetrodon* cf. *milleri* [74]. Although this is an interesting observation, the presence of a second contemporaneous *Dimetrodon* lineage cannot be confirmed because the material is too poorly preserved to make conclusions. Therefore, these specimens are considered to fall within the same ancestral lineage as *Dimetrodon occidentalis* and *Dimetrodon* cf. *milleri.*

1. ***Dimetrodon cf. natalis***

**Where:** Texas, USA [13].

**When:**

1. Table Branch, locality IIIe of Romer and Price [13], former Putnam Formation, mid-Artiskian age (see Fig. 4).
2. Three Forks of the Little Wichita, locality 5 of Romer [70], locality IVb of Romer and Price [13], former Putnam Formation, mid-Artiskian age (see Fig. 4).

**Phylogenetic relation:** Fragmentary specimens, which are comparable to *D. natalis* [13].

1. ***Lupeosaurus kayi***

**Where:** Archer County, Texas, USA [13, 28, 75].

**When:** USA:

1. Within the former Admiral Formation [75], Nokona Formation, mid-Artiskian age (see Fig. 4).
2. Cottonwood Creek, former Moran Formation [13]. The horizon located ~50 feet below the Sedwick limestone equivalent [76], late Sakmarian-early Artiskian (see Fig. 4).

**Phylogenetic relation:** In the phylogenetic analysis of Mazierski and Reisz [23], *L. kayi* was concluded to be one of the most basal edaphosaurids. See also ref. [7, 77].

1. ***Edaphosaurus “boanerges”***

**Where:** USA:

1. Archer City, Texas, USA. Archer City Bonebed 2 and 3 [78], and Putnam Formation localities referred to by Romer and Price [13] [see also ref. 92].
2. Ohio, USA [79, and references therein].
3. West Virginia, USA [79, and references therein].

**When:**

1. Former Putnam Formation, early Archer City Formation [13, 69], mid-Artiskian age (see Fig. 4).
2. A. Approximately 2 to 3 m below the Waynesburg “A” coal located in the early Washington Formation of the Dunkard Group [79, and references therein; see also ref. 80). According to Lucas [56], it correlates with the early Archer City Formation, which correlates with the mid-Artiskian age (Putnam Formation or older; see Fig. 4).

B. Around the horizon of the Washington “A” coal in the Washington Formation of the Dunkard Group [79, and references therein; see also ref. 80), mid-Artiskian age.

1. Within the lower part of the upper Marietta Sandstone [79, and references therein], which also corresponds with the Waynesburg “A” coal [81] located in the early Washington Formation [80], mid-Artiskian age.

**Phylogenetic relation:** Although the *E. boanerges* species from the Geraldine material has been very well defined (see *E. boanerges* in Amniote Chrone 7 below), the older and fragmentary specimens from the Archer City area (early Archer City Formation) have never been included in a formal phylogenetic analysis. These include the fragmentary specimens from the Putnam Formation referred to by Romer and Price [13] (localities IIIc, IIId, IIIe) and the *Edaphosaurus* cf. *E. boanerges* specimens from Ohio and West Virginia [79, and references therein]. Thus, they are provisionally referred to as *E. “boanerges”* here. Specimens of a contemporaneous age that have been referred to as *Edaphosaurus cf. Edaphsaurus novemexicanus* likely fall within this taxon.

1. ***Euromycter rutenus (*“*Casea*” *rutena)*** [82]

**Where:** Saint-Christophe-Vallon, Valady, France [82].

**When:** M1 (basal) Member of the Grès Rouge (“Red Sandstone”) Group of late Sakmarian age [82].

**Phylogenetic relation:** In the phylogenetic analysis of Brocklehurst and colleagues [8], *E. rutenus* was found to be a member of the family Caseidae.

1. ***Callibrachion gaudryi***

**Where:** Autun Basin, France [36, and references therein].

**When:** Upper Millery Formation [36, and references therein]. The upper Millery Formation is considered to be of early Artiskian age [see the correlation chart of Schneider et al. [68].

**Phylogenetic relation:** *C. gaudryi* has been determined to be a member of the family Caseidae and closely related to the *Datheosaurus* clade [8, 9].

1. ***Vaughnictis smithae (=‘Mycterosaurus’ smithae) [8]***

**Where:** Placerville Locality, Colorado, USA [62].

**When:** *M.* smithae holotype (MCZ 2985) was excavated 100-200 m beneath the top of the upper beds of the Cutler Formation [62]. The upper beds (~1000 feet) of the Cutler Formation are considered to be of Wolfcampian age [63], including the upper Wolfcampian [64]. Therefore, the age of *V. smithae (=‘M. smithae’)* horizon (uppermost Cutler Formation) can be considered to be of upper Wolfcampian (Lenoxian) age.

**Phylogenetic relation:** In the phylogenetic analysis of Brocklehurst et al. [8], *E. rutenus* was found to be a member of the family Caseidae and closely related to the *Eothyris-Oadelops* clade.

1. ***Oedaleops campi***

**Where:** Camp Quarry, New Mexico, USA [83].

**When:** The age of Camp Quarry is considered to be Sakmarian-early Artiskian (Am6 chrone) (see the Methods).

**Phylogenetic relation:** In the phylogenetic analysis of Brocklehurst et al. [8], *O. campi* was found to be a member of the family Caseidae and closely related to the *Eothyris-Vaughnictis* clade.

1. ***Euromycter rutenus (*“*Casea*” *rutena)*** [82]

**Where:** Aveyron, France [82].

**When:** M1 Member of the Grès Rouge Group in Rodez Basin, upper Sakmarian [82].

**Phylogenetic relation:** In the phylogenetic analysis of Brocklehurst et al. [8], *E. rutenus* was found to be a member of the family Caseidae.

1. ***Ascendonanus nestleri***

**Where:** Chemnitz-Hilbersdorf, Germany [9].

**When:** Basal section of Zeisigwald Tuff, Leukersdorf Formation, Sakmarian-Artinskian boundary [9].

**Phylogenetic relation:** *A. nestleri* was determined to be a member of the family Varanopidae and very closely related to *Apsisaurus witteri* forming an exclusive clade [9].

1. ***Apsisaurus witteri***

**Where:** Archer City Bonebed 1 [84].

**When:** Archer City Bonebed 1 [69]; locality IIIf (within the former Putnam Formation) [13], mid-Artiskian age (see Fig. 3).

**Phylogenetic relation:** *A. witteri* was determined to be a member of the family Varanopidae and very closely related to *Apsisaurus nestleri* forming an exclusive clade [9, 85].

1. ***Ruthiromia elcobriensis***

**Where:** Cañon del Cobre, New Mexico [86].

**When:** *R. elcobriensis* was collected from an unknown site in the west wall of Cañon del Cobre [86] rather than from the floor, which strongly suggests a younger age than the fossils collected from the classic sites on the canyon floor [34]. Spielmann and Lucas [55] precisely dated the specimen as late Wolfcampian (early Seymuran; lower part of Arroyo del Agua Formation).

**Phylogenetic relation:** Spielmann and Lucas [55] tentatively placed *R. elcobriensis* in Ophiacodontidae, whereas others placed it in Varanopidae [8, 9] (see also *Aerosaurus wellesi* below).

1. ***Aerosaurus wellesi***

**Where:** Camp Quarry, New Mexico, USA [87].

**When:** The age of Camp Quarry is considered to be Sakmarian-early Artiskian (Am6 chrone) (see the Methods).

**Phylogenetic relation:** *A. wellesi* is a member of the family Varanopidae. Spindler et al. [9] concluded that the genus *Aerosaurus* is paraphyletic with *A. greenleeorum* being more closely related to *Ruthiromia elcobriensis* than to *Aerosaurus wellesi*, which suggests that these taxa should be renamed*.* Because their ages are more proximal to each other than to *A. wellesi*, *R. elcobriensis* and *A. greenleeorum* may be chronospecies. However, other phylogenetic analyses suggest a sister taxon relationship between *A. greenleeorum* and *A. wellesi* [6, 8]. Based on the currently available data regarding the stratigraphical occurrence and the phylogenetic relations of these taxa, the following ancestor-descendant relation is concluded here: *Archaeovenator hamiltonensis* - *Aerosaurus greenleeorum* - *Ruthiromia elcobriensis* - *Aerosaurus wellesi*.

1. ***Baldwinonus trux***

**Where:** Cañon del Cobre, New Mexico, USA [13, 28].

**When:** Romer and Price [13] referred to the specimen collected from Cañon del Cobre by David Baldwin without any other specific information. This means that the age may range from the Gzhelian to the Sakmarian [56].

**Phylogenetic relation:** *B. trux* is considered to be a valid taxon [31, 34 and references therein] and member of the family Ophiacodontidae. It is closely related to *Stereophallodon* [13, 31]. Brinkman and Eberth [31] stated “in most features in which both *Baldwinonus* and *Stereophallodon* are known, *Baldwinonus* is simply a smaller version of *Stereophallodon*”. Due to its fragmentary status (a single fragmentary specimen), it has never been considered in a formal phylogenetic analysis, thus, it is included in the current phylostratigraphic trees with question marks.

1. ***Ophiacodon uniformis***

**Where:** According to ref. [88]:

1. Ohio, USA.
2. Texas, USA.
3. Oklahoma, USA.

**When:** According to ref. [88]:

1. Ohio, probably the earliest Green Formation, which corresponds to the latest Archer City Formation [56], mid-Artiskian age (see Fig. 4).
2. Texas:
3. Geraldine Bonebed. Upper Nokona Formation [30, 78], locality IVa [13], mid-Artiskian age (see Fig. 4).
4. Briar Creek, location 13 of Romer [70], location IVd of Romer and Price [13], Nokona Formation [30], mid-Artiskian age (see Fig. 4).
5. Slippery Creek, south of Dundee, Texas. Locality 20 of Romer [70], locality Vc of Romer and Price [13], former Belle Plains Formation. The Slippery Creek River in the area south of Dundee crosses the Petrolia Formation (and its base, which is the Elm Creek Limestone) and penetrates the upper Nokona Formation as it flows into Lake Kickapoo [30]. Based on the map in Romer [70] and correlation with the Geological Atlas of Hentz and Brown [30], this site falls within the Petrolia Formation, mid to late Artiskian age (see Fig. 4).
6. Godwin Creek, locality 14 of Romer [70], location IVe of Romer and Price [13]. Based on the map in Romer [70] and correlation with the Geological Atlas of Hentz and Brown [30], this site falls within the Nokona Formation. Therefore, it should be of mid-Artiskian age (see Fig. 4).
7. Archer City Bonebed 1 [69]; locality IIIf (within the former Putnam Formation) [13], mid-Artiskian age (see Fig. 4).
8. South of Fulda (Belle Plains), locality 19 of Romer [70]. Based on the map in Romer [70] and correlation with the Geological Atlas of Hentz and Brown [30], this site falls within the Petrolia Formation. Therefore, it should be of mid to late Artiskian age (see Fig. 4).
9. Maybelle, locality VIc of Romer and Price [13], Waggoner Ranch Formation, Kungurian (see Fig. 4).
10. Rattlesnake Canyon, locality IVc of Romer and Price [13], uppermost Nokona Formation, mid-Artiskian age (see Fig. 4).
11. Oklahoma:
12. Perry site 6, bed 16, Oklahoma. The correlation between the site location given in the map in Olson [89] and the modern geological map of Oklahoma [90] suggests that the site is located within the Wellington Formation [see also ref. 88], which correlates with the early Waggoner Ranch Formation [56], latest Artiskian age (see Fig. 4).

**Phylogenetic relation:** *O. uniformis* occurs consistently in the mid-Artiskian, thus the younger specimens probably constitute a distinct species. However, *Ophiacodon* species have never been included in a formal phylogenetic study. Thus, only the oldest representatives of the genus are considered in the current phylostratigraphical trees.

1. ***Ophiacodon retroversus***

**Where:** According to ref. [91]:

1. Oklahoma, USA.
2. Texas, USA.

**When:** According to ref. [91]:

1. Oklahoma, Waurika Site 1, mid-Artiskian age (see Fig. 3 and the Methods).
2. Texas:
3. Cottonwood Creek, former Moran Formation [13]. The horizon was located ~50 feet below the Sedwick limestone equivalent [76], late Sakmarian-early Artiskian (see Fig. 4).
4. Archer City Bonebed 1 [69]; locality IIIf (within the former Putnam Formation) [13], former Putnam Formation, mid-Artiskian age (see Fig. 4).
5. Geraldine Bonebed. Upper Nokona Formation [30, 78], mid-Artiskian age (see Fig. 4).
6. Rattlesnake Canyon, locality IVc of Romer and Price [13], uppermost Nokona Formation, mid-Artiskian age (see Fig. 4).
7. Three Forks of the Little Wichita, locality 5 of Romer [70], locality IVb of Romer and Price [13], former Putnam Formation, mid-Artiskian age (see Fig. 4).
8. Middle Fork of the Little Wichita, locality 10 of Romer [70], locality Vg of Romer and Price [13], former Admiral Formation, mid-Artiskian age (see Fig. 4).
9. Mount Barry, locality 12 of Romer [70], locality IVf of Romer and Price [13]. Based on the map in Romer [70] and correlation with the Geological Atlas of Hentz and Brown [30], this site falls within the Petrolia Formation, mid to late Artiskian age (see Fig. 4).
10. Godwin Creek, locality 14 of Romer [70]. Based on the map in Romer [70] and correlation with the Geological Atlas of Hentz and Brown [30], this site falls within the Nokona Formation. Therefore, it should be of mid-Artiskian age (see Fig. 4).
11. Slippery Creek, south of Dundee, Texas. Locality 20 of Romer [70], locality Vc of Romer and Price [13], former Belle Plains Formation. The Slippery Creek River in the area south of Dundee crosses the Petrolia Formation (and its base, which is the Elm Creek Limestone) and penetrates the upper Nokona Formation as it flows into Lake Kickapoo [30]. Based on the map in Romer [70] and correlation with the Geological Atlas of Hentz and Brown [30], this site falls within the Petrolia Formation, mid to late Artiskian age (see Fig. 4).
12. Big Wichita, locality 23 of Romer [70], locality Ve of Romer and Price [13]. Based on the map in Romer [70] and correlation with the Geological Atlas of Hentz and Brown [30], the fossils of this site are likely from the Petrolia Formation. Therefore, they should be of mid to late Artiskian age (see Fig. 4).
13. Tit Mountain, locality 21 of Romer [70], locality Vd of Romer and Price [13]. Based on the map in Romer [70] and correlation with the Geological Atlas of Hentz and Brown [30], this site falls within the Petrolia Formation. Therefore, it should be of mid to late Artiskian age (see Fig. 4).
14. South of Fulda (Belle Plains), locality 19 of Romer [70]. Based on the map in Romer [70] and correlation with the Geological Atlas of Hentz and Brown [30], this site falls within the Petrolia Formation. Therefore, it should be of late Artiskian age (see Fig. 4).

**Phylogenetic relation:** *Ophiacodon* species have never been included in a formal phylogenetic study. Thus, only the oldest representatives of the genus are considered in the current phylostratigraphical trees.

1. ***Edaphosaurus? credneri***

**Where:** Döhlen Basin, Dresden, Germany [61, 65].

**When:** It was found within the lacustrine Limestone Member in the uppermost Niederhäslich Formation [65]. Recently reported radioisotopic dates from the Döhlen Basin [51] propose an early Artiskian age (~289 Ma).

**Phylogenetic relation:** *Edaphosaurus credneri* has never been included in a formal phylogenetic study. Thus, it is not further considered here.

**Amniote chrone 7 (Am7)**

1. ***Eothyris parkeyi***

**Where:** Texas, USA [28].

**When:** Romer [28] stated that the fossils were found approximately one mile west of the former Woodrum ranch house, south of Dundee, Archer County, and within the horizon of the former Belle Plains Formation. However, Romer and Price [13] noted that the fossils were from the Vd locality [Tit Mountain, locality 21 of Romer [70]]. The initial locality described appears to be more accurate, although the exact location of the Woodrum ranch house is unknown. However, elsewhere, recovery of a *Diadectes* specimen has been described as “5 miles south of Dundee, near Woodrum ranch house”. See <http://www.paleofile.com/Anapsidalist/Diadectes.asp>

In this case, correlation with the Geological Atlas of Hentz and Brown [30] attributes a lower Petrolia Formation level for the *Eothyris parkeyi* specimen, which is of mid-Artiskian age (see Fig. 4).

**Phylogenetic relation:** Although of a relatively younger age, *Eothyris* is considered to be a primitive and basal member of Caseasauria (e.g. ref. [7, 8].

1. ***Ruthenosaurus russellorum***

**Where:** Saint-Christophe-Vallon, Valady, France [82].

**When:** Upper part of the red pelitic beds of the M2 Member, of the Grès Rouge of the Rodez Basin [82]. This sequence has five members extending from the upper Sakmarian to the lower Lopingian [82]. Therefore, a precise age estimate is not possible.

**Phylogenetic relation:** *R. russellorum* has never been included in a formal phylogenetic study. Because of this and the unestablished age of this taxon, it is not further considered here.

1. ***Edaphosaurus boanerges***

**Where:** USA:

1. Texas, USA [92].
2. Oklahoma, USA [89, 92].
3. Pittsburgh-West Virginia region, USA [93].

**When:**

1. Texas:
2. Coprolite Bonebed, lowermost Nocona Formation [69], mid-Artiskian age (see Fig. 4).
3. Loftin Bonebed, Nokona Formation [69], mid-Artiskian age (see Fig. 4).
4. Geraldine Bonebed. Upper Nokona Formation [30, 78], mid-Artiskian age (see Fig. 4).
5. Rattlesnake Canyon, locality IVc of Romer and Price [13], uppermost Nokona Formation, mid-Artiskian age (see Fig. 4).
6. Archer City Bonebed 2 & 3 [69], mid-Artiskian age (see Fig. 4).
7. Oklahoma:
8. Waurika Site 1, mid-Artiskian age (see Fig. 3 and the Methods).
9. Orlando site, Wellington Formation (*Edaphosaurus cf* *E. boanerges*) [89, 90], correlated to the lower Waggoner Ranch Formation [56], late Artiskian age (see Fig. 4).
10. McCann Quarry site, Wellington/Garber Formation boundary [89, 90], correlated to the lower Waggoner Ranch Formation [56], Kungurian age (see Fig. 4).
11. Ohio River drainage (*Edaphosaurus cf* *E. boanerges*) [93], lowermost Greene formation, probably equivalent to the latest Archer City Formation [56], mid-Artiskian age (see Fig. 4).

**Phylogenetic relation:** *E. boanerges* has been described well on the basis of rich and well-preserved skeletons from the Geraldine Bonebed [94] which are of mid-Artiskian age. In the phylogenetic analysis by Mazierski and Reisz [23], *E. boanerges* was concluded to be a basal edaphosaurid related to *E. novomexicanus* [see also ref. 7]**.** All contemporaneous specimens excavated from mid-Artiskian horizons (see above) can be attributed to this species. Older species should be attributed to a new species (see *E. “boanerges”* in Amniote chrone 6). The younger specimens of *Edaphosaurus cf* *E. boanerges* specimens from Orlando Site and McCann Quarry can be attributed to *E. crusiger* as proposed by ref. [89].

1. ***Ctenorhachis jacksoni***

**Where:** South Fulda, Texas, USA [95].

**When:** Petrolia Formation, mid-to-late Artiskian age (see Fig. 4).

**Phylogenetic relation:** *C. jacksoni* seems to be a member of Sphenacodontia and is probably a stem of the sphenacodontids and/or the therapsids [7, 8].

1. ***Secodontosaurus obtusidens***

**Where:** Texas, USA [13].

**When:**

1. Rattlesnake Canyon, locality IVc of Romer and Price [13], uppermost Nokona Formation, mid-Artiskian age (see Fig. 4).
2. Briar Creek, location 13 of Romer [70], location IVd of Romer and Price [13], Nokona Formation [30], mid-Artiskian age (see Fig. 4).
3. Godwin Creek, locality 14 of Romer [70], location IVe of Romer and Price [13]. Based on the map in Romer [70] and correlation with the Geological Atlas of Hentz and Brown [30], this site falls within the Nokona Formation. Therefore, it should be of mid-Artiskian age (see Fig. 4).
4. Mount Barry, locality 12 of Romer [70], locality IVf of Romer and Price [13]. Based on the map in Romer [70] and correlation with the Geological Atlas of Hentz and Brown [30], this site falls within the Petrolia Formation, mid-to-late Artiskian age (see Fig. 4).
5. Tit Mountain, locality 21 of Romer [70], locality Vd of Romer and Price [13]. Based on the map in Romer [70] and correlation with the Geological Atlas of Hentz and Brown [30], this site falls within the Petrolia Formation. Therefore, it should be of mid-to-late Artiskian age (see Fig. 4).
6. Beaver Creek, locality 24 of Romer [70], locality Vf of Romer and Price [13], upper Petrolia Formation, late Artiskian age (see Fig. 4).

**Phylogenetic relation:** *Secodontosaurus obtusidens* has been determined to be in the family Sphenacodontidae [8, 59].

1. ***Dimetrodon limbatus***

**Where:** USA:

- 1. Oklahoma, USA [89].
  2. Texas, USA [13, 96].

**When:**

1. Waurika Site 1, mid-Artiskian age (see Fig. 3 and the Methods).
2. Texas:
3. Shell Point, locality 6 of Romer [70], locality IVh of Romer and Price [13], northwest Archer City, south of the Little Wichita River. Based on the Geological Atlas of Hentz and Brown [30], this site falls within the Nokona Formation, mid-Artiskian age (see Fig. 4).
4. Mount Barry, locality 12 of Romer [70], locality IVf of Romer and Price [13]. Based on the map in Romer [70] and correlation with the Geological Atlas of Hentz and Brown [30], this site falls within the Petrolia Formation, mid-to-late Artiskian age (see Fig. 4).
5. Briar Creek, location 13 of Romer [70], location IVd of Romer and Price [13], Nokona Formation [30], mid-Artiskian age (see Fig. 4).
6. Godwin Creek, locality 14 of Romer [70], location IVe of Romer and Price [13]. Based on the map in Romer [70] and correlation with the Geological Atlas of Hentz and Brown [30], this site falls within the Nokona Formation. Therefore, it should be of mid-Artiskian age (see Fig. 4).
7. Daggett Creek. Location 18 of Romer [70], location Va of Romer and Price [13], former Belle Plain Formation, latest Artiskian age (see Fig. 4).
8. Locality Vb of Romer and Price [13], former Belle Plain Formation, latest Artiskian age (see Fig. 4).
9. Rattlesnake Canyon Bonebed 2, uppermost Nokona Formation [69], mid-Artiskian age (see Fig. 4).
10. Numerous other specimens described in Romer and Price [13] fall within the IV (former Admiral Formation) and V (former Belle Plains Formation) areas of the mid-to-late Artiskian age (see Fig. 4).

**Phylogenetic relation:** Romer and Price [13] noted that the *D. limbatus* and *D. milleri* specimens were very similar *D. limbatus* from Briar Creek has been determined to be a species distinct from *D. milleri and D. grandis* based on the different tooth morphologies [58]. See also *D. booneorum* below.

1. ***Dimetrodon booneorum***

**Where:** Texas, USA [13, 97].

**When:**

1. Rattlesnake Canyon, locality IVc of Romer and Price [13], uppermost Nokona Formation, mid-Artiskian age (see Fig. 4).
2. Briar Creek, location 13 of Romer [70], location IVd of Romer and Price [13], Nokona Formation [30], mid-Artiskian age (see Fig. 4).
3. Godwin Creek, locality 14 of Romer [70], location IVe of Romer and Price [13]. Based on the map in Romer [70] and correlation with the Geological Atlas of Hentz and Brown [30], this site falls within the Nokona Formation. Therefore, it should be of mid-Artiskian age (see Fig. 4).
4. Locality IV-V of Romer and Price [13]. It is not a definable collecting area and is believed to fall within the Nocona and Petrolia Formations, mid-to-late Artiskian age (see Fig. 4).
5. Locality Vb of Romer and Price [13], former Belle Plain Formation, mid to late Artiskian age (see Fig. 4).
6. Slippery Creek, south of Dundee, Texas. Locality 20 of Romer [70], locality Vc of Romer and Price [13], former Belle Plains Formation. The Slippery Creek River in the area south of Dundee crosses the Petrolia Formation (and its base, which is the Elm Creek Limestone) and penetrates the upper Nokona Formation as it flows into Lake Kickapoo [30]. Based on the map in Romer [70] and correlation with the Geological Atlas of Hentz and Brown [30], this site falls within the Petrolia Formation, mid to late Artiskian age (see Fig. 4).

**Phylogenetic relation:** The three sympatric and contemporaneous species of the genus *Dimetrodon*, *D. natalis*, *D. booneorum*, and *D. limbatus* have been previously considered an ontogenetic series with each species representing a small, an intermediate, and a large species, respectively. However, a more recent histologic study concluded that the small species *D. natalis*, and a different large species were present in the examined material from Briar Creek, whereas the status of *D. booneorum* and *D. limbatus* remained unresolved [98]. Here, *D. booneorum* and *D. limbatus* are considered one species.

1. ***Dimetrodon natalis***

**Where:** Various localities of Texas, USA (see below) [13, 99].

**When:**

1. Geraldine Bonebed. Upper Nokona Formation [30, 78], mid-Artiskian (see Fig. 4).
2. Rattlesnake Canyon, locality IVc of Romer and Price [13], uppermost Nokona Formation, mid-Artiskian age (see Fig. 4).
3. Godwin Creek, locality 14 of Romer [70], location IVe of Romer and Price [13]. Based on the map in Romer [70] and correlation with the Geological Atlas of Hentz and Brown [30], this site falls within the Nokona Formation. Therefore, it should be of mid-Artiskian age (see Fig. 4).
4. Mount Barry, locality 12 of Romer [70], locality IVf of Romer and Price [13]. Based on the map in Romer [70] and correlation with the Geological Atlas of Hentz and Brown [30], this site falls within the Petrolia Formation, mid-to-late Artiskian age (see Fig. 4).
5. Locality Vb of Romer and Price [13], former Belle Plain Formation, mid to late Artiskian age (see Fig. 4).
6. Slippery Creek, south of Dundee, Texas. Locality 20 of Romer [70], locality Vc of Romer and Price [13], former Belle Plains Formation. The Slippery Creek River in the area south of Dundee crosses the Petrolia Formation (and its base, which is the Elm Creek Limestone) and penetrates the upper Nokona Formation as it flows into Lake Kickapoo [30]. Based on the map in Romer [70] and correlation with the Geological Atlas of Hentz and Brown [30], this site falls within the Petrolia Formation, mid to late Artiskian age (see Fig. 4).
7. Tit Mountain, locality 21 of Romer [70], locality Vd of Romer and Price [13]. Based on the map in Romer [70] and correlation with the Geological Atlas of Hentz and Brown [30], this site falls within the Petrolia Formation. Therefore, it should be of mid-to-late Artiskian age (see Fig. 4).

**Phylogenetic relation:** See *D. booneorum* above.

1. ***Ctenospondylus casei***

**Where:** USA:

1. Archer County, Texas [13]
2. San Juan County, Utah [100]

**When:**

1. Slippery Creek, south of Dundee, Texas. Locality 20 of Romer [70], locality Vc of Romer and Price [13], former Belle Plains Formation. The Slippery Creek River in the area south of Dundee crosses the Petrolia Formation (and its base, which is the Elm Creek Limestone) and penetrates the upper Nokona Formation as it flows into Lake Kickapoo [30]. Based on the map in Romer [70] and correlation with the Geological Atlas of Hentz and Brown [30], this site falls within the Petrolia Formation, mid to late Artiskian age (see Fig. 4).
2. Organ Rock Shale Member (Cutler Formation), Leonardian/Artinskian age [101] (see fig. 4)

**Phylogenetic relation:** A sphenacodont more closely related to the *Sphenacodon* [58, 59], *Dimetrodon* and *Sphenacodon* [44, 52] and the *Cryptovenator* [44].

1. ***Ctenospondylus ninevehensis***

**Where:** Monroe County, Ohio, USA [102].

**When:** Niniveh Limestone Member, deposited at the lower third of the Greene Formation [103], thus, corresponding to the middle of Petrolia Formation [56], that is, late Artiskian age (Am7) (see Fig. 4).

**Phylogenetic relation:** *Ctenospondylus ninevehensis,* although contemporaneous with *Ctenospondylus casei,* displays more primitive features that make it to look like an ancestor of *C. casei* [102].

**Amniote chrone 8 (Am8)**

1. ***Dimetrodon loomisi***

**Where:** USA:

1. Texas, USA [13].
2. Oklahoma, USA [104].

**When:**

1. Texas:
2. Coffee Creek, locality 34 of Romer [70], locality VIIa of Romer and Price [13], Arroyo Formation, mid-Kungurian age (see Fig. 4).
3. Craddock Bonebed, Brush Creek, locality 38 of Romer [70], locality VIIb of Romer and Price [13]. Arroyo Formation, Kungurian age (see Fig. 4).
4. Hog Creek, locality 39 of Romer [70], locality VIId of Romer and Price [13]. Arroyo Formation, mid-Kungurian age.
5. Locality VIIg of Romer and Price [13]. Arroyo Formation, Kungurian age.

Poney Creek, locality VIIh of Romer and Price [13]. Arroyo Formation, Kungurian age.

1. Oklahoma, South Grandfield, Hennessey Formation, Kungurian age [104].

**Phylogenetic relation:** *D. loomisi* is represented by a nearly perfect skeleton and is similar to its larger contemporary *D. gigashomogenes* in most respects, but is distinguished by its straight spines and by an unusually large number of

lower jaw teeth [13]. It can be considered a valid taxon, which is sufficiently represented in the fossil record.

1. ***Dimetrodon macrospondylus***

**Where:** USA [13]:

1. Texas.
2. Oklahoma.

**When:**

1. Texas, Tit Mountain, locality 21 of Romer [70], locality Vd of Romer and Price [13]. Based on the map in Romer [70] and correlation with the Geological Atlas of Hentz and Brown [30], this site falls within the Petrolia Formation. Therefore, it should be of mid-to-late Artiskian age (see Fig. 4).
2. Oklahoma, Deep Red Run, Clyde or Garber Formation, Kungurian age (Fig. 4).

**Phylogenetic relation:** *D. macrospondylus* is a poorly known species, is about the size of *D. loomisi*, but differs in that it has a normal tooth count [13]. However, the lower jaw collected with the *D. macrospondylus* fossil may not belong to this species [13]. Thus, *D. macrospondylus* may not be a valid taxon and is not further considered here.

1. ***Dimetrodon gigashomogenes***

**Where:** Various localities of Texas, USA (see below) [13].

**When:**

1. Coffee Creek, locality 34 of Romer [70], locality VIIa of Romer and Price [13], Arroyo Formation, Kungurian age.
2. Craddock Bonebed, Brush Creek, locality 38 of Romer [70], locality VIIb of Romer and Price [13]. Arroyo Formation, Kungurian age.
3. Indian Creek (Cacops Bonebed), locality VIIc of Romer and Price [13]. Arroyo Formation, mid-Kungurian age (see Fig. 4).
4. Poney Creek, locality VIIh of Romer and Price [13]. Arroyo Formation, Kungurian age.
5. Multiple other localities of Kungurian age [105].

**Phylogenetic relation:** *D. gigashomogenes* is very similar to *D. loomisi* and their morphological differences could be sexual dimorphism [13]; this interpretation is adopted here.

1. ***Dimetrodon dollovianus***

**Where:** USA [13]:

1. Texas.
2. Oklahoma.

**When:**

1. Texas:

1. Locations VIb, VIc and Vid of Romer and Price [13], Clyde Formation, Kungurian.

2. Texas, Tit Mountain, locality 21 of Romer [70], locality Vd of Romer and Price [13]. Based on the map in Romer [70] and correlation with the Geological Atlas of Hentz and Brown [30], this site falls within the Petrolia Formation. Therefore, it should be of mid-to-late Artiskian age (see Fig. 4).

1. Oklahoma, Deep Red Run, Clyde or Garber Formation, Kungurian age (Fig. 4).

**Phylogenetic relation:** According to Romer and Price [13], *D. dollovianus* is most likely identical to *D. gigashomogenes*; this interpretation is adopted here.

1. ***Dimetrodon angelensis***

**Where:** Texas, USA [106].

**When:** MacFayden Ranch, Knox County, upper San Angelo Formation [106].

**Phylogenetic relation:** According to Olson [106], the morphology of *D. angelensis* is very similar to *D. gigashomogenes*, but the skull of *D. gigashomogenes* has not been characterized enough for a proper comparison. Therefore, *D. angelensis* is most likely not a valid taxon and may be identical to *D. gigashomogenes.* Thus, it is not further considered here.

1. ***Dimetrodon teutonis***

**Where:** Thuringian Forest Basin, central Germany [72, 107].

**When:** Bromacker quarry locality, Tambach Formation, early Kungurian (see the correlation chart of ref. [68]).

**Phylogenetic relation:** Very closely related to *D. natalis* [72, 107].

1. ***Dimetrodon grandis***

**Where:** Various localities of Texas, USA (see below) [13].

**When:**

1. Coffee Creek, locality 34 of Romer [70], locality VIIa of Romer and Price [13], Arroyo Formation, Kungurian age.
2. Craddock Bonebed, Brush Creek, locality 38 of Romer [70], locality VIIb of Romer and Price [13]. Arroyo Formation, Kungurian age.
3. Indian Creek (Cacops Bonebed), locality VIIc of Romer and Price [13]. Arroyo Formation, mid-Kungurian age (see Fig. 4).
4. Hog Creek, locality 39 of Romer [70], locality VIId of Romer and Price [13]. Arroyo Formation, Kungurian age.
5. Crooked Creek. Locality VIIf of Romer and Price [13]. Arroyo Formation, Kungurian age.
6. Locality VIIg of Romer and Price [13]. Arroyo Formation, Kungurian age.

**Phylogenetic relation:** Romer and Price [13] noted that *D. grandis* differs little from *D. limbatus* and may have descended from *D. limbatus,* the earlier species. *D. grandis* has also been analysed in thorough phylogenetic studies and was found to related to *D. limbatus* and *Bathygnathus borealis* [58, 59, 108].

1. ***Dimetrodon (=Bathygnathus) borealis***

**Where:** Prince Edward Island, Canada [59, and references therein].

**When:** French River District, Orby Head Formation, mid-to-late Artiskian [59].

**Phylogenetic relation:** *B. borealis* was found to be closely related to the genus *Dimetrodon* and to be the sister taxon to *Dimetrodon grandis* [59]*.*

1. ***Tetraceratops insignis***

**Where:** Big Wichita River, Baylor County, Texas, USA [13].

**When:** Arroyo Formation [13], mid-Kungurian age (see Fig. 4).

**Phylogenetic relation:** *T. insignis* was previously considered a therapsid [50, and references therein]. However, a recent thorough phylogenetic study placed *T. insignis* close to *Pantelosaurus saxonicus* [50].

1. ***Dimetrodon kempae***

**Where:** Various localities of Texas, USA (see below) [13].

**When:**

1. Coffee Creek, locality 34 of Romer [70], locality VIIa of Romer and Price [13], Arroyo Formation, Kungurian age (see Fig. 4).
2. Craddock Bonebed, Brush Creek, locality 38 of Romer [70], locality VIIb of Romer and Price [13]. Arroyo Formation, Kungurian age (see Fig. 4).
3. Poney Creek, locality VIIh of Romer and Price [13]. Arroyo Formation, Kungurian age (see Fig. 4).

**Phylogenetic relation:** *D. kempae* is a very poorly known and questionable species of *Dimetrodon* [13]. Reisz [109] considered it to be a Sphenacodontidae *incertae sedis*. Thus, here it is provisionally considered a sphenacodontid outside of the genus *Dimetrodon*.

1. ***Secodontosaurus willisoni***

**Where:** Various localities of Texas, USA (see below) [13].

**When:**

1. Coffee Creek, locality 34 of Romer [70], locality VIIa of Romer and Price [13], Arroyo Formation, Kungurian age (see Fig. 4).
2. Craddock Bonebed, Brush Creek, locality 38 of Romer [70], locality VIIb of Romer and Price [13]. Arroyo Formation, Kungurian age (see Fig. 4).

**Phylogenetic relation:** *S. willisoni* is a species with a stratigraphical age younger than *S. obtusidens*, thus it can be considered an advanced stage of this species.

1. ***Dimetrodon cf. limbatus***

**Where:** USA [96]:

1. Oklahoma.
2. Texas.

**When:**

1. Perry Site 6, bed 16, Oklahoma. Correlation of the site location given in the Olson [89] map and the modern geological map of Oklahoma [90] suggests that the site is located within the Wellington Formation [see also ref. 88], which correlates with the early Waggoner Ranch Formation [56], late Artiskian-early Kungurian age (see Fig. 4).
2. Craddock Bonebed, locality 38 of Romer [70], locality VIIb of Romer and Price [13]. Arroyo Formation, Kungurian age.

**Phylogenetic relation:** These isolated specimens have never been analysed in detail and they were recovered from younger localities than the well-studied *D. limbatus* specimens. Therefore, they are likely not related to *D. limbatus.*

1. ***Glaucosaurus megalops***

**Where:** Texas, USA [13].

**When:** Mitchell Creek, northeast of Maybelle, Texas, USA. Locality 29 of Romer [70], locality VIa of Romer and Price [13]. Based on the map in Romer [70] and correlation with the Geological Atlas of Hentz and Brown [30], this site falls within the Waggoner Ranch Formation. Therefore, it should be of early Kungurian age (see Fig. 4).

**Phylogenetic relation:** Although of relatively younger age, *Glaucosaurus* is considered to be a primitive and basal member of the Edaphosauridae [7, 8, 23].

1. ***Edaphosaurus cruciger***

**Where:** USA [13, 110]:

1. Texas.
2. Oklahoma.

**When:**

1. Texas:
2. Big Wichita, locality 23 of Romer [70], locality Ve of Romer and Price [13]. Based on the map in Romer [70] and correlation with the Geological Atlas of Hentz and Brown [30], the fossils of this site are likely from the Petrolia Formation. Therefore, they should be of mid-to-late Artiskian age (see Fig. 4).
3. Beaver Creek, locality 24 of Romer [70], locality Vf of Romer and Price [13], upper Petrolia Formation, late Artiskian age (see Fig. 4).
4. Maybelle, locality VIc [13], Waggoner Ranch Formation, Kungurian (see Fig. 4).
5. Baylor County, Lueders Formation, Maybelle Limestone Member [110], mid-Kungurian age (see Fig. 4).
6. Oklahoma, Deep Red Run, Clyde or Garber Formation [110], Kungurian age.

**Phylogenetic relation:** *E. crusiger* is a valid taxon, which is considered a sister taxon to *E. pogonias* in the family Edaphosauridae [23, 35].

1. ***Edaphosaurus pogonias***

**Where:** Various localities of Texas, USA (see below) [13].

**When:**

1. Coffee Creek, locality 34 of Romer [70], locality VIIa of Romer and Price [13], Arroyo Formation, Kungurian age.
2. Craddock Bonebed, Brush Creek, locality 38 of Romer [70], locality VIIb of Romer and Price [13]. Arroyo Formation, Kungurian age.
3. Hog Creek, locality 39 of Romer [70], locality VIId of Romer and Price [13]. Arroyo Formation, Kungurian age.

**Phylogenetic relation:** *E. pogonias* is a valid taxon, which is considered as a sister taxon to *E. cruciger* in the family Edaphosauridae [23, 35].

1. ***Oromycter dolesorum***

**Where:** Dolese Brothers limestone quarry, Oklahoma, USA [111].

**When:** Horizon stratigraphically equivalent to the lowermost Clear Fork Group [111], mid-Kungurian age (see Fig. 4).

**Phylogenetic relation:** *O. dolesorum* is a valid taxon and member of the family Caseidae [8, 35].

1. ***Trichasaurus texensis***

**Where:** Various localities of Texas, USA (see below) [13].

**When:**

1. Craddock Bonebed, Brush Creek, locality 38 of Romer [70], locality VIIb of Romer and Price [13]. Arroyo Formation, Kungurian age.
2. Indian Creek (Cacops Bonebed), locality VIIc of Romer and Price [13]. Arroyo Formation, mid-Kungurian age (see Fig. 4).

**Phylogenetic relation:** *T. texensis* is a valid taxon and member of the family Caseidae [6, 8].

1. ***Angelosaurus romeri***

**Where:** Site KF1, Kingfisher County, Oklahoma, USA [112].

**When:** Chickasha Tongue, Chickasha Formation in the middle of the Flowerpot Formation [112], latest Kungurian age (see Fig. 4).

**Phylogenetic relation:** *A. romeri* is a valid taxon and young member of the family Caseidae [6, 8].

1. ***Angelosaurus dolani***

**Where:** Sublocality KN, Little Croton Creek, Knox County, Texas, USA [113].

**When:** Red shale facies of middle part of San Angelo Formation [113], latest Kungurian age (see Fig. 4).

**Phylogenetic relation:** Olson and Beerbower [113] described *A. dolani* as a young member of the family Caseidae, however, it has never been analysed in a formal phylogenetic study. Thus, it is not further considered here.

1. ***Angelosaurus greeni***

**Where:** Sublocality KR, Alexander Ranch, Knox County, USA [106].

**When:** Flowerpot Formation, ~15 feet above the contact with the San Angelo Formation [106], latest Kungurian age (see Fig. 4).

**Phylogenetic relation:** Olson [106] described *A. greeni* as a young member of the family Caseidae, however, it has never been analysed in a formal phylogenetic study. Thus, it is not further considered here.

1. ***Casea halselli***

**Where:** Locality FC, Halsell ranch, Foard County, Texas, USA [114].

**When:** Middle part of Choza Formation [114], latest Kungurian age (see Fig. 4).

**Phylogenetic relation:** *C. halselli* is a valid taxon and a young member of the family Caseidae [8].

1. ***Casea broilii***

**Where:** Texas, USA [13].

**When:** Indian Creek (Cacops Bonebed), locality 35 of Romer [70], locality VIIc of Romer and Price [13]. Arroyo Formation, mid-Kungurian age (see Fig. 4).

**Phylogenetic relation:** *C. broilii* is a valid taxon and a young member of the family Caseidae [6, 8].

1. ***Casea nicholsi***

**Where:** Two localities of Texas, USA (see below) [115].

**When:**

1. Taylor County Site 7, Clear Fork Group, mid-Kungurian age (see Fig. 4).
2. Locality KC, upper Vale Formation (see Fig. 4).

**Phylogenetic relation:** *C. broilii* is considered a young member of the family Caseidae, but it has never been analysed in a formal phylogenetic study. Thus, it is not further considered here.

1. ***Cotylorhynchus hancocki***

**Where:** Texas, USA (116).

**When:** It has been recovered from many sites in the San Angelo Formation and from a site in the lower part of the Flowerpot Formation [106, 113, 115], latest Kungurian age (see Fig. 4).

**Phylogenetic relation:** *C. hancocki* is a valid taxon and a young member of the family Caseidae [6, 8].

1. ***Cotylorhynchus bransoni***

**Where:** Texas, USA [117].

**When:** It has been recovered from many sites in the Chickasha Tongue (Chickasha Formation) [117] which deposited in and in the middle of the Flowerpot Formation [106], latest Kungurian age (see Fig. 4).

**Phylogenetic relation:** *C. bransoni* is a valid taxon and a young member of the family Caseidae [6, 8].

1. ***Cotylorhynchus romeri***

**Where:** Near Navina, Logan County, Oklahoma, USA [13, and references therein].

**When:** Hennessey Formation, Kungurian age (see Fig. 4).

**Phylogenetic relation:** *C. romeri* is a valid taxon and a young member of the family Caseidae [6, 8].

1. ***Varanops brevirostris***

**Where:** Texas, USA [13, 118].

**When:**

1. Indian Creek (Cacops Bonebed), locality VIIc of Romer and Price [13]. Arroyo Formation, mid-Kungurian age (see Fig. 4).
2. Southwest Abilene, Taylor County, Arroyo Formation, mid-Kungurian age (see Fig. 4).

**Phylogenetic relation:** *V. brevirostris* is a valid taxon and a young member of the family Varanopidae. *V. brevirostris* is a sister taxon to *T. unguifalcatus* within a clade that forms the sister clade to the terminal dichotomy *Varanodon*+*Watongia* [33, 119] or to *T. unguifalcatus* outside of the sister taxon relation [8, 9].

1. ***Varanops “brevirostris”* (“Richards Spur *Varanops*”)**

**Where:** Dolese Brothers limestone quarry, Comanche County, Oklahoma, USA [120].

**When:** Horizon stratigraphically equivalent to the lowermost Clear Fork Group [111], mid-Kungurian age (see Fig. 4).

**Phylogenetic relation:** Although the Richards Spur *Varanops cf. V. brevirostris* was previously thought to be identical to *V. brevirostris* [120], it has recently been shown to be a separate species and a sister taxon to *V. brevirostris* within a clade that forms the sister clade of the terminal dichotomy *Varanodon*+*Watongia* [9].

1. ***Varanodon agilis***

**Where:** Hitchcock, Blaine County, Oklahoma, USA [121].

**When:** Chickasha Formation, equivalent to the middle Flowerpot Formation [121], latest Kungurian age (see Fig. 4).

**Phylogenetic relation:** *V. agilis* is a valid taxon and a young member of the family Varanopidae, which is a sister taxon to *W. meieri* within a clade that forms the sister clade to *V. brevirostris* [8, 9].

1. ***Watongia meieri***

**Where:** Fossil site BC7, Blaine County, Oklahoma, USA [122].

**When:** Chickasha Formation, equivalent to the middle Flowerpot Formation [121], latest Kungurian age (see Fig. 4).

**Phylogenetic relation:** *W. meieri* is a valid taxon, a young member of the family Varanopidae, and a sister taxon to *V. agilis* within a clade that forms the sister clade to *V. brevirostris* [8, 9].

1. ***Tambacarnifex unguifalcatus***

**Where:** Thuringian Forest Basin, central Germany [33].

**When:** Bromacker quarry locality, Tambach Formation, early Kungurian (see the correlation chart of ref. [68]).

**Phylogenetic relation:** *T. unguifalcatus* is a valid taxon, is a young member of the family Varanopidae [33], is close to *M. longiceps*, and is outside of the clade of *W. meieri, V. agilis*, and *V. brevirostris* [8, 9].

1. ***Mycterosaurus longiceps***

**Where:** Texas, USA [13].

**When:** Mitchell Creek, northeast of Maybelle, Texas, USA. Locality 29 of Romer [70], locality VIa of Romer and Price [13]. Based on the map in Romer [70] and correlation with the Geological Atlas of Hentz and Brown [30], this site falls within the Waggoner Ranch Formation. Therefore, it should be of early Kungurian age (see Fig. 4).

**Phylogenetic relation:** *M. longiceps* is a valid taxon, is a young member of the family Varanopidae, is close to *T. unguifalcatus*, and is outside of the clade of *W. meieri, V. agilis* and *V. brevirostris* [6, 9, 85].

1. ***Varanosaurus wichitaensis***

**Where:** Texas, USA (see below) [13].

**When:**

1. Slippery Creek, south of Dundee, Texas. Locality 20 of Romer [70], locality Vc of Romer and Price [13], former Belle Plains Formation. The Slippery Creek River in the area south of Dundee crosses the Petrolia Formation (and its base, which is the Elm Creek Limestone) and penetrates the upper Nokona Formation as it flows into Lake Kickapoo [30]. Based on the map in Romer [70] and correlation with the Geological Atlas of Hentz and Brown [30], this site falls within the Petrolia Formation, mid to late Artiskian age (see Fig. 4).
2. Mitchell Creek, northeast of Maybelle, Texas, USA. Locality 29 of Romer [70], locality VIa of Romer and Price [13]. Based on the map of Romer [70] and correlation with the Geological Atlas of Hentz and Brown [30], this site falls within the Waggoner Ranch Formation. Therefore, it should be of early Kungurian age (see Fig. 4).

**Phylogenetic relation:** *V. wichitaensis* is a member of the Ophiacodontidae outside of the *Ophiacodon* clade [8].

1. ***Varanosaurus acutirostris***

**Where:** Various localities of Texas, USA (see below) [13].

**When:**

1. Coffee Creek, locality 34 of Romer [70], locality VIIa of Romer and Price [13], Arroyo Formation, mid-Kungurian age (see Fig. 4).
2. Craddock Bonebed, Brush Creek, locality 38 of Romer [70], locality VIIb of Romer and Price [13]. Arroyo Formation, Kungurian age (see Fig. 4).

**Phylogenetic relation:** *Varanosaurus* is a member of the Ophiacodontidae outside of the *Ophiacodon* clade [6, 7, 8].

**Amniote chrone 9 (Am9)**

1. ***Phreatoplasma aenigmaticum***

**Where:** Santagulov Mine, Bashkortostan Province, Russian Federation [123, and references therein].

**When:** Golyusherma Subassemblage, early Kazanian age, equivalent to the Roadian Stage [123, and references therein].

**Phylogenetic relation:** Although poorly preserved, *P. aenigmaticum* is considered to be a member of the family Caseidae [123].

1. ***Ennatosaurus tecton***

**Where:** Moroznitsa and Nyisagora localities, Northwestern Russian Federation [124, and references therein].

**When:** Earliest Tatarian [124, and references therein], earliest Severodvinian (see Fig. 2).

**Phylogenetic relation:** A phylogenetic analysis placed *Ennatosaurus tecton* as the sister taxon to the clade of the North American caseids *Angelosaurus dolani* and *Cotylorhynchus romeri* [124].

**126**

1. ***Mesenosaurus romeri***

**Where:** Northwestern Russian Federation [125].

**When:** Mezen Assemblage, lower Tatarian, Urzhumian [125], Wordian (see Fig. 2).

**Phylogenetic relation:** A young member of the family Varanopidae [8, 9].

1. ***Pyozia mesenensis***

**Where:** Northwestern Russian Federation [125].

**When:** Mezen Assemblage, lower Tatarian, Urzhumian [125], Wordian (see Fig. 2).

**Phylogenetic relation:** A young and primitive member of the family Varanopidae [8, 9].

**Amniote chrone 10 (Am10)**

1. ***Elliotsmithia longiceps***

**Where:** Abrahamskraal Farm, Prince Albert, South Africa [126].

**When:** *Tapinocephalus* Assemblage zone [126], Capitanian (see Fig. 2).

**Phylogenetic relation:** A young and primitive member of the family Varanopidae, and closely related to *Heleosaurus scholtzi* [8, 9].

1. ***Heleosaurus scholtzi***

**Where:** Abrahamskraal Farm, Prince Albert, South Africa [127].

**When:** *Tapinocephalus* or *Tropidostoma* Assemblage zone [127], Capitanian (see Fig. 2).

**Phylogenetic relation:** A young and primitive member of the family Varanopidae and closely related to *Elliotsmithia longiceps* [8, 9].

1. ***Anningia megalops***

**Where:** Bloukrans, Prince Albert, South Africa [128].

**When:** Uppermost Abrahamskraal Formation and lowermost Poortjie Member of the Teekloof Formation [128], Upper *Tapinocephalus* Assemblage zone, Capitanian (see Fig. 2).

**Phylogenetic relation:** A young and primitive member of the family Varanopidae and closely related to *Microvaranops parentis* [9].

1. ***Microvaranops parentis***

**Where:** Beukesplaas, Northern Cape, South Africa [9].

**When:** Upper *Tapinocephalus* Assemblage zone [9], Capitanian (see Fig. 2).

**Phylogenetic relation:** A young and primitive member of the family Varanopidae and closely related to *Elliotsmithia longiceps* [9].

**References**

1. Paleobiology Database. Search for *Protoclepsydrops haplous*. Access on February 9, 2020. <https://paleobiodb.org/classic/basicTaxonInfo?taxon_no=123523>
2. Haubold, H. *et al.* Interpretation of the tetrapod footprints from the Early Pennsylvanian of Alabama in *Pennsylvanian Footprints in the Black Warrior Basin of Alabama* (eds. Buta, R. J., Rindsberg, A. K. & Kopaska-Merkel, D. C.) 75–112 (Alabama Paleontological Society Monograph 1, 2005).
3. Reisz, R. R. Pelycosaurian reptiles from the middle Pennsylvanian of North America. *Bull Mus Comp Zool* **144**, 27–67 (1972).
4. Paleobiology Database. Search for *Archaeothyris*. Access on May 10, 2020. <https://paleobiodb.org/classic/basicTaxonInfo?taxon_no=38885>
5. Martino, R. L. Sequence stratigraphy of the Glenshaw Formation (Middle– Late Pennsylvanian) in the Central Appalachian Basin. *AAPG Studies in Geology* **51**, 1–28 (2004).
6. Benson, R. B. J. Interrelationships of basal synapsids: Cranial and postcranial morphological partitions suggest different topologies. *J Syst Palaeontol* **10**, 601–624 (2012).
7. Brocklehurst, N. & Fröbisch, J. A reexamination of *Milosaurus mccordi*, and the evolution of large body size in Carboniferous synapsids. *J Vertebr Paleontol*, e1508026 (2018).
8. Brocklehurst, N., Reisz, R.R., Fernandez, V. & Fröbisch, J. A re-description of ‘*Mycterosaurus*’ *smithae*, an Early Permian eothyridid, and its impact on the phylogeny of pelycosaurian-grade synapsids. *PLoS ONE* **11**, e0156810; <https://doi.org/10.1371/journal.pone.0156810> (2016).
9. Spindler, F. *et al.* First arboreal ’pelycosaurs’ (Synapsida: Varanopidae) from the early Permian Chemnitz Fossil Lagerstätte, SE Germany, with a review of varanopid phylogeny. *PalZ* **92**, 315–364 (2018).
10. Ezcurra, M. D., Scheyer, T. M. & Butler, R. J. The origin and early evolution of Sauria: reassessing the Permian saurian fossil record and the timing of the crocodile-lizard divergence. *PLoS ONE* **9**, e89165; <https://doi.org/10.1371/journal.pone.0089165> (2014).
11. Paleobiology Database. Search for *Echinerpeton*. Access on May 10, 2020. <https://paleobiodb.org/classic/basicTaxonInfo?taxon_no=38923>
12. Paleobiology Database. Search for *Clepsydrops*. Access on May 10, 2020. <https://paleobiodb.org/classic/basicTaxonInfo?taxon_no=38887>
13. Romer, A. S. & Price, L. W. *Review of the Pelycosauria* (Geological Society of America Special Papers 28, 1940).
14. Romer, A. S. A large ophiacodont pelycosaur from the Pennsylvanian of the Pittsburgh region. *Breviora* **144**, 1–7 (1961).
15. Laurin, M. & de Buffrénil, V. Microstructural features of the femur in early ophiacodontids: A reappraisal of ancestral habitat use and lifestyle of amniotes. *C R Palevol* **15**, 115–127 (2016).
16. Paleobiology Database. Search for *Ianthodon*. Access on May 10, 2020. <https://paleobiodb.org/classic/basicTaxonInfo?taxon_no=196919>
17. Spindler, F. The basal Sphenacodontia - systematic revision and evolutionary implications. PhD Thesis, TU Bergakademie Freiberg (2015).
18. DeMar, R. A primitive pelycosaur from the Pennsylvanian of Illinois. *J Paleontol* **44**, 154–163 (1970).
19. Shaver, R. H. *et al.* *Compendium of Paleozoic rock-unit stratigraphy in Indiana - a revision* (Indiana Geological Survey Bulletin 59, 1986).
20. Paleobiology Database. Search for *Xyrospondylus*. Access on May 10, 2020. <https://paleobiodb.org/classic/basicTaxonInfo?taxon_no=38934>
21. Paleobiology Database. Search for *Haptodus garnettensis*. Access on May 10, 2020. <https://paleobiodb.org/classic/basicTaxonInfo?taxon_no=122358>
22. Paleobiology Database. Search for *Ianthasaurus*. Access on May 10, 2020. <https://paleobiodb.org/classic/basicTaxonInfo?taxon_no=38912>
23. Mazierski, D. M. & Reisz, R. R. Description of a new specimen of *Ianthasaurus hardestiorum* (Eupelycosauria: Edaphosauridae) and a re-evaluation of edaphosaurid phylogeny. *Can J Earth Sci* **47**, 901–912 (2010).
24. Paleobiology Database. Search for *Ophiacodon navajovicus*. Access on May 10, 2020. <https://paleobiodb.org/classic/basicTaxonInfo?taxon_no=122334>
25. Lucas, S. G. Global Permian tetrapod biostratigraphy and biochronology. *Geol Soc Spec Publ* **265**, 405–444 (2006).
26. Vaughn, P. P. Vertebrates from the Halgaito Tongue of the Cutler Formation, Permian of San Juan County, Utah. *J Paleontol* **36**, 529–539 (1962).
27. DiMichele, W. A., Cecil, C. B., Chaney, D. S., Elrick, S. D. & Nelson, W. J. Fossil floras from the Pennsylvanian-Permian Cutler Group of southeastern Utah in *Geology of Utah’s Far South* (eds. MacLean, J. S., Biek, R. F. & Huntoon, J. E.) 491–504 (Utah Geological Association Publication 43, 2014).
28. Romer, A. S. New genera and species of pelycosaurian reptiles. *Proceedings of the New England Zoölogical Club* **16**, 89–95 (1937).
29. Romer, A. S. Early history of Texas redbeds vertebrates. *GSA Bulletin* **46**, 1597–1657 (1935).
30. Hentz, T. F., Jr. & Brown, L. F. *Geologic Atlas of Texas, Wichita Falls-Lawton Sheet* (University of Texas at Austin, 1987).
31. Brinkman, D. & Eberth, D. A. The anatomy and relationships of *Stereophallodon* and *Baldwinonus* (Reptilia, Pelycosauria). *Breviora* **485**, 1–34 (1986).
32. Reisz, R. R. & Dilkes, D. W. *Archaeovenator hamiltonensis*, a new varanopid (Synapsida: Eupelycosauria) from the Upper Carboniferous of Kansas. *Can J Earth Sci* **40**, 667–678 (2003).
33. Berman, D. S., Henrici, A.C., Sumida, S. S., Martens, T. & Pelletier, V. First European record of a Varanodontine (Synapsida: Varanopidae): Member of a unique Early Permian upland paleoecosystem, Tambach Basin, Central Germany in *Early Evolutionary History of the Synapsida* (eds. Kammerer, C. F., Angielczyk, K. D. & Fröbisch, J.) 69–86 (Springer, Netherlands, 2014).
34. Lucas, S. G., Harris, S. K., Spielmann, J. A., Berman, D. S. & Henrici, A. C. Vertebrate biostratigraphy and biochronology of the Pennsylvanian-Permian Cutler Group, El Cobre Canyon, Northern New Mexico. *New Mexico Museum of Natural History and Science Bulletin* **31**, 128–139 (2005).
35. Reisz, R. R. & Fröbisch, J. The oldest caseid synapsid from the Late Pennsylvanian of Kansas, and the evolution of herbivory in terrestrial vertebrates. *PLoS ONE* **9**, e94518; <https://doi.org/10.1371/journal.pone.0094518> (2014).
36. Spindler, F., Falconnet, J. & Fröbisch, J. *Callibrachion* and *Datheosaurus*, two historical and previously mistaken basal caseasaurian synapsids from Europe. *Acta Palaeontol Pol* **61**, 597–616; <https://doi.org/10.4202/app.00221.2015> (2016).
37. Paleobiology Database. Search for *Edaphosaurus mirabilis*. Access on May 10, 2020. <https://paleobiodb.org/classic/basicTaxonInfo?taxon_no=346037>
38. Opluštil, S., Schmitz, M., Cleal, C. J., & Martínek, K. A review of the Middle–Late Pennsylvanian west European regional substages and floral biozones, and their correlation to the Geological Time Scale based on new U–Pb ages. *Earth Sci Rev* **154**, 301–335 (2016).
39. Berman, D.S. *Edaphosaurus* (Reptilia, Pelycosauria) from the Lower Permian of northeastern United States, with description of a new species. *Ann Carnegie Mus* **48**, 185–202 (1979).
40. Milici, R. C. Assessment of Appalachian Basin oil and gas resources: Carboniferous Coal-bed Gas Total Petroleum System (U.S. Geological Survey Open-File Report 2004-1272); <https://pubs.usgs.gov/of/2004/1272/> (2004).
41. Modesto, S. P. & Reisz, R. R. Restudy of Permo-Carboniferous synapsid *Edaphosaurus novomexicanus* Williston and Case, the oldest known herbivorous amniote. *Can J Earth Sci* **29**, 2653–2662 (1992).
42. Paleobiology Database 11. Search for *Edaphosaurus novomexicanus*. Access on May 10, 2020. <https://paleobiodb.org/classic/basicTaxonInfo?taxon_no=122351>
43. Lucas, S. G., Krainer, K., Voigt, S., Berman, D. S. & Henrici, A. The Lower Permian Abo Formation in the northern Sacramento Mountains, southern New Mexico. New Mexico Geological Society 65th Annual Field Conference, Geology of the Sacramento Mountains Region. *New Mexico Geological Society Fall Field Conference Guidebook* **65**, 287–302 (2014).
44. Fröbisch, J., Schoch, R. R., Müller, J., Schindler, T. & Schweiss, D. A new basal sphenacodontid synapsid from the Late Carboniferous of the Saar-Nahe Basin, Germany. *Acta Palaeontol Pol* **56**, 113–120; <http://dx.doi.org/10.4202/app.2010.0039> (2011).
45. Uhl, D. & Jasper, A. New data on the macroflora of the basal Rotliegend Group (Remigiusberg Formation; Gzhelian) in the Saar-Nahe Basin (SW-Germany). *Fossil Imprint* **72**, 239–250 (2016).
46. Paleobiology Database. Search for *Macromerion schwartzenbergii*. Access on May 11, 2020. <https://paleobiodb.org/classic/basicTaxonInfo?taxon_no=334675>
47. Romer, A. S. The late Carboniferous vertebrate fauna of Kounova (Bohemia) compared with that of the Texas redbeds. *Am J Sci* **243**, 417–442 (1945).
48. Falconnet, J. The sphenacodontid synapsid *Neosaurus cynodus*, and related material, from the Permo-Carboniferous of France. *Acta Palaeontol Pol* **60**, 169–182; <https://doi.org/10.4202/app.2012.0105> (2015).
49. Schneider, J., Lucas, S. G., Werneburg, R. & Rößler, R. Euramerican Late Pennsylvanian/Early Permian arthropleurid/tetrapod associations – implications for the habitat and paleobiology of the largest terrestrial arthropod. *New Mexico Museum of Natural History and Science Bulletin* **49**, 49–70 (2010).
50. Spindler, F. Morphological description and taxonomic status of *Palaeohatteria* and *Pantelosaurus* (Synapsida: Sphenacodontia). *Freiberger Forschungshefte* **C 550**, 1–57 (2016).
51. Zieger, J. U–Pb ages of magmatic and detrital zircon of the Döhlen Basin: geological history of a Permian strike-slip basin in the Elbe Zone (Germany). *Int J Earth Sci* **108**, 887–910 (2019).
52. Spindler, F., Scott, D. & Reisz, R. R. New information on the cranial and postcranial anatomy of the early synapsid *Ianthodon schultzei* (Sphenacomorpha: Sphenacodontia), and its evolutionary significance. *Mitt Mus Nat Berl Foss Rec* **18**, 17–30 (2015).
53. Paleobiology Database. Search for *Sphenacodon britannicus*. Access on May 11, 2020. <https://paleobiodb.org/classic/basicTaxonInfo?taxon_no=147996>
54. Spielmann, J. A. *et al.* Redescription of the cranial anatomy of *Sphenacodon ferox* Marsh (Eupelycosauria: Sphenacodontidae) from the Late Pennsylvanian-Early Permian of New Mexico. *New Mexico Museum of Natural History and Science Bulletin* **49**, 159–184 (2010).
55. Spielmann, J. A. & Lucas, S. G. Re-evaluation of *Ruthiromia elcobriensis* (Eupelycosauria: Ophiacodontidae?) from the Lower Permian (Seymourian?) of Caρon del Cobre, Northern New Mexico. *New Mexico Museum of Natural History and Science Bulletin* **49**, 151–158 (2010).
56. Lucas, S. G. Permian tetrapod biochronology, correlation and evolutionary events. *Geol Soc Spec Publ* **450**, 405–444 (2017).
57. Lucas, S. G. *et al.* Lithostratigraphy, paleontology, biostratigraphy, and age of the Upper Paleozoic Abo Formation Near Jemez Springs, northern New Mexico, USA. *Ann Carnegie Mus* **80**, 323–350 (2012).
58. Brink, K. S. & Reisz, R. R. Hidden dental diversity in the oldest terrestrial apex predator Dimetrodon. *Nat Commun* **5**, 3269; <https://doi.org/10.1038/ncomms4269> (2014).
59. Brink, K. S., Maddin, H. C., Evans, D. C. & Reisz, R. R. Re-evaluation of the historic Canadian fossil *Bathygnathus borealis* from the Early Permian of Prince Edward Island. *Can J Earth Sci* **52**, 1109–1120 (2015).
60. Harris, S. K., Lucas, S. G., Berman, D. S., & Henrici, A. C. Vertebrate fossil assemblage from the Upper Pennsylvanian Red Tanks Member of the Bursum Formation, Lucero Uplift, Central New Mexico. *New Mexico Museum of Natural History and Science Bulletin* **25**, 267–284 (2004).
61. Lucas, S. G., Rinehart, L. F. & Celeskey, M. D. The oldest specialized tetrapod herbivore: A new eupelycosaur from the Permian of New Mexico, USA. *Palaeontol Electronica* 21.3.39A; <https://doi.org/10.26879/899> (2018).
62. Lewis, G. E. & Vaughn, P. P. *Early Permian Vertebrates from the Culter Formation of the Placerville Area, Colorado, with a Section on Footprints from the Cutler Formation* (Geological Survey Professional Paper 503- C, 1965).
63. Condon, S. M. *Geology of the Pennsylvanian and Permian Cutler Group and Permian Kaibab Limestone in the Paradox Basin, Southeastern Utah and Southwestern Colorado U.S* (Geological Survey Bulletin 2000-P, 1997).
64. Moore, K. D., Soreghan, G. S. & Sweet, D. E. Stratigraphic and structural relations in the proximal Cutler Formation of the Paradox Basin: implications for timing of movement on the uncompahgre front. *The Mountain Geologist* **45**, 49–68 (2008).
65. Schneider, J. W. Environment, biotas and taphonomy of the Lower Permian lacustrine Niederhäslich limestone, Döhlen basin, Germany. *Earth Environ Sci Trans R Soc Edinb* **84**, 453–464 (1994).
66. Roscher, M. & Schneider, H. W. An annotated correlation chart for continental Late Pennsylvanian and Permian basins and the marine. *New Mexico Museum of Natural History and Science Bulletin* **30**, 282–291 (2005).
67. Werneburg, R. & Schneider, J. W. Non-marine Permian biostratigraphy and biochronology. *Geol Soc Spec Publ* **265**, 201–215 (2006).
68. Schneider, J. W. & Task Group. Report of the nonmarine-marine correlation working group. *Newsletter on Carboniferous Stratigraphy* **32**, 38–41; <http://carboniferous.stratigraphy.org/files/20170331203916776.pdf> (2016).
69. Sander, P. M. Early Permian depositional environments and pond bonebeds in central archer County, Texas. *Palaeogeogr Palaeoclimatol Palaeoecol* **69**, 1–21 (1989).
70. Romer, A. S. Vertebrate faunal horizons in the Texas Permo-Carboniferous red beds. *University of Texas Bulletin* ***2801***, 67–108 (1928).
71. Berman, D. S. A new species of *Dimetrodon* (Reptilia: Pelycosauria) from the Lower Permian of north-central New Mexico. *J Paleontol* **51**, 108–115 (1977).
72. Berman, D. S, Reisz, R. R., Martens, T. & Henrici, A. C. A new species of *Dimetrodon* (Synapsida: Sphenacodontidae) from the Lower Permian of Germany records first occurrence of genus outside of North America. *Can J Earth Sci* **38**, 803–812 (2001).
73. Vaughn, P. P. Early Permian vertebrates from southern New Mexico and their paleozoogeographic significance. *Los Angeles County Museum of Natural History,* *Contributions in Science* **166,** 1–22 (1969).
74. Lucas, S. G. *et al.* The Lower Permian Abo Formation in the Fra Cristobal and Caballo Mountains, Sierra County, New Mexico in *Geology of the Warm Springs Region* (eds. Lucas, S. G., McLemore, V. T., Lueth, V. W., Spielmann, J. A. & Krainer, K.) 345–376 (New Mexico Geological Society 63rd Annual Field Conference Guidebook, 2012).
75. Sumida, S. S. New information on the pectoral girdle and vertebral column in Lupeosaurus (Reptilia, Pelycosauria). *Can J Earth Sci* **26**, 1343–1349 (1989).
76. Clark, J. & Carroll, R. L. Romeriid reptiles from the Lower Permian. *Bull Mus Comp Zool* **144**, 353-407 (1973).
77. Huttenlocker, A. K., Mazierski, D. & Reisz, R. R. Comparative osteohistology of hyperelongate neural spines in the Edaphosauridae (Amniota: Synapsida). *Palaeontology* **54**, 573–590 (2011).
78. Sander, P. M. Taphonomy of the Lower Permian Geraldine Bonebed in Archer County, Texas. *Palaeogeogr Palaeoclimatol Palaeoecol* **61**, 221–236 (1987).
79. Kissel, R. Morphology, phylogeny, and evolution of Diadectidae (Cotylosauria: Diadectomorpha). PhD Thesis, University of Toronto; <http://hdl.handle.net/1807/24357> (2010).
80. Fedorko, N. & Skema, V. A review of the stratigraphy and stratigraphic nomenclature of the Dunkard Group in West Virginia and Pennsylvania, USA. *Int J Coal Geol* **119**, 2–20 (2013).
81. Smith, W. H. *Geology of Newport Township, Washington County, Ohio* (Report of investigations 5, Geological Survey of Ohio, 1948).
82. Reisz, R. R., Maddin, H. C., Fröbisch, J. & Falconnet, J. A new large caseid (Synapsida, Caseasauria) from the Permian of Rodez (France), including a reappraisal of *“Casea” rutena* Sigogneau-Russell & Russell, 1974. *Geodiversitas* **33**, 227–246 (2011).
83. Langston, W., Jr. *Oedalops campi* (Reptilia: Pelycosauria) new genus and species from the Lower Permian of New Mexico, and the family Eothyrididae. *Texas Memorial Museum Bulletin* **9**, 1-47 (1965).
84. Laurin, M. The osteology of a Lower Permian eosuchian from Texas and a review of diapsid phylogeny. *Zool J Linn Soc* **101**, 59–95 (1991).
85. Reisz, R. R., Laurin, M. & Marjanović, D. *“Apsisaurus witteri”* from the Lower Permian of Texas: yet another small varanopid synapsid, not a diapsid. *J Vertebr Paleontol* **30**, 1628–1631 (2010).
86. Eberth, D. S. & Brinkman, D. *Ruthiromia elcobriensis*, a new pelycosaur from El Cobre Canyon, New Mexico. *Breviora* **474**, 1–26 (1983).
87. Langston, W. & Reisz, R. R. *Aerosaurus wellesi*, new species, a varanopseid mammal-like reptile (Synapsida: Pelycosauria) from the Lower Permian of New Mexico. *J Vertebr Paleontol* **1**, 73–96 (1981).
88. Paleobiology Database. Search for *Ophiacodon uniformis*. Access on May 11, 2020. <https://paleobiodb.org/classic/basicTaxonInfo?taxon_no=123419>
89. Olson, E. C. Early Permian vertebrates of Oklahoma. *Oklahoma Geological Survey, Circular* **74**, 1-111 (1967).
90. Heran, W. D., Green, G. N. & Stoeser, D. B. *A Digital Geologic Map Database for the State of Oklahoma* (Geological Survey Open-File Report 03-247, 2003).
91. Paleobiology Database. Search for *Ophiacodon retroversus*. Access on May 11, 2020. <https://paleobiodb.org/classic/basicTaxonInfo?taxon_no=138048>
92. Paleobiology Database. Search for *Edaphosaurus boanerges*. Access on May 11, 2020. <https://paleobiodb.org/classic/basicTaxonInfo?taxon_no=122352>
93. Romer, A. S. Late Pennsylvanian and Early Permian vertebrates of the Pittsburgh-West Virginia region. *Ann Carnegie Mus* **33**, 47–110 (1952).
94. Modesto, S. P. The skull of the herbivorous synapsid *Edaphosaurus boanerges* from the Lower Permian of Texas. *Palaeontology* **38**, 213–239 (1995).
95. Hook, R. W. & Hotton, N. A new sphenacodontid pelycosaur (Synapsida) from the Wichita Group, Lower Permian of North-Central Texas. *J Vertebr Paleontol* **11**, 37–44 (1991).
96. Paleobiology Database. Search for *Dimetrodon limbatus*. Access on May 11, 2020. <https://paleobiodb.org/classic/basicTaxonInfo?taxon_no=70401>
97. Paleobiology Database. Search for *Dimetrodon booneorum*. Access on May 11, 2020. <https://paleobiodb.org/classic/basicTaxonInfo?taxon_no=122783>
98. Shelton, C. D., Sander, P. M., Stein, K. & Winkelhorst, H. Long bone histology indicates sympatric species of *Dimetrodon* (Lower Permian, Sphenacodontidae). *Earth Environ Sci Trans R Soc Edinb* **103**, 217–236 (2013).
99. Paleobiology Database. Search for *Dimetrodon natalis*. Access on May 11, 2020. <https://paleobiodb.org/classic/basicTaxonInfo?taxon_no=122781>
100. Vaughn, P. P. Vertebrates from the Organ Rock Shale of the Cutler Group, Permian of Monument Valley and vicinity, Utah and Arizona. *J Paleontol* **38,** 567-583 (1964).
101. Cain, S. A. & Mountney, N. P. Spatial and temporal evolution of a terminal fluvial fan system: the Permian Organ Rock Formation, South‐east Utah, USA. *Sedimentology* **56**, 1774-1800 (2009). <https://doi.org/10.1111/j.1365-3091.2009.01057.x>
102. Berman, D. S. *Ctenospondylus ninehevensis*, a new species (Reptilia, Pelycosauria) from the Lower Permian Dunkard Group of Ohio. *Ann Carnegie Mus* **47**, 493–514 (1978).
103. Fedorko, N. & Skema, V. A review of the stratigraphy and stratigraphic nomenclature of the Dunkard Group in West Virginia and Pennsylvania, USA. *Int J Coal Geol* **119**, 2–20 (2013).
104. Paleobiology Database. Search for *Dimetrodon loomisi*. Access on May 11, 2020. <https://paleobiodb.org/classic/basicTaxonInfo?taxon_no=90738>
105. Paleobiology Database. Search for *Dimetrodon gigashomogenes*. Access on February 10, 2020. <https://paleobiodb.org/classic/basicTaxonInfo?taxon_no=138123>
106. Olson, E. C. *Late Permian terrestrial vertebrates, U.S.A. and U.S.S.R.* (Transactions of the American Philosophical Society, New Series, 52, 1962).
107. Berman, D. S., Henrici, A. C., Sumida, S. S. & Martens, T. New materials of *Dimetrodon teutonis* (Synapsida: Sphenacodontidae) from the Lower Permian of Germany. *Ann Carnegie Mus* **73**, 108–116 (2004).
108. Brink, K. S., LeBlanc, A. R. H. & Reisz, R. R. First record of plicidentine in Synapsida and patterns of tooth root shape change in Early Permian sphenacodontians. *Naturwissenschaften* **101**, 883–892 (2014).
109. Reisz, R. R. Pelycosauria in *Handbuch der Paläoherpetologie* (ed. Wellnhofer, P.) Part 17A (Gustav Fischer Verlag, Stuttgart, 1986).
110. Paleobiology Database. Search for *Edaphosaurus cruciger*. Access on May 11, 2020. <https://paleobiodb.org/classic/basicTaxonInfo?taxon_no=138134>
111. Reisz, R. R. *Oromycter*, a new caseid from the Lower Permian of Oklahoma. *J Vertebr Paleontol* **25**, 905–910 (2005).
112. Olson, E. C. & Barghusen, H. Permian vertebrates from Oklahoma and Texas. Part I.—Vertebrates from the Flowerpot Formation, Permian of Oklahoma. *Oklahoma Geological Survey, Circular* **59** 5–48 (1962).
113. Olson, E. C. & Beerbower, J. R. The San Angelo Formation, Permian of Texas, and its vertebrates. *J Geol* **61**, 389–423 (1953).
114. Olson, E. C. Fauna of the Vale and Choza: 7, Pelycosauria: family Caseidae. *Fieldiana: Geology* **10** (17), 193–204 (1954).
115. Paleobiology Database. Search for *Casea nicholsi*. Access on May 11, 2020. <https://paleobiodb.org/classic/basicTaxonInfo?taxon_no=120853>
116. Paleobiology Database. Search for *Cotylorhynchus hancocki*. Access on May 11, 2020. <https://paleobiodb.org/classic/basicTaxonInfo?taxon_no=122303>
117. Paleobiology Database. Search for *Cotylorhynchus bransoni*. Access on May 11, 2020. <https://paleobiodb.org/classic/basicTaxonInfo?taxon_no=120922>
118. Reisz, R. R. & Tsuji, L. A. An articulated skeleton of *Varanops* with bite marks: the oldest known evidence of scavenging among terrestrial vertebrates. *J Vertebr Paleontol* **26**, 1021–1023 (2006).
119. Campione, N. E. & Reisz, R. R. *Varanops brevirostris* (Eupelycosauria: Varanopidae) from the Lower Permian of Texas, with discussion of varanopid morphology and interrelationships. *J Vertebr Paleontol* **30**, 724–746 (2010).
120. Maddin, H. C., Evans, D. C. & Reisz, R. R. An Early Permian Varanodontine Varanopid (Synapsida: Eupelycosauria) from the Richards Spur locality, Oklahoma. *J Vertebr Paleontol* **26**, 957–966 (2006).
121. Olson, E. C. New Permian vertebrates from the Chickasha Formation in Oklahoma. *Oklahoma Geological Survey, Circular* **70**, 1-70 (1965).
122. Olson, E. C. On the source of therapsids. *Annals of the South African Museum* **64**, 27–46 (1974).
123. Brocklehurst, N. & Fröbisch, J. A re-examination of the enigmatic Russian tetrapod *Phreatophasma aenigmaticum* and its evolutionary implications. *Mitt Mus Nat Berl Foss Rec* **20**, 87–93 (2017).
124. Maddin, H.C., Sidor, C. A. & Reisz, R. R. Cranial anatomy of *Ennatosaurus tecton* (synapsida: Caseidae) from the Middle Permian of Russia and the evolutionary relationships of Caseidae. *J Vertebr Paleontol* **28**, 160–180 (2008).
125. Anderson, J. S. & Reisz, R. R. *Pyozia mesenensis*, a new, small vapanopid (Synapsida: Eupelycosauria) from Russia: ‘‘Pelycosaur’’ Diversity in the Middle Permian. *J Vertebr Paleontol* **24**, 173–179 (2004).
126. Paleobiology Database. Search for *Elliotsmithia longiceps*. Access on May 11, 2020. <https://paleobiodb.org/classic/basicTaxonInfo?taxon_no=140622>
127. Reisz, R. R. & Modesto, S. P. *Heleosaurus scholtzi* from the Permian of South Africa: a varanopid synapsid, not a diapsid reptile. *J Vertebr Paleontol* **27**, 734–739 (2007).
128. Paleobiology Database. Search for *Anningia megalops*. Access on May 12, 2020. <https://paleobiodb.org/classic/basicTaxonInfo?taxon_no=148038>

**Appendix S2: Early Therapsid stratigraphy and phylogenetic relations**

**Synapsida, Therapsida**

**Dinocephalia**

**Amniote chrone 8 (Am8)**

1. ***Syodon biarmicum* (LGU 140/1)**

**Where:** Exact locality unknown, Perm Krai, Russian Federation [1, 2].

**When:** Sheshmian Horizon, Ufimian [1, 2; for a general geology of the area, see ref. 3], earliest Roadian (see the Methods, Fig. 3 and ref. [4]).

**Phylogenetic relation:** Closely related to *Australosyodon* within Syodontinae, Dinocephalia [1, 5].

**Notice:** The holotype of this taxon is based exclusively on the LGU 140/1, an isolated left canine, which is the only anteosaur specimen of Ufimian age [1, and references therein]. It is the oldest therapsid specimen known.

**Amniote chrone 9 (Am9)**

1. ***Syodon “biarmicum”*** [1]

**Where:** Orenburg and Tatarstan, Russian Federation [2].

**When:** Isheevo and Ocher Assemblages [2], Urzhumian to earliest Severodvinian (see Fig. 2).

**Phylogenetic relation:** A dinocephalian closely related to *Australosyodon* within Syodontinae [1, 5].

**Notice:** Kammerer [1] considered *S*. *efremovi* to be a junior synonym of *S. biarmicum* because of the problematic characteristics used to separate *Syodon biarmicum* from *S*. *efremovi*. Here, we kept them as two species based on the much older age of the LGU 140/1, which was not personally examined by Kammerer [1]. *S. biarmicum* is closely related to *A. nyaphuli* within the Syodontinae [1, 5].

1. ***Titanophoneus adamanteus***

**Where:** Malyi Uran locality, Orenburg Region, Russian Federation [6].

**When:** *Titanophoneus* other zone, Isheevo Subassemblage [6] (see Fig. 2).

**Phylogenetic relation:** A dinocephalian closely related to *T. potens*, *Anteosaurus magnificus*, and *Sinophoneus yumenensis* within Anteosaurinae [1, 5, 7].

1. ***Titanophoneus potens***

**Where:** Isheevo locality, Apastovskii District, Russian Federation [6].

**When:** Collections PIN 157 and 2207, Isheevo Faunal Subassemblage [6], Urzhumian to lowermost Severodvinian (see Fig. 2).

**Phylogenetic relation:** A dinocephalian closely related to *T. adamanteus*, *Anteosaurus magnificus*, and *Sinophoneus yumenensis* within Anteosaurinae [1, 5, 7].

1. ***Anteosaurus “rugosus”* (=*Titanophoneus rugosus*)**

**Where:** Butlerovka locality, Tatarstan, Russian Federation [1, 6].

**When:** Isheevo Faunal Subassemblage, Urzhumian [6] (see Fig. 2).

**Phylogenetic relation:** Indeterminate dinocephalian anteosaur that indicates the presence of the genus *Anteosaurus* in Russia [1].

1. ***Australosyodon nyaphuli***

**Where:** Tuinkraal, Prince Albert, South Africa [8].

**When:** *Eodicynodon* Assemblage Zone [8], uppermost Wordian- lowermost Capitanian [9] (see Fig. 2).

**Phylogenetic relation:** A dinocephalian closely related to *Syodon biarmicum* within Syodontinae [1, 5].

1. ***Notosyodon gusevi***

**Where:** Zhaksy-Kargala, Aktyubinsk, Kazakhstan [6].

**When:** Collection PIN 2505, *Deuterosaurus biarmicus* other zone, Malaya Kinel Subassemblage of the Isheevo Assemblage [6], upper Urzhumian (see Fig. 2).

**Phylogenetic relation:** A dinocephalian closely related to the *Syodon biarmicum* + *Australosyodon nyaphuli* clade within Syodontinae [1, 5].

1. ***Archaeosyodon praeventor***

**Where:** Ezhovo locality, Perm, Russian Federation [6].

**When:** *Estemmenosuchus uralensis* other zone, Ocher Assemblage [6], lower Urzhumian (see Fig. 2).

**Phylogenetic relation:** A dinocephalian closely related to *Microsyodon orlovi*, which is outside of the Anteosaurinae and Syodontinae [1] (see also phylogenetic tree B in Supplementary Material of ref. 5). Ivakhnenko [10] placed it in Titanosuchidae.

1. ***Microsyodon orlovi***

**Where:** Golyusherma locality, Udmurtia, Russian Federation [6].

**When:** Collection PIN 4276, Ocher Faunal Assemblage, Golyusherma Subassemblage [6], upper Kazanian (nonmarine), upper Roadian [4].

**Phylogenetic relation:** A dinocephalian closely related to *Archaeosyodon praeventor*, which is outside of the Anteosaurinae and Syodontinae [1] (see also phylogenetic tree B in Supplementary Material of ref. 5). Ivakhnenko [10] placed it in Titanosuchidae.

1. ***Ulemosaurus svijagensis***

**Where:** Isheevo locality, Apastovskii District, Russian Federation [6].

**When:** Collections PIN 157 and 2207, Isheevo Faunal Subassemblage [6], Urzhumian to lowermost Severodvinian (see Fig. 2).

**Phylogenetic relation:** A dinocephalian closely related to *Tapinocaninus pamelae* [5, 7].

1. ***Tapinocaninus pamelae***

**Where:** Modderdrift farm, Prince Albert, South Africa [11].

**When:** *Eodicynodon* other zone [12], uppermost Wordian- lowermost Capitanian [9] (see Fig. 2).

**Phylogenetic relation:** A dinocephalian closely related to *Estemmenosuchus*, which is outside of the Anteosaurinae and Syodontinae and within Tapinocephalidae [1].

1. ***Estemmenosuchus uralensis***

**Where:** Ezhovo locality, Perm, Russian Federation [6].

**When:** *Estemmenosuchus uralensis* other zone, Ocher Assemblage [6], lower Urzhumian [4] (see Fig. 2).

**Phylogenetic relation:** A dinocephalian closely related to the *Tapinocaninus pamelae* + *Ulemosaurus svijagensis* clade [5].

1. ***Estemmenosuchus mirabilis***

**Where:** Ezhovo locality, Perm, Russian Federation [6].

**When:** *Estemmenosuchus uralensis* other zone, Ocher Assemblage [6], lower Urzhumian [4] (see Fig. 2).

**Phylogenetic relation:** A dinocephalian closely related to *Tapinocaninus pamelae* [1] or to the *Tapinocaninus pamelae* + *Ulemosaurus svijagensis* clade [7].

**Amniote chrone 10 (Am10)**

1. ***Anteosaurus magnificus***

**Where:** South Africa [13].

**When:** *Tapinocephalus* Assemblage zone, Capitanian [13].

**Phylogenetic relation:** A dinocephalian closely related to *T. adamanteus*, *T. potens*, and *Sinophoneus yumenensis* within Anteosaurinae [1, 5, 7].

1. ***Sinophoneus yumenensis***

**Where:** China [7].

**When:** Qingtoushan Formation [7], Gamkan, *Tapinocephalus-Pristerognathus* zone [4].

**Phylogenetic relation:** A dinocephalian closely related to *T. adamanteus*, *T. potens*, and *Anteosaurus magnificus* within Anteosaurinae [1, 5, 7].

1. ***Pampaphoneus biccai***

**Where:** Boqueirão farm, Rio Grande do Sul, Brasil [5].

**When:** Morro Pelado Member of the Rio do Rasto Formation [5], Gamkan, *Tapinocephalus-Pristerognathus* zone [4] (see Fig. 2).

**Phylogenetic relation:** A dinocephalian closely related to the *Syodon biarmicum* + *Australosyodon nyaphuli* clade and outside of *N. gusevi* within Syodontinae [5].

1. ***Struthiocephaloides cavifrons***

**Where:** South Africa. 1) Farm Lammerkraal, Prince Albert district, eastern Koup. 2) Farm Dikbome, Laingsburg district [14, and references therein].

**When:** The stratigraphic range of *S. cavifrons* can be constrained to an interval 2150–2450 m above the base of the Abrahamskraal Formation at its thickest point, which is equivalent to the Moordenaars Member [14], upper *Tapinocephalus* zone (see Fig. 2).

**Phylogenetic relation:** Carroll [15] considered *S. cavifrons* to be a tapinocephalid, and *Struthiocephaloides* was considered a valid genus by Day [14] and Day et al. [16]. However, its exact phylogenetic relation is unknown because it has never been included in a comprehensive phylogenetic study.

1. ***Mormosaurus seeleyi***

**Where:** Farm Klein Koedoeskop, Beaufort West district, South Africa [14, and references therein].

**When:** It can be stratigraphically constrained to an interval 2000–2100 m above the base of the Abrahamskraal Formation, which corresponds to the uppermost Moordenaars Member [14], upper *Tapinocephalus* zone (see Fig. 2).

**Phylogenetic relation:** Carroll [15] considered *M. seeleyi* to be a tapinocephalid, and *Mormosaurus* was considered a valid genus by Day [14] and Day et al. [16]. However, its exact phylogenetic relation is unknown because it has never been included in a comprehensive phylogenetic study.

1. ***Riebeeckosaurus longirostris***

**Where:** Viviers railway, Beaufort West district, South Africa [14, and references therein; 17].

**When:** It can be stratigraphically constrained to an interval 2300–2500 m above the base of the Abrahamskraal Formation, which corresponds to the upper Moordenaars Member [14], upper *Tapinocephalus* zone (see Fig. 2).

**Phylogenetic relation:** Carroll [15] considered *Riebeeckosaurus* as a tapinocephalid, and *Riebeeckosaurus* was considered a valid genus by Day [14], Day et al. [16], and Güven et al. [17] who synonymized it with *Avenantia kruisvleiensis*. However, its exact phylogenetic relation is unknown because it has never been included in a comprehensive phylogenetic study.

1. ***Delphinognathus conocephalus***

**Where:** South Africa. 1) Farm Kruisvallei, north of Merweville, Beaufort West district. 2) Farm Rietkuil, Beaufort West district [14, and references therein].

**When:** It can be stratigraphically constrained to an interval 2200–2550 m above the base of the Abrahamskraal Formation, which corresponds to the lower Moordenaars Member [14], upper *Tapinocephalus* zone (see Fig. 2).

**Phylogenetic relation:** Carroll [15] considered *D. conocephalus* to be a tapinocephalid, and *Delphinognathus* was considered a valid genus by Day [14] and Day et al. [16]. However, its exact phylogenetic relation is unknown because it has never been included in a comprehensive phylogenetic study.

1. ***Moschops sp.***

**Where:** Various localities of South Africa [14, and references therein].

**When:** The stratigraphic range of *Moschops* can be constrained to an interval 2100–2550 m above the base of the Abrahamskraal Formation at its thickest point, in the upper Swaerskraal Member, Moordenaars Member, and Karelskraal Member [14], upper *Tapinocephalus* zone (see Fig. 2).

**Phylogenetic relation:** Carroll [15] considered *Moschops* to be a tapinocephalid, and *Moschops* was considered a valid genus by Day [14] and Day et al. [16]. However, its exact phylogenetic relation is unknown because it has never been included in a comprehensive phylogenetic study.

1. ***Struthiocephalus sp.***

**Where:** Viviers railway, Beaufort West district, South Africa [14, and references therein].

**When:** The stratigraphic range of *Struthiocephalus* can be constrained to an interval 1600 m to 2550 m above the base of the Abrahamskraal Formation at its thickest point, which is equivalent to a stratigraphic interval from the upper Koornplaats Member to the upper Karelskraal Member [14], upper *Tapinocephalus* zone (see Fig. 2).

**Phylogenetic relation:** Carroll [15] considered *Struthiocephalus* to be a tapinocephalid, and *Struthiocephalus* was considered a valid genus by Day [14] and Day et al. [16]. However, its exact phylogenetic relation is unknown because it has never been included in a comprehensive phylogenetic study.

1. ***Keratocephalus moloch***

**Where:** Farms Boesmansrivier and Mynhardskraal, Beaufort West district, South Africa [14, and references therein].

**When:** It can be stratigraphically constrained to an interval 2050–2100 m above the base of the Abrahamskraal Formation, which corresponds to the upper Moordenaars Member or lowest Karelskraal Member [14], upper *Tapinocephalus* zone (see Fig. 2).

**Phylogenetic relation:** In Güven et al. [18] and Day [14], *K. moloch* was considered a junior synonym of *Struthiocephalus whaitsi*. Therefore, *Keratocephalus* is probably not valid genus and, thus it is not further considered here.

1. ***Taurocephalus lerouxi***

**Where:** Farm Abrahamskraal, Prince Albert district, South Africa [14, and references therein].

**When:** The stratigraphic range of *Taurocephalus* can be constrained to an interval 1800 m to 2200 m above the base of the Abrahamskraal Formation at its thickest point, which corresponding to the Swaerskraal Member [14], upper *Tapinocephalus* zone (see Fig. 2).

**Phylogenetic relation:** Carroll [15] considered *Taurocephalus* to be a tapinocephalid, and *Taurocephalus was* considered a valid genus by Day [14] and Day et al. [16]. However, its exact phylogenetic relation is unknown because it has never been included in a comprehensive phylogenetic study.

1. ***Struthionops sp.***

**Where:** Farm Abrahamskraal, Prince Albert district, South Africa [14, and references therein].

**When:** The stratigraphic range of *Struthionops* can be constrained to an interval 1800 m to 2200 m above the base of the Abrahamskraal Formation at its thickest point, which corresponds to the Swaerskraal Member [14], upper *Tapinocephalus* zone (see Fig. 2).

**Phylogenetic relation:** Carroll [15] considered *Struthionops* to be a tapinocephalid, and *Struthionops* was considered a valid genus by Day [14] and Day et al. [16]. However, its exact phylogenetic relation is unknown because it has never been included in a comprehensive phylogenetic study.

1. ***Phocosaurus sp.***

**Where:** Various localities of South Africa [14, and references therein].

**When:** The stratigraphic range of *Taurocephalus* can be constrained to an interval 2100 m to 2575 m above the base of the Abrahamskraal Formation at its thickest point, which encompasses the upper Swaerskraal Member, the Moordenaars Member, and the Karelskraal Member [14], upper *Tapinocephalus* zone (see Fig. 2).

**Phylogenetic relation:** *Phocosaurus* remains are a poorly defined taxon and is most likely a junior synonym of *Mormosaurus* [14]. Thus, it is not further considered here.

1. ***Criocephalosaurus vanderbyli***

**Where:** Various localities of South Africa [14, and references therein].

**When:** The stratigraphic range of *C. vanderbyli* is between 2100 m above the base of the Abrahamskraal Formation at its thickest point to the lower Poortjie Member of the Teekloof Formation [14], upper *Tapinocephalus* to lower *Pristerognathus* zone (see Fig. 2).

**Phylogenetic relation:** *Criocephalosaurus* was considered a valid tapinocephalid genus by Day [14] and Day et al. [16]. However, its exact phylogenetic relation is unknown because it has never been included in a comprehensive phylogenetic study.

1. ***Jonkeria sp.***

**Where:** Farm Abrahamskraal, Prince Albert district, South Africa [14, and references therein].

**When:** The stratigraphic range of *Jonkeria* is constrained to an interval 2100–2575 m above the base of the Abrahamskraal Formation at its thickest point, which is equivalent to the upper Swaerskraal Member, the Moordenaars Member, and the Karelskraal Member [14], upper *Tapinocephalus* zone (see Fig. 2).

**Phylogenetic relation:** *Jonkeria* was considered a titanosuchid by Carroll [15] and a valid genus by Day [14] and Day et al. [16]. However, its exact phylogenetic relation is unknown because it has never been considered in a comprehensive phylogenetic study even though eight species of this genus have been described [14, and references therein]. Although some of the species are probably synonyms, some of the other species may be valid.

1. ***Titanosuchus ferox***

**Where:** Various localities of South Africa [14, and references therein].

**When:** The stratigraphic range of *Titanosuchus* is constrained to an interval 2100–2575 m above the base of the Abrahamskraal Formation at its thickest point, which is equivalent to the upper Swaerskraal Member, the Moordenaars Member, and the Karelskraal Member [14], upper *Tapinocephalus* zone (see Fig. 2).

**Phylogenetic relation:** *Titanosuchus* was considered a titanosuchid by Carroll [15] and a valid genus by Day [14] and Day et al. [16]. However, its exact phylogenetic relation is unknown because it has never been considered in a comprehensive phylogenetic study.

1. ***Styracocephalus platyrhynchus***

**Where:** Various localities of South Africa [14, and references therein].

**When:** The stratigraphic range of *Styracocephalus* is constrained to an interval 2150–2575 m above the base of the Abrahamskraal Formation at its thickest point, which is equivalent to the Moordenaars and Karelskraal members [14], upper *Tapinocephalus* zone (see Fig. 2).

**Phylogenetic relation:** *S. platyrhynchus* has been considered a stem group tapinocephalian that is more closely related to the herbivorous tapinocephalids and titanosuchids than to the Anteosauridae.

**Anomodontia**

**Amniote chrone 9 (Am9)**

1. ***Patranomodon nyaphulii***

**Where:** Farm Combrinkskraal, Eastern Cape, South Africa [19].

**When:** *Eodicynodon* zone [19], uppermost Wordian- lowermost Capitanian [9] (see Fig. 2).

**Phylogenetic relation:** *P. nyaphulii* has been identified as a basal anomodont in the Anomocephalus + *Tiarajudens* clade, also associated with *B. qilianicus* [20, 21].

1. ***Otsheria netzvetajevi***

**Where:** Ezhovo locality, Perm, Russian Federation [6].

**When:** Collection PIN 1758, *Estemmenosuchus uralensis* other zone, Ocher Assemblage, Ocher Faunal Subassemblage [6], lower Urzhumian [4] (see Fig. 2).

**Phylogenetic relation:** *O. netzvetajevi* is a member of the anomodont family Venyukovioidea, which forms a separate clade from the *S. getmanovi* + *U. invisa* [22] clade or forms a clade with *U. invisa* that is separate from *S. getmanovi* ([21]. Here, the first interpretation is supported.

1. ***Ulemica invisa***

**Where:** Isheevo locality, Tatarstan, Russian Federation [6].

**When:** Collections PIN 157 and 2207 *Titanophoneus potens* other zone, Isheevo Assemblage, Isheevo Faunal Subassemblage [6], Urzhumian to lowermost Severodvinian (see Fig. 2).

**Phylogenetic relation:** *U. invisa* is a member of the anomodont family Venyukovioidea. *U. invisa* and *S. getmanovi* may form a clade separate from *O. netzvetajevi* [22] or *U. invisa* and *O. netzvetajevi* may form a clade separate from *S. getmanovi* [21]. Here, the first interpretation is supported.

1. ***Eodicynodon oosthuizeni***

**Where:** Southwest of the Karoo Basin, South Africa [14, and references therein].

**When:** *Eodicynodon* zone [14], uppermost Wordian to lowermost Capitanian [9].

**Phylogenetic relation:** In the most thorough phylogenetic studies of Dicynodontia [20, 21], *E. oosthuizeni* was determined to be a basal dicynodont together with *E. oelofseni* and was found to be the sister taxon to *Colobodectes cluveri* [20].

1. ***Eodicynodon oelofseni***

**Where:** Botterkraal, Prince Albert, South Africa [23].

**When:** *Eodicynodon* zone [23], uppermost Wordian to lowermost Capitanian [9].

**Phylogenetic relation:** *E. oelofseni* is a basal dicynodont anomodont ([21].

1. ***Lanthanostegus mohoii***

**Where:** Farm Mandalay, Jansenville district, eastern part of the Karoo Basin, South Africa [14, and references therein].

**When:** According to Day [14], the stratigraphic range of *Lanthanostegus mohoii* is equivalent to the *Eodicynodon* zone (see Fig. 2).

**Phylogenetic relation:** *L. mohoi* is a basal anomodont [21].

1. ***Brachyprosopus broomi***

**Where:** Various localities of South Africa [14, 24].

**When:** According to Day [14], the overall stratigraphic range of *Brachyprosopus* extends from the lower Abrahamskraal Formation, perhaps within the *Eodicynodon* AZ, up to the Karelskraal Member of the Abrahamskraal Formation at its thickest point (see Fig. 2).

**Phylogenetic relation:** *Chelydontops altidentalis* is a junior synonym of *B. broomi* [24].

**Amniote chrone 10 (Am10)**

1. ***Biseridens qilianicus***

**Where:** Dashankou locality, Gansu, China [25].

**When:** Qingtoushan Formation [25]. Although the age of this formation is not well-constrained, it is considered to be in the Gamkan, *Tapinocephalus-Pristerognathus* zone [4] (see Fig. 2).

**Phylogenetic relation:** *B. qilianicus* is a basal anomodont related to both the *Anomocephalus*+*Tiarajudens* clade and the *P. nyaphulii* [20, 21]. The close relation between *Anomocephalus* and *Tiarajudens* and *B. qilianicus* indicates that they are close in age*.* Thus, the Morro Pelado Member (*Tiarajudens* horizon) may be of lower *Tapinocephalus* zone age, whereas the Qingtoushan Formation (*Biseridens* horizon) is of upper *Tapinocephalus* zone age.

1. ***Anomocephalus africanus***

**Where:** Near Williston, Northern Cape Province, South Africa [26].

**When:** According to Day [14], *A. africanus* was recovered from a horizon equivalent to the upper Swaerskraal Member or lower Moordenaars Member of the Abrahamskraal Formation, upper *Tapinocephalus* zone (see Fig. 2).

**Phylogenetic relation:** Phylogenetic analysis indicates that *A. africanus* is a basal anomodont and sister taxon to *T. eccentricus* [20, 21].

1. ***Galechirus scholtzi***

**Where:** Various localities of South Africa [14, and references therein].

**When:** *Galechirus* has been recovered from the upper Moordenaars Member of the Abrahamskraal Formation [14, 16], upper *Tapinocephalus* zone (see Fig. 2).

**Phylogenetic relation:** *Galechirus* is a basal anomodont and is closely related to *Galepus* [20, 21].

1. ***Galeops whaitsi***

**Where:** Various localities of South Africa [14, and references therein].

**When:** The definite range of *Galeops* is restricted to the upper Moordenaars Member and the Karelskraal Member of the Abrahamskraal Formation [14], upper *Tapinocephalus* zone (see Fig. 2).

**Phylogenetic relation:** *Galeops* is a basal anomodont and is closely related to *Galepus* and *Galechirus* [20, 21].

1. ***Colobodectes cluveri***

**Where:** Farm Vleikraal in Williston District, Northern Cape Province, South Africa [27].

**When:** According to Day [14], *C. cluveri* has been recovered from the upper *Tapinocephalus* zone and possibly from the lower *Tapinocephalus* zone (see Fig. 2).

**Phylogenetic relation:** *C. cluveri* was placed phylogenetically between “*Eodicynodon*” *oelofseni* and *Eodicynodon oosthuizeni* [20, 21].

1. ***Diictodon feliceps***

**Where:** Various localities of South Africa [14, and references therein].

**When:** According to Day [14], the first certain appearance of *Diictodon* can placed at the base of the Moordenaars Member, ~2150–2200 m above the base of the Abrahamskraal Formation at its thickest point, upper *Tapinocephalus* zone (see Fig. 2).

**Phylogenetic relation:** *Diictodon* is a basal anomodont genus closely related to *Eosimops*, *Prosictodon* and *Robertia* [28].

1. ***Emydops arctatus***

**Where:** Various localities of South Africa [14, and references therein].

**When:** From the upper *Tapinocephalus* to *Tropidostoma* zones [14, 16].

**Phylogenetic relation:** *E. arctatus* is a therochelonian anomodont member of the family Emydopoidea [21].

1. ***Eosimops newtoni***

**Where:** Various localities of South Africa [14, and references therein].

**When:** According to Day [14], *Eosimops* has a stratigraphic range that extends from the mid-Swaerskraal Member of the Abrahamskraal Formation at its thickest point to the lower Poortjie Member of the Teekloof Formation, upper *Tapinocephalus* to mid-*Pristerognathus* zone (see Fig. 2).

**Phylogenetic relation:** *E. newtoni* is a basal anomodont closely related to *Diictodon*, *Prosictodon* and *Robertia* [28].

1. ***Pristerodon mackayi***

**Where:** Various localities of South Africa [14, and references therein].

**When:** The stratigraphic range of *Pristerodon* *mackayi* extends from the upper *Tapinocephalus* to *Tropidostoma* zones [14, 16].

**Phylogenetic relation:** *P. mackayi* is a basal dicynodont anomodont [21].

1. ***Prosictodon dubei***

**Where:** Various localities of South Africa [14, and references therein].

**When:** According to Day [14], *Prosictodon dubeii* occurs between the base of the Moordenaars Member of the Abrahamskraal Formation and the base of the Poortjie Member, upper *Tapinocephalus* zone (see Fig. 2).

**Phylogenetic relation:** *P. dubei* is a basal anomodont closely related to *Diictodon*, *Eosimops* and *Robertia* [28].

1. ***Robertia broomiana***

**Where:** Various localities of South Africa [14, and references therein].

**When:** According to Day [14], the certain stratigraphic range of *Robertia broomiana* extends between 1950–2550 m above the base of the Abrahamskraal Formation and encompasses the Karelskraal, Moordenaars, and upper Swaerskraal Members of the Abrahamskraal Formation at its thickest point, upper *Tapinocephalus* zone (see Fig. 2).

**Phylogenetic relation:** *R. broomiana* is a basal anomodont closely related to *Diictodon*, *Eosimops* and *Prosictodon* [28].

1. ***Brachyprosopus broomi***

**Where:** Various localities of South Africa [14, 24].

**When:** According to Day [14], the overall stratigraphic range of *Brachyprosopus* extends from the lower Abrahamskraal Formation, perhaps within the *Eodicynodon* AZ, up to the Karelskraal Member of the Abrahamskraal Formation at its thickest point (see Fig. 2).

**Phylogenetic relation:** *Chelydontops altidentalis* is a junior synonym of *B. broomi* [24]. *B. broomi* is a basal dicynodont anomodont [28].

**Amniote chrone 11 (Am11)**

1. ***Galepus jouberti***

**Where:** Near Merriman railway, Northern Cape Province, South Africa [14, and references therein].

**When:** According to Day [14], *Galeops* could be as old as the *Pristerognathus* zone.

**Phylogenetic relation:** *Galepus* is a basal anomodont and is closely related to *Galechirus* [20, 21].

1. ***Suminia getmanovi***

**Where:** Various localities of Russian Federation [6] (see below).

**When:**

1. Collection PIN 2212, Kotel’nich locality, Kirov Region, Kotel’nich Sub-assemblage, *Deltavjatia vjatkensis* zone, mid-Severodvinian (see Fig. 2).
2. Collection PIN 3159, Navoloki, Vologda, Ilinskoe Subassemblage, late Severodvinian-early Wuchiapingian (see Fig. 2).
3. Collection PIN 4548, Ust’e Strel’ny locality, Orenburg, Velikoustyugskii District, Ilinskoe Subassemblage, late Severodvinian-early Wuchiapingian (see Fig. 2).

**Phylogenetic relation:** *S. getmanovi* is a member of the anomodont family Venyukovioidea. *S. getmanovi* and *U. invisa* may form a clade separate from *O. netzvetajevi* [22], or *S. getmanovi* may be in a clade separate from the *U. invisa* + *O. netzvetajevi* [21] clade. Here, the first interpretation is supported.

1. ***Diictodon sp.***

**Where:** Various localities of South Africa. The most common dicynodont genus in South Africa [14, and references therein].

**When:** According to Day [14], *Diictodon* is from the *Pristerognathus*, *Tropidostoma*, and *Cistecephalus* zones and survives until the end of the Permian.

**Phylogenetic relation:** *Diictodon* is a basal anomodont genus closely related to *Eosimops*, *Prosictodon* and *Robertia* [28].

1. ***Endothiodon bathystoma***

**Where:** Various localities of South Africa [14, and references therein].

**When:** According to Day [14] and Day et al. [16], *Endothiodon* is from the *Pristerognathus*, *Tropidostoma*, and *Cistecephalus* zones.

**Phylogenetic relation:** *Endothiodon* is a dicynodont anomodont genus closely related to *Niassodon* [21].

1. ***Dicynodontoides recurvidens***

**Where:** Various localities of South Africa [14, and references therein].

**When:** According to Day et al. [16], the lowest stratigraphic occurrence of *Dicynodontoides* is from the upper *Pristerognathus*-*Tropidostoma* zones. Sidor et al. [29] show that it crosses the *Dicynodon* zone as well.

**Phylogenetic relation:** *Dicynodontoides* is a dicynodont anomodont member of Therochelonia [20].

1. ***Pristerodon mackayi***

**Where:** Various localities of South Africa [14, and references therein].

**When:** The stratigraphic range of *Pristerodon* *mackayi* extends from the upper *Tapinocephalus* to the *Tropidostoma* zones [14, 16].

**Phylogenetic relation:** *P. mackayi* is a dicynodont anomodont [21].

1. ***Rastodon procurvidens***

**Where:** Boqueirão farm site of the Rio do Rasto Formation, Paraná Basin, Brazil [30].

**When:** Rio do Rasto Formation [30]. Although, the age of this formation is not well-constrained, Lucas [4] considered it to be of Gamkan to earliest Hoedemakeran age, which is equivalent to the *Tapinocephalus* to early *Tropidostoma* zone [4] (see Fig. 2).

**Phylogenetic relation:** *Rastodon procurvidens* is a dicynodont anomodont and basal member of Bidentalia [30].

1. ***Tiarajudens eccentricus***

**Where:** Barro Alto, Rio Grande do Sul, Brasil [31].

**When:** Morro Pelado Member of the Rio do Rasto Formation [31]. Althoug the age of this formation is not well-constrained, Lucas [4] considered it to be of Gamkan to earliest Hoedemakeran age, which is equivalent to the *Tapinocephalus* to early *Tropidostoma* zone (see Fig. 2).

**Phylogenetic relation:** Phylogenetic analysis indicates that *T. eccentricus* is an anomodont and sister taxon to *A. africanus* [20, 21].

1. ***Idelesaurus tataricus***

**Where:** Semin Ovrag locality, Tatarstan, Russian Federation [6].

**When:** Collection PIN 156, Ilinskoe Faunal Subassemblage [6].

**Phylogenetic relation:** *Idelesaurus tataricus* is a dicynodont anomodont and basal member of Bidentalia [20].

1. ***Oudenodon sp.***

**Where:** Various localities of the Western Pangaea [32].

**When:** The stratigraphic range of *Oudenodon* spans from the upper *Tropidostoma* to the upper *Dicynodon* zones [29].

**Phylogenetic relation:** *Oudenodon* is a dicynodont anomodont, a basal member of Bidentalia, and is closely related to *Tropidostoma* [20].

1. ***Tropidostoma dubium***

**Where:** Various localities of South Africa [33].

**When:** The stratigraphic range of *Tropidostoma dubium* spans the *Tropidostoma* zone [12].

**Phylogenetic relation:** *Tropidostoma* is a dicynodont anomodont, abasal member of Bidentalia, and is closely related to *Oudenodon* [20].

1. ***Australobarbarus kotelnitshi***

**Where:** Port Kotelnich locality, Kirov Region, Russian Federation [6].

**When:** Collection PIN 4678, Kotelnich Faunal Subassemblage [6], equivalent to the *Pristerognathus* zone (see Fig. 2).

**Phylogenetic relation:** *A. kotelnitshi* is a dicynodont anomodont, a basal member of Bidentalia, and is closely related to *Oudenodon* and *Tropidostoma* [20].

1. ***Australobarbarus platycephalus***

**Where:** Port Kotelnich locality, Kirov Region, Russian Federation [6].

**When:** Collection PIN 4678, Kotelnich Faunal Subassemblage [6], equivalent to the *Pristerognathus* zone (see Fig. 2).

**Phylogenetic relation:** *A. platycephalus* is a dicynodont anomodont, a basal member of Bidentalia, and is closely related to *Oudenodon* and *Tropidostoma* [20].

1. ***Bulbasaurus phylloxyron***

**Where:** Driekoppe, Vredelus, Fraserburg, Western Cape Province, South Africa [28].

**When:** Hoedemaker Member of the Teekloof Formation, *Tropidostoma* zone [28].

**Phylogenetic relation:** *Bulbasaurus phylloxyron* is a member of the family Geikiidae [28].

1. ***Rhachiocephalus magnus***

**Where:** Ruhuhu Basin of south-west Tanzania and South Africa [34].

**When:** *Tropidostoma* zone [16] and *Cistecephalus* zone [34].

**Phylogenetic relation:** *Rhachiocephalus magnus* is a cryptodont anomodont [28].

1. ***Sauroscaptor tharavati***

**Where:** Andhra Pradesh, India [35].

**When:** *Tropidostoma*-*Cistecephalus* zone [35].

**Phylogenetic relation:** *Sauroscaptor tharavati* is a therochelonian anomodont member of the family Emydopoidea [28].

**Amniote chrone 12 (Am12)**

1. ***Diictodon sp.***

**Where:** Various localities of South Africa [14, and references therein].

**When:** *Diictodon* is from the *Pristerognathus*, *Tropidostoma*, and *Cistecephalus* zones and survived until the end of the Permian [14].

**Phylogenetic relation:** *Diictodon* is a dicynodont anomodont [21].

1. ***Endothiodon bathystoma***

**Where:** Various localities of South Africa [14, and references therein].

**When:** *Endothiodon* is from the *Pristerognathus*, *Tropidostoma*, and *Cistecephalus* zones [16].

**Phylogenetic relation:** Although three *Endothiodon* species are currently recognized, their differences exclusively in size very probably reflect an ontogenetic series and only one species, *E.* *bathystoma* is valid [14, and references therein].

1. ***Emydops oweni***

**Where:** Various localities of South Africa [36].

**When:** *Cistecephalus* zone [36].

**Phylogenetic relation:** *E. oweni* is the second valid species of *Emydops* [36].

1. ***Digalodon rubidgei***

**Where:** Central Karoo Basin, near the junction between the Western, Eastern, and Northern Cape of South Africa [37].

**When:** Upper *Cistecephalus* zone and *Dicynodon* zone [37], lower *Daptocephalus* zone of Viglietti et al. [38].

**Phylogenetic relation:** *Digalodon rubidgei* is a therochelonian anomodont member of the family Emydopoidea [20, 28].

1. ***Cistecephalus microrhinus***

**Where:** Various localities of South Africa [39].

**When:** *Cistecephalus zone* [12].

**Phylogenetic relation:** *Cistecephalus microrhinus* is a therochelonian anomodont member of the family Emydopoidea [20, 28].

1. ***Cistecephaloides boonstrai***

**Where:** Ely Cottage, near Alice, Cape Province, South Africa [40].

**When:** Upper *Cistecephalus zone* [40; see also 41, 42), possibly within the lower *Daptocephalus* zone [38].

**Phylogenetic relation:** *Cistecephaloides boonstrai* is a therochelonian anomodont member of the family Emydopoidea [20, 28].

1. ***Kawingasaurus fossilis***

**Where:** Near the Village of Kingori, Songea District, Tanzania, Tanganyika, East Africa [43].

**When:** The Usili Formation (Songea Group) of southern Tanzania [29] is equivalent to the former upper *Cistecephalus* – lowermost *Dicynodon* zone [44] and coincides with the recently defined lower *Daptocephalus* zone [38].

**Phylogenetic relation:** *Kawingasaurus fossilis* is a therochelonian anomodont member of the family Emydopoidea [20, 28].

1. ***Niassodon mfumukasi***

**Where:** Tulo, Lago District, Niassa Province, northern Mozambique [20].

**When:** *Cistecephalus* zone [20].

**Phylogenetic relation:** *N. mfumukasi* is a therochelonian anomodont member of the family Emydopoidea closely related to *Endothiodon* [20, 28].

1. ***Dicynodontoides recurvidens***

**Where:** Various localities of South Africa [14, and references therein].

**When:** According to Day et al. [16], the lowest stratigraphic occurrence of *Dicynodontoides* is the upper *Pristerognathus*-*Tropidostoma* zone. Sidor et al. [29] showed that it crossed the *Dicynodon* zone as well.

**Phylogenetic relation:** *Dicynodontoides* is a therochelonian anomodont member of the family Emydopoidea [20, 28].

1. ***Odontocyclops whaitsi***

**Where:** Various localities of South Africa and Zambia [45].

**When:** *Cistecephalus*–*Dicynodon* zone [45].

**Phylogenetic relation:** *Odontocyclops whaitsi* is a cryptodont anomodont member of the family Oudenodontidae [28].

1. ***Aulacephalodon bainii***

**Where:** Various localities of South Africa [46].

**When:** *Cistecephalus*–*Dicynodon* zone [46].

**Phylogenetic relation:** *Aulacephalodon bainii* is a cryptodont anomodont member of the family Geikiidae [28].

1. ***Pelanomodon moschops***

**Where:** Central Karoo Basin, near the junction between the Western, Eastern, and Northern Cape of South Africa [37, 47].

**When:** Upper *Cistecephalus* zone and *Dicynodon* zone [37]), lower *Daptocephalus* zone [38].

**Phylogenetic relation:** *Pelanomodon moschops* is a cryptodont anomodont member of the family Geikiidae [28].

1. ***Geikia locusticeps***

**Where:** Ruhuhu Basin of southwest Tanzania [48].

**When:** Usili Formation of Kingori [48], which is equivalent to the former upper *Cistecephalus* – lowermost *Dicynodon* zone [44] and coincides with the recently defined lower *Daptocephalus* zone [38].

**Phylogenetic relation:** *Geikia locusticeps* is a cryptodont anomodont member of the family Geikiidae [28].

1. ***Rhachiocephalus magnus***

**Where:** Ruhuhu Basin of southwest Tanzania and South Africa [49].

**When:** *Tropidostoma* zone [16] and *Cistecephalus* zone [49].

**Phylogenetic relation:** *Rhachiocephalus magnus* is a cryptodont anomodont [28].

1. ***Rhachiocephalus behemoth***

**Where:** Ruhuhu Basin of southwest Tanzania [50].

**When:** Usili Formation of Kingori [48], which is equivalent to the former upper *Cistecephalus* – lowermost *Dicynodon* zone [44] and coincides with the recently defined lower *Daptocephalus* zone [38].

**Phylogenetic relation:** *Rhachiocephalus* is a cryptodont anomodont closely related to *Kitchinganomodon* and a member of the family Rhachiocephalidae [28].

1. ***Kitchinganomodon crassus***

**Where:** South Africa and Zambia [51].

**When:** Upper *Cistecephalus* zone and *Dicynodon* zone [37], lower *Daptocephalus* zone [38].

**Phylogenetic relation:** *Kitchinganomodon* is a cryptodont anomodont closely related to *Rhachiocephalus* and a member of the family Rhachiocephalidae [28].

1. ***Daqingshanodon limbus***

**Where:** Shiguai, Nei Monggol, China [21, and references therein].

**When:** *Daptocephalus* zone [21].

**Phylogenetic relation:** *Daqingshanodon limbus* is a cryptodont anomodont [28].

1. ***Keyseria benjamini***

**Where:** Brookfield, Murraysburg, South Africa [21].

**When:** Upper *Cistecephalus* zone and *Dicynodon* zone [37], lower *Daptocephalus* zone [38].

**Phylogenetic relation:** *Keyseria benjamini* is a cryptodont anomodont [28].

1. ***Syops vanhoepeni***

**Where:** Luangwa Basin, Zambia [21, 42].

**When:** *Cistecephalus* zone [21, 42].

**Phylogenetic relation:** *Syops vanhoepeni* is a cryptodont anomodont member of the family Rhachiocephalidae [28].

1. ***Dicynodon huenei***

**Where:** Luangwa Basin, Zambia and Tanzania [21, 42].

**When:** *Cistecephalus* zone [21, 42], probably within the lower *Daptocephalus* zone (Usili Formation age) [38].

**Phylogenetic relation:** *Dicynodon huenei* is an anomodont, member of the group Dicynodontoidea [28].

1. ***Dicynodon laceticeps***

**Where:** South Africa [21].

**When:** *Cistecephalus* and lower *Daptocephalus* zone [38].

**Phylogenetic relation:** *Dicynodon laceticeps* is an anomodont, member of the group Dicynodontoidea [28].

1. ***Dinanomodon gilli***

**Where:** South Africa [21].

**When:** *Cistecephalus* and *Dicynodon* zones [21], lower and upper *Daptocephalus* zones [38].

**Phylogenetic relation:** *Dinanomodon gilli* is an anomodont closely related to *Daptocephalus* and *Peramodon* within Dicynodontoidea [28].

1. ***Basilodon woodwardi***

**Where:** Various localities of South Africa [21].

**When:** Upper *Cistecephalus* zone and *Dicynodon* zone [37], lower *Daptocephalus* zone [38].

**Phylogenetic relation:** *Basilodon woodwardi* is closely related to TSK 2 within Dicynodontoidea and [28].

1. **TSK 2 (*Lystrosaurus cf. L. curvatus*)**

**Where:** North side of Munyamadzi River, Luangwa Valley, Zambia [42].

**When:** Madumabisa Mudstone, *Cistecephalus* to lower *Daptocephalus* zone [38].

**Phylogenetic relation:** TSK specimens determined to be closely related to *Basilodon* within Dicynodontoidea [28].

1. ***Sintocephalus alticeps***

**Where:** One mile west of Oudeberg, Graaff-Reinet, South Africa [21].

**When:** *Cistecephalus* zone [21].

**Phylogenetic relation:** *Sintocephalus alticeps* is an anomodont closely related to TSK 2 and *Basilodon* within Dicynodontoidea [28].

1. ***Vivaxosaurus trautscholdi***

**Where:** Berezhane locality, Kirov Region, Russian Federation [6, 21].

**When:** Collection PIN 1536, Sokolki Faunal Subassemblage [6, 52].

**Phylogenetic relation:** *Vivaxosaurus trautscholdi* is an anomodont closely related to *Delectosaurus* within Dicynodontoidea [28].

1. ***Peramodon amalitzkii***

**Where:** Sokolki locality, Arkhangelsk Region, Russian Federation [6: “*Dicynodon” amalitzkii*).

**When:** Collection PIN 2005, Sokolki Faunal Subassemblage [6, 52].

**Phylogenetic relation:** *Peramodon* (*Dicynodon*) *amalitzkii* is an anomodont closely related to *Daptocephalus* and *Dinanomodon* within Dicynodontoidea [28].

1. ***Delectosaurus arefjevi***

**Where:** Voskresenskoe-2B locality, Nizhni Novgorod Region, Russian Federation [6, 21].

**When:** Collection PIN 4644, Sokolki Faunal Subassemblage [6, 52].

**Phylogenetic relation:** *Delectosaurus arefjevi* is an anomodont closely related to *Vivaxosaurus* within Dicynodontoidea [28].

1. ***Euptychognathus bathyrhynchus***

**Where:** South Africa and Tanzania [21].

**When:** *Cistecephalus* zone [21].

**Phylogenetic relation:** *Euptychognathus bathyrhynchus* is closely related to the lystrosaurids and *Kwazulusaurus shakai* within Dicynodontoidea [28].

1. ***Kwazulusaurus shakai***

**Where:** Stoffelton, Polela District, KwaZulu-Natal, South Africa [53].

**When:** *Dicynodon* zone [53], lower *Daptocephalus* zone.

**Phylogenetic relation:** *Kwazulusaurus shakai* is closely related to the lystrosaurids and *Euptychognathus bathyrhynchus* within Dicynodontoidea [28].

1. ***Elph borealis***

**Where:** Sokolki locality, Arkhangelsk Region, Russian Federation [6].

**When:** Collection PIN 2005, Sokolki Faunal Subassemblage [6, 52].

**Phylogenetic relation:** *Elph borealis* is closely related to *Katumbia* within Dicynodontoidea [28].

1. ***Interpresosaurus blomi***

**Where:** Voskresenskoe-2A locality, Nizhni Novgorod Region, Russian Federation [54].

**When:** Sokolki Faunal Subassemblage [6, 52].

**Phylogenetic relation:** *Interpresosaurus blomi* is closely related to *Elph* and *Katumbia* within Dicynodontoidea [28].

1. ***Katumbia parringtoni***

**Where:** Luangwa Basin, Zambia and Ruhuhu Basin, Tanzania [42].

**When:** Upper Madumabisa Mudstone (Zambia) and Usili Formation [42], *Cistecephalus* zone to lower *Daptocephalus* zone [38].

**Phylogenetic relation:** *Katumbia parringtoni* is closely related to *Elph* within Dicynodontoidea [28].

1. ***Daptocephalus leoniceps***

**Where:** South Africa [38].

**When:** Lower and upper *Daptocephalus* zone [38].

**Phylogenetic relation:** *Daptocephalus leoniceps* is an anomodont closely related to *Peramodon* and *Dinanomodon* within Dicynodontoidea [28].

**Younger taxa**

Although they are closely related phylogenetically to the taxa listed above, the following taxa are younger than the lower *Daptocephalus* zone, thus they are not further considered here:

*Kombuisia frerensis, Lystrosaurus maccaigi*, *Geikia elginensis*, *Gordonia traquairi, Angonisaurus cruickshanki, Jimusaria sinkianensis, Turfanodon bogdaensis, Myosaurus gracilis.*

**Biarmosuchia**

**Amniote chrone 9 (Am9)**

1. ***Biarmosuchus tener***

**Where:** Ezhovo locality, Permian Region, Russian Federation [6].

**When:** Collection PIN 1758, Ocher Faunal Subassemblage, Upper Kazanian [6] (see Fig. 2).

**Phylogenetic relation:** The genus *Biarmosuchus* is the most basal and the oldest member of the family Biarmosuchidae [55, 56].

1. ***Biarmosuchus tchudinovi***

**Where:** Sokol locality, Udmurtia, Zav’yalovskii District, Russian Federation [6].

**When:** Collection PIN 4309, Ocher Faunal Subassemblage, Upper Kazanian [6] (see Fig. 2).

**Phylogenetic relation:** The genus *Biarmosuchus* is the most basal and the oldest member of the family Biarmosuchidae [55, 56]. *B. tchudinovi* is currently only represented by maxillary bones. Ivakhnenko [6] stated that “new finds will result in the establishment of a separate genus or even family, since the relatively small size of this animal is in contrast with the biomorph of the family considered”. As such, *B. tchudinovi* is not further considered here.

1. ***Pachydectes elsi***

**Where:** Farm Môrester, Jansenville, Eastern Cape Province, South Africa [57].

**When:** Day and colleagues [55] determined that *P. elsi* is from the lower *Tapinocephalus* zone. However, according to Day [14], *P. elsi* has been recovered at 800-1000 m above the base of the Abrahamsrkaal Formation at its thickest point, uppermost Combrinkskraal to lower Leeuvlei Members, Abrahamskraal Formation, which corresponds to the *Eodicynodon* zone (see Fig. 2). If so, according to the current approach, if *Biarmosuchus tchudinovi* is a valid biarmosuchian taxon, then *Pachydectes elsi* and *B. tchudinovi* belong to the same lineage.

**Phylogenetic relation:** Basal biarmosuchian or burnetiamorph [55, 56].

**Amniote chrone 10 (Am10)**

1. ***Bulacephalus jacksoni***

**Where:** Middelvlei Farm, Western Cape, South Africa [58].

**When:** According to Day [14], *B. jucksoni* has been recovered at a depth of1650-1700 m in the Beaufort Group sequence, uppermost Koornplaats Member, Abrahamskraal Formation, which corresponds to the lowermost upper *Tapinocephalus* zone (see Fig. 2).

**Phylogenetic relation:** Basal biarmosuchian or burnetiamorph [55, 56].

1. **TM 4305 (unnamed burnetiamorph specimen)**

**Where:** Unknown locality, South Africa [56].

**When:** *Tapinocephalus* zone [56].

**Phylogenetic relation:** A purely preserved specimen, which was placed within the burnetiamorphs in the phylogenetic analysis of Kammerer [56].

1. ***Hipposaurus boonstrai***

**Where:** South Africa. According to Day [14]:

1. Farm Rietfontein, in the Beaufort West district of the central Koup.
2. Farm Seekoegat, Prince Albert district.
3. Farm Klein Koedoeskop, Beaufort West district.
4. Farm Rietfontein, Prince Albert district.

**When:** According to Day [14], the stratigraphic range of *H. boonstrai* can be constrained to the interval 2200–2500 m above the base of the Abrahamsrkaal Formation at its thickest point, which is equivalent to the Moordenaars Member and lower Karelskraal Member. Thus, *H. boonstrai* belongs to the upper *Tapinocephalus* zone (see Fig. 2).

**Phylogenetic relation:** Basal biarmosuchian [55, 56].

**Amniote chrone 11 (Am11)**

1. ***Lobalopex mordax***

**Where:** Quaggas Fontein 250, Victoria West District, South Africa [59].

**When:** Top of the Teekloof Formation (uppermost Pisterognathus-lowermost *Tropidostoma* zone) [14, 59, 60].

**Phylogenetic relation:** *L. mordax* is a burnetiamorph biarmosuchian placed outside of the family Burnetidae [55, 56].

1. ***Proburnetia viatkensis***

**Where:** Agafonovo locality, Kirov Region, Russian Federation [6].

**When:** Collection PIN 2416, Ilinskoe Faunal Subassemblage [6], which is equivalent to the *Tropidostoma* zone (see Fig. 2).

**Phylogenetic relation:** *P. viatkensis* is a burnetiamorph biarmosuchian and basal member of the family Burnetidae [55, 56].

1. ***Niuksenitia sukhonensis***

**Where:** Navoloki locality, Vologda Region, Russian Federation [6].

**When:** Collection PIN 3159, Ilinskoe Faunal Subassemblage [6], which is equivalent to the *Tropidostoma* zone (see Fig. 2).

**Phylogenetic relation:** *N. sukhonensis* is a burnetiamorph biarmosuchian placed outside of [55] or within the family Burnetidae [56].

1. **BP/1/7098 (unnamed biarmosuchian specimen)**

**Where:** Farm Springfontein, Beaufort West district, South Africa [55].

**When:** Middle *Pristerognathus* zone [55].

**Phylogenetic relation:** BP/1/7098 is a currently unnamed burnetiamorph biarmosuchian specimen, which is closely related to *N. sukhonensis* [55].

1. ***Lophorhinus willodenensis***

**Where:** Teekloof Pass, Beaufort West district, Western Cape Province, South Africa [61].

**When:** Middle Teekloof Formation, middle *Tropidostoma* zone [60, 61].

**Phylogenetic relation:** *L. willodenensis* is a burnetiamorph biarmosuchian placed outside of the family Burnetidae [55, 56].

1. ***Ustia atra***

**Where:** Ust’e Strel’ny locality, Vologda Region, Russian Federation [6].

**When:** Collection PIN 4548, Ilinskoe Faunal Subassemblage [6], which is equivalent to the *Tropidostoma* zone (see Fig. 2).

**Phylogenetic relation:** *U. atra* is a poorly preserved biarmosuchian that has never been considered in a comprehensive phylogenetic study. Thus, its exact position in the biarmosuchian phylogenetic tree is unknown.

1. **BP/1/7098 (unnamed biarmosuchian specimen)**

**Where:** Farm Springfontein, Beaufort West district, South Africa [55].

**When:** equivalent to the *Tropidostoma* zone (see Fig. 2).

**Phylogenetic relation:** BP/1/7098 is an unnamed burnetiamorph biarmosuchian specimen, which is closely related to the Russian taxon *N. sukhonensis* [55].

1. **NHMUK R871 (unnamed burnetiamorph specimen)**

**Where:** Tafelberg, Beaufort West, South Africa [56].

**When:** *Tropidostoma* zone [56].

**Phylogenetic relation:** A purely preserved specimen, which was placed within the burnetiamorph biarmosuchians in the phylogenetic analysis of Kammerer [56].

1. ***Lycaenodon longiceps***

**Where:** Biesjespoort Station, Victoria West District, Northern Cape Province, South Africa [55].

**When:** Upper *Tropidostoma* zone [55].

**Phylogenetic relation:** A biarmosuchian member of the family Ictidorhinidae [55].

1. **RC20 (cf. *Lycaenodon* = *Hipposaurus rubidgei*)** [62]

**Where:** Wellwood, Graaff-Reinet District, South Africa [62, and references therein].

**When:** The RC20 specimen was considered to be from the *Cistecephalus* zone [55, 60], however, Sidor [62] consider this specimen to come from the same biozone as *L. longiceps*. Therefore, it can be considered to also belong in the upper *Tropidostoma* zone.

**Phylogenetic relation:** A biarmosuchian member of the family Ictidorhinidae [55].

**Amniote chrone 12 (Am12)**

1. ***Paraburnetia sneeubergensis***

**Where:** Farm Walplaas, Aberdeen District, South Africa [63].

**When:** Teekloof Formation, lowermost *Cistecephalus* Assemblage Zone [60, 63].

**Phylogenetic relation:** *P. sneeubergensis* is a burnetiamorph biarmosuchian and member of the family Burnetidae [55, 56].

1. ***Burnetia mirabilis***

**Where:** Farm Water Krantz, Eastern Cape, South Africa [64].

**When:** Former *Dicynodon* zone [60, 64], which is equivalent to the new upper *Daptocephalus* zone [38].

**Phylogenetic relation:** *B. mirabilis* is a burnetiamorph biarmosuchian and member of the family Burnetidae [55, 56].

1. ***Lende chiweta***

**Where:** Chiweta, Northern Region, Malawi [65].

**When:** Fossils 'B1', Lower Bone Bed, Chiweta Beds, equivalent to the *Cistecephalus* zone [65].

**Phylogenetic relation:** *L. chiweta* is a burnetiamorph biarmosuchian and member of the family Burnetidae [55, 56].

1. ***Lemurosaurus pricei***

**Where:** Farm Dorsfontein, Graaff-Reinet District, Eastern Cape Province, South Africa [66].

**When:** *Cistecephalus* zone [60, 66].

**Phylogenetic relation:** *L. pricei* is a burnetiamorph biarmosuchian placed outside of the family Burnetidae [55, 56].

1. ***Ictidorhinus martinsi***

**Where:** Wilgerbosch, New Bethesda, Eastern Cape, South Africa [67].

**When:** Upper *Daptocephalus* zone [55].

**Phylogenetic relation:** A biarmosuchian member of the family Ictidorhinidae [55].

1. ***Herpetoskylax hopsoni***

**Where:** Matjiesfontein, Western Cape, South Africa [68].

**When:** *Cistecephalus* zone [55, 60].

**Phylogenetic relation:** A biarmosuchian member of the family Ictidorhinidae [55].

**Gorgonopsia**

**Amniote chrone 10 (Am10)**

1. ***Eriphostoma microdon***

**Where:** Western and Northern Cape, South Africa [69].

**When:** Upper *Tapinocephalus* zone [69].

**Phylogenetic relation:** Basal gorgonopsian [70].

1. ***Arctognathus cf. curvimola***

**Where:** Western Cape, South Africa [69].

**When:** *Tapinocephalus* zone [69].

**Phylogenetic relation:** A possible basal gorgonopsian, which currently can only be referred to as Gorgonopsia *incertae sedis* [69].

**Amniote chrone 11 (Am11)**

1. ***Nochnitsa geminidens***

**Where:** North bank of the Vyatka River, Russian Federation [70].

**When:** Kotelnich locality, Vanyushonki Member (lowest), Kotelnich Subassemblage [70], equivalent to the *Pristerognathus* zone (see Fig. 2).

**Phylogenetic relation:** Basal gorgonopsian [70].

1. ***Viatkogorgon ivakhnenkoi***

**Where:** Kotel’nichskii District, Kirov Region, Russian Federation [70].

**When:** Kotelnich locality, Kotelnich Subassemblage [70], equivalent to the upper *Pristerognathus* zone (see Fig. 2).

**Phylogenetic relation:** Basal gorgonopsian [70].

1. ***Suchogorgon golubevi***

**Where:** Ust’e Strel’ny Locality, Vologda Region, Russian Federation [6].

**When:** *Proelginia permiana* Zone, Ilinskoe Faunal Subassemblage [6], equivalent to the *Tropidostoma* zone (see Fig. 2).

**Phylogenetic relation:** Gorgonopsian [70].

1. ***Sauroctonus progressus***

**Where:** Semin Ovrag locality, Tatarstan, Russian Federation [71].

**When:** *Proelginia permiana* Zone, Ilinskoe Faunal Subassemblage [71], equivalent to the *Tropidostoma* zone (see Fig. 2).

**Phylogenetic relation:** Gorgonopsian [70] closely related to the African species *Sauroctonus parringtoni* [72].

1. ***Gorgonops torvus***

**Where:** South Africa [73]:

1. Mildenhalls near Fort Beaufort, Western Cape Province.
2. Beaufort West, Cape Colony, Western Cape Province.
3. Farm Driehoeksfontein in the Murraysburg District, Western Cape Province.
4. Driehoeksfontein, Murraysburg District, Western Cape Province.
5. Rooipoort, Murraysburg District, Western Cape Province.

**When:** According to ref. [73]:

1. Hoedemaker member, Middle Teekloof Formation, *Tropidostoma* zone (see Fig. 2).
2. Hoedemaker member, Middle Teekloof Formation, *Tropidostoma* zone (see Fig. 2).
3. Hoedemaker member, Middle Teekloof Formation, *Tropidostoma* zone (see Fig. 2).
4. *Cistecephalus* zone.
5. *Cistecephalus* zone.

Given that the uppermost *Tropidostoma* zone penetrates the *Cistecephalus* zone (see Fig. 2), this taxon can be considered to fall within the *Tropidostoma* zone.

**Phylogenetic relation:** Gorgonopsian [70].

1. ***Cynariops robustus***

**Where:** Northern and Western Cape Province, South Africa [74].

**When:** *Tropidostoma* zone [74].

**Phylogenetic relation:** Gorgonopsian [74].

1. ***“Aelurognathus tigriceps”***

**Where:** South Africa [75] .

**When:** *Tropidostoma* zone [16, 76].

**Phylogenetic relation:** Gorgonopsian [74, 75]. Member of the gorgonopsian subfamily Rubidgeinae [75].

**Amniote chrone 12 (Am12)**

1. ***Pravoslavlevia parva***

**Where:** Collection PIN 2005 (Sokolki locality), Russian Federation [6].

**When:** Sokolki Faunal Subassemblage, *Scutosaurus karpinskii* zone [6] (see Fig. 2).

**Phylogenetic relation:** A gorgonopsian of the “Russian” clade, which is closely related to the genus *Inostrancevia* [70].

1. ***Inostrancevia sp.***

**Where:** Various localities of Russian Federation [6] (see below):

1. Collection PIN 2005 (Sokolki locality).
2. Collection PIN 2353, 2356 (Zavrazh’e locality).
3. Collection PIN 2896 (Blumental-3 locality).

**When:** Sokolki Faunal Subassemblage, *Scutosaurus karpinskii* zone [6] (see Fig. 2).

**Phylogenetic relation:** A gorgonopsian of the “Russian” clade, which is closely related to *P. parva* [70].

1. ***Sauroctonus parringtoni (= ?Aelurognathus* *parringtoni)*** [72]

**Where:** Usili-Berges, Ruhuhu Valley, Tanganyika, East Africa [72, and references therein].

**When:** Usili Formation (Songea Group) of southern Tanzania [29], which is equivalent to the former upper *Cistecephalus* – lowermost *Dicynodon* zone [44] and coincides with the recently defined lower *Daptocephalus* zone [38].

**Phylogenetic relation:** *A. parringtoni* was renamed to *S. parringtoni* by Gebauer [72]. It is closely related to the Russian gorgonopsian species *Sauroctonus progressus* [72].

1. ***Arctops willistoni***

**Where:** Various localities of South Africa (see below) [77].

**When:** *A. willistoni* has been accepted to be from the *Cistecephalus* zone [77], however, there is little evidence to support this age. The ages of the specimens currently assigned to *A. willistoni* [77] are as follows:

1. Locality Howse Poort, near Fort Beaufort, Western Cape Province. Holotype of *A. willistoni* (NHMUK R4049): uncertain precise stratigraphic position [77].
2. Locality Leeufontein, Murraysburg District, Western Cape Province. Holotype of *A. kitchingi* (BP/1/575 = B.P.I. 265): Oudeberg Member, Teekloof Formation, *Cistecephalus* zone [78]. According to Viglietti et al. [38], the upper half of the Oudeberg Member falls within the older upper *Cistecephalus* zone, which has been proposed by Viglietti et al. [38] to be renamed as the lower *Daptocephalus* zone. Therefore, the exact stratigraphic position of the *A. kitchingi* holotype is unknown. This speciemen falls within the *Cistecephalus* zone or the lower *Daptocephalus* zone [38].
3. Locality Aasvogelkrans, Murraysburg District, Western Cape Province. Holotype of *A. watsoni* (BP/1/698 = B.P.I. 263): Oudeberg Member, Teekloof Formation, *Dicynodon*, *Daptocephalus,* and upper *Cistecephalus* zones [78], thus the lower *Daptocephalus* zone [38].
4. Locality Leeufontein, Murraysburg District, Western Cape Province. Holotype of *Lycaenops* *angusticeps (*UCMP 42701): *Dicynodon*, *Daptocephalus, and upper Cistecephalus zones* [79], thus the lower *Daptocephalus* zone [38].

The following previously nondescribed specimens were provisionally determined to be from the *Cistecephalus* zone by Kammerer [77] in the absence of more detailed stratigraphic evidence:

1. Unknown locality and horizon, specimen CGS 319 [77].
2. Farm Lossekop, specimen CGS JW 83-34 [77].
3. Farm Matjeskloof, north of Beaufort West, specimen CGS RMS 63 [77].
4. Farm Bastardspoort, north-west of Beaufort West, specimen CGS S 125 [77].

Consequently, most of the currently available data suggests that *A. willistoni* falls within the upper *Cistecephalus* zone, which is equivalent to the lower *Daptocephalus* zone [38].

**Phylogenetic relation:** A gorgonopsian closely related to *Smilesaurus ferox*. The two form a clade outside of the subfamily Rubidgeinae [70, 74, 77].

1. ***Smilesaurus ferox***

**Where:** Graaff-Reinet, Richmond, Pearston, Beaufort West, Fraserburg, Somerset East, Colesberg, South Africa [75].

**When:** *Tropidostoma*?, *Cistecephalus* zone [75, 80].

**Phylogenetic relation:** A gorgonopsian closely related to *Arctops willistoni*. The two form a clade outside of the subfamily Rubidgeinae [70, 74, 77].

1. ***Lycaenops ornatus***

**Where:** Eastern, Western, and Northern Cape, Graaff-Reinet, South Africa [81].

**When:** *Cistecephalus* zone [81].

**Phylogenetic relation:** Gorgonopsian [74].

1. ***Arctognathus curvimola***

**Where:** Eastern, Western, and Northern Cape, as well as Free State, South Africa [82].

**When:** *Cistecephalus–Dicynodon* zone [82].

**Phylogenetic relation:** Gorgonopsian [74].

1. ***Aelurognathus tigriceps***

**Where:** Africa:

1. Graaff-Reinet, Nieu Bethesda, Richmond, Pearston, Beaufort West, Murraysburg, Fraserburg, Somerset East, Colesberg, South Africa [75].
2. Locality 5 of the Luangwa Valley, Zambia [75].

**When:** *Cistecephalus* and *Daptocephalus* zones [74, 76]. Member of the gorgonopsian subfamily Rubidgeinae [75].

**Phylogenetic relation:** Gorgonopsian [74, 75].

1. ***Ruhuhucerberus haughtoni***

**Where:** Ruhuhu Basin, Tanzania [75].

**When:** Usili Formation (Songea Group) of southern Tanzania [29], which is equivalent to the former upper *Cistecephalus* – lowermost *Dicynodon* zone [44] and coincides with the recently defined lower *Daptocephalus* zone [38]. Member of the gorgonopsian subfamily Rubidgeinae [75].

**Phylogenetic relation:** Member of the gorgonopsian subfamily Rubidgeinae [74, 75].

1. ***Sycosaurus laticeps***

**Where:** Farm Zuurplaas, Graaff-Reinet, South Africa [75; Holotype].

**When:** The holotype of this taxon from Farm Zuurplaas is from the former upper *Cistecephalus*, *Dicynodon*, and *Daptocephalus* zones [83], which correspond to the recently defined lower *Daptocephalus* zone [38]. The ages of the other specimens in this taxon described in Kammerer [75] are unknown to the author.

**Phylogenetic relation:** Member of the gorgonopsian subfamily Rubidgeinae [75].

1. ***Sycosaurus nowaki***

**Where:** Ruhuhu Basin, Tanzania [75].

**When:** Usili Formation (Songea Group) of southern Tanzania [29], which is equivalent to the former upper *Cistecephalus* – lowermost *Dicynodon* zone [44] and coincides with the recently defined lower *Daptocephalus* zone [38].

**Phylogenetic relation:** Member of the gorgonopsian subfamily Rubidgeinae [75].

1. ***Leontosaurus vanderhorsti (=Sycosaurus vanderhorsti)*** [75]

**Where:** Graaff-Reinet, Richmond, and Murraysburg, South Africa [75].

**When:** Upper *Cistecephalus*, *Dicynodon*, and *Daptocephalus* zones, which correspond to the recently defined lower *Daptocephalus* zone [38, 83].

**Phylogenetic relation:** Member of the gorgonopsian subfamily Rubidgeinae and the clade Rubidgeini [75].

1. ***Dinogorgon rubidgei***

**Where:** According to ref. [75]:

1. Ruhuhu Basin, Tanzania.
2. Wellwood, Eastern Cape, South Africa.
3. Oudeberg, Eastern Cape, South Africa.
4. Graaff-Reinet, South Africa.

**When:**

1. Usili Formation (Songea Group) of southern Tanzania [29], which is equivalent to the former upper *Cistecephalus* – lowermost *Dicynodon* zone [44] and coincides with the recently defined lower *Daptocephalus* zone [38].
2. Wellwood, Graaff-Reinet, South Africa, *Cistecephalus* zone [84].
3. Oudeberg, *Dicynodon* zone [84].
4. Graaff-Reinet, *Daptocephalus* zone [84].

**Phylogenetic relation:** Member of the gorgonopsian subfamily Rubidgeinae and the clade Rubidgeini [75].

1. ***Rubidgea atrox***

**Where:** Various localities of South Africa (see below) [75, 85].

**When:** According to ref. [85]:

1. Dorsfontein, Graaff-Reinet (*Cistecephalus* Assemblage Zone).
2. Sondagsriviershoek (*Cistecephalus* Assemblage Zone).
3. Patrysfontein, Wellwood (*Cistecephalus* or *Daptocephalus* Assemblage Zone).
4. Doornplaas (*Cistecephalus* or *Daptocephalus* Assemblage Zone).
5. Vlakplaas, Vlakteplaas, Graaff-Reinet (*Daptocephalus* Assemblage Zone).
6. Coetzeeskraal, Murraysburg (*Daptocephalus* Assemblage Zone).
7. Soetvlei, Richmond (*Daptocephalus* Assemblage Zone).
8. Doornberg, New Bethesda (*Daptocephalus* Assemblage Zone).

The overlapping occurrence of this taxon within the *Cistecephalus*-*Daptocephalus* zones indicates that it falls within the recently defined lower *Daptocephalus* zone [38].

**Phylogenetic relation:** Member of the gorgonopsian subfamily Rubidgeinae and the clade Rubidgeini [75]. Closely related to *C. rubidgei.*

1. ***Clelandina rubidgei***

**Where:** Eastern and Western Cape Provinces, South Africa [86].

**When:** *Cistecephalus* zone [86].

**Phylogenetic relation:** Member of the gorgonopsian subfamily Rubidgeinae and the clade Rubidgeini [75]. Closely related to *R. atrox.*

**Therocephalia**

**Amniote chrone 9 (Am9)**

1. ***Porosteognathus efremovi***

**Where:** Isheevo locality, Tatarstan, Apastovskii District, Russian Federation [87].

**When:** Collection PIN 157, *Ulemosaurus* Assemblage Zone [87], Urzhumian to lowermost Severodvinian (see Fig. 2).

**Phylogenetic relation:** *P. efremovi* is a therocephalian within the family Lycosuchidae [87] and probably within Scylacosauridae [70]. *P. efremovi* is represented by fragmentary material, thus it has never been considered in a formal phylogenetic study.

1. ***Glanosuchus macrops***

**Where:** Various localities of South Africa [14, and references therein; 88].

**When:** The certain stratigraphic range of *Glanosuchus* is between 700 m above the base of the Abrahamskraal Formation at its thickest point (upper Combrinkskraal Member) to the top of the Poortjie Member of the Teekloof Formation [14, 16], upper *Tapinocephalus* to *Pristerognathus* zone (see Fig. 2).

**Phylogenetic relation:** A therocephalian within the family Scylacosauridae [70].

1. ***Ictidosaurus angusticeps***

**Where:** Various localities of South Africa [14, and references therein; 88].

**When:** The stratigraphic range of *Ictidosaurus* extends from the upper Combrinkskraal Member of the Abrahamskraal Formation at its thickest point 750–800 m above its base to the upper Karelskraal Member [14, 16].

**Phylogenetic relation:** The genus *Biarmosuchus* is the most basal and the oldest member of the family therocephalian Biarmosuchidae [55, 75].

**Amniote chrone 10 (Am10)**

1. ***Glanosuchus macrops***

**Where:** Various localities of South Africa [14, and references therein; 88].

**When:** The stratigraphic range of *Glanosuchus* is between 700 m above the base of the Abrahamskraal Formation at its thickest point (upper Combrinkskraal Member) to the top of the Poortjie Member of the Teekloof Formation [14, 16], upper *Tapinocephalus* to *Pristerognathus* zone (see Fig. 2).

**Phylogenetic relation:** A therocephalian within the family Scylacosauridae [70].

1. ***Ictidosaurus angusticeps***

**Where:** Various localities of South Africa [14, and references therein; 88].

**When:** The stratigraphic range of *Ictidosaurus* extends from the upper Combrinkskraal Member of the Abrahamskraal Formation at its thickest point 750–800 m above its base to the upper Karelskraal Member [14, 16].

**Phylogenetic relation:** A therocephalian within the family Scylacosauridae [70].

1. ***Lycosuchus vanderrieti***

**Where:** Various localities of South Africa [14, and references therein].

**When:** The stratigraphic range of *Lycosuchus* extends from a horizon 2450 m above the base of the Abrahamskraal Formation at its thickest point in the uppermost Moordenaars Member to the top of the Poortjie Member of the Teekloof Formation [14, 16], upper *Tapinocephalus* to *Pristerognathus* zone (see Fig. 2).

**Phylogenetic relation:** *Lycosuchus* and *Simorhinella* are currently the only recognized genera in the family Lycosuchidae [89].

1. ***Simorhinella baini***

**Where:** South Africa. 1) Weltevreden, Gouph, Prince Albert District, Western Cape Province. 2) Farm Rheboksfontein 74, Victoria West district, Northern Cape Province [89].

**When:** Upper *Tapinocephalus* zone [89].

**Phylogenetic relation:** *Simorhinella* and *Lycosuchus* are currently the only recognized genera in the family Lycosuchidae [89].

1. ***Pristerognathus polyodon***

**Where:** Various localities of South Africa [14, and references therein].

**When:** The stratigraphic range of *Pristerognathus* corresponds to an interval 2100–2400 m above the base of the Abrahamskraal Formation at its thickest point from the uppermost Swaerskraal Member to the upper Moordenaars Member [14, 16].

**Phylogenetic relation:** A therocephalian, member of the family Scylacosauridae [70].

1. ***Scylacosaurus sclateri***

**Where:** Various localities of South Africa [14, and references therein].

**When:** The certain stratigraphic range of *Scylacosaurus* extends from 2250 m above the base of the Abrahamskraal Formation at its thickest point in the lower Moordenaars Member up to the mid-Poortjie Member of the Teekloof Formation [14, 16].

**Phylogenetic relation:** A therocephalian, member of the family Scylacosauridae [70].

1. ***Alopecodon priscus***

**Where:** Various localities of South Africa [14, and references therein].

**When:** The occurrence of the *Alopecodon* correlated with the Moordenaars Member of the Abrahamskraal Formation [14, 16].

**Phylogenetic relation:** A therocephalian, member of the family Scylacosauridae and closely related to *Pardosuchus* [70].

1. ***Pardosuchus whaitsi***

**Where:** Various localities of South Africa [14, and references therein].

**When:** *Pardosuchus* occurs within the Moordenaars Member of the Abrahamskraal Formation at its thickest point [14, 16].

**Phylogenetic relation:** A therocephalian, member of the family Scylacosauridae and closely related to *Alopecodon* [70].

1. ***Crapartinella croucheri***

**Where:** Weltevreden, Goup, North Cape West Province, South Africa [90].

**When:** *Tapinocephalus* zone [88].

**Phylogenetic relation:** *C. croucheri* is known from a single, poorly preserved skull, thus the validity of *C. croucheri* is dubious [14]. Thus, it is not further considered here.

1. ***Blattoidealestes gracilis***

**Where:** Prince Albert Road in the Prince Albert Division in the Koup, Prince Albert Distict, Western Cape Province, South Africa [91].

**When:** *Tapinocephalus* zone [88].

**Phylogenetic relation:** *B. gracilis* is known from a single, poorly preserved skull and dentary, thus the validity of *B. gracilis* is dubious [14]. Thus, it is not further considered here.

**Amniote chrone 11 (Am11)**

1. ***Karenites ornamentatus***

**Where:** Kotelnich locality, Kotel’nichskii District, Kirov Region, Russian Federation [87].

**When:** Kotelnich locality, *Deltavjatia vjatkensis* zone [87], equivalent to the *Pristerognathus* zone (see Fig. 2).

**Phylogenetic relation:** *Karenites ornamentatus* was found to be closely related to *Mupashi migrator* within the eutherocephalian family Baurioidea [70].

1. ***Perplexisaurus foveatus***

**Where:** Kotelnich locality, Kotel’nichskii District, Kirov Region, Russian Federation [87].

**When:** Kotelnich locality, *Deltavjatia vjatkensis* zone [87], equivalent to the *Pristerognathus* zone (see Fig. 2).

**Phylogenetic relation:** Phylogenetic analysis identified *Perplexisaurus foveatus* as a non-lycosuchid, non-scylacosaurid therocephalian and sister taxon to Eutherocephalia [70].

1. ***Viatkosuchus sumini***

**Where:** Kotelnich locality, Kotel’nichskii District, Kirov Region, Russian Federation [87].

**When:** Kotelnich locality, *Deltavjatia* zone [87], equivalent to the *Pristerognathus* zone (see Fig. 2).

**Phylogenetic relation:** *Viatkosuchus sumini* is a therocephalian, within the Whaitsiaoidea [92] or Eutherocephalia [70].

1. ***Lycosuchus vanderrieti***

**Where:** Various localities of South Africa [14, and references therein].

**When:** The certain stratigraphic range of *Lycosuchus* extends from a horizon 2450 m above the base of the Abrahamskraal Formation at its thickest point in the uppermost Moordenaars Member to the top of the Poortjie Member of the Teekloof Formation [14, 16], upper *Tapinocephalus* to *Pristerognathus* zone (see Fig. 2).

**Phylogenetic relation:** *Lycosuchus* and *Simorhinella* are currently the only recognized genera in the family Lycosuchidae [89].

1. ***Glanosuchus macrops***

**Where:** Various localities of South Africa [14, and references therein; 88];

**When:** The stratigraphic range of *Glanosuchus* is between 700 m above the base of the Abrahamskraal Formation at its thickest point (upper Combrinkskraal Member) to the top of the Poortjie Member of the Teekloof Formation [14, 16], upper *Tapinocephalus* to *Pristerognathus* zone (see Fig. 2).

**Phylogenetic relation:** A therocephalian within the family Scylacosauridae [70].

1. ***Scylacosuchus orenburgensis***

**Where:** Vyazovka 5 locality, Orenburgskii District, Orenburg Region, Russian Federation [87].

**When:** *Proelginia* Assemblage Zone [87], equivalent to the *Tropidostoma* zone (see Fig. 2).

**Phylogenetic relation:** *Scylacosuchus orenburgensis* is a eutherocephalian [70].

1. ***Choerosaurus dejageri***

**Where:** Kuilspoort, Beaufort West, Western Cape Province, South Africa [93].

**When:** *Tropidostoma* zone [93].

**Phylogenetic relation:** *Choerosaurus dejageri* is an eutherocephalian, member of the Baurioidea [70, 92].

1. ***Ictidosuchus primaevus***

**Where:** NE of Pearston, Cape Colony, South Africa [94].

**When:** *Tropidostoma* zone [92].

**Phylogenetic relation:** *Ictidosuchus primaevus* is closely related to *Ictidosuchoides longiceps* and *Ictidosuchops rubidgei* within the eutherocephalian Baurioidea [70, 92].

1. ***Ictidosuchoides longiceps***

**Where:** Various localities of South Africa [95].

**When:** *Tropidostoma, Cistecephalus,* and upper *Daptocephalus* zones [92].

**Phylogenetic relation:** *Ictidosuchoides longiceps* is closely related to *Ictidosuchoides primaevus* and *Ictidosuchops rubidgei* within the eutherocephalian Baurioidea [70, 92].

1. ***Microwhaitsia mendrezi***

**Where:** Farm Badshoek, near Beaufort West district, Western Cape Province, South Africa [92].

**When:** Upper *Tropidostoma* zone [92].

**Phylogenetic relation:** *M. mendrezi* is a member of the eutherocephalian Whaitsiaoidea [92].

1. ***Hofmeyria atavus***

**Where:** Kookfontein, Victoria West, Northern Cape, and Graaff-Reinet Commonage, Eastern Cape, South Africa [96].

**When:** *Tropidostoma and Cistecephalus* zones [92].

**Phylogenetic relation:** *Hofmeyria* is a member of the family Hofmeyriidae [70] within *the* eutherocephalian Whaitsiaoidea [92].

1. ***Ictidostoma hemburyi***

**Where:** Northwest of Richmond and Beaufort West District, South Africa [97].

**When:** *Tropidostoma and Cistecephalus* zones [92].

**Phylogenetic relation:** *Ictidostoma hemburyi* is a member of the family Hofmeyriidae [70] within the eutherocephalian Whaitsiaoidea [92].

1. ***Charassognathus gracilis***

**Where:** Between the towns Leeu Gamka and Fraserburg in the Beaufort West district, Western Cape Province, South Africa [98].

**When:** Hoedemaker Member of the Teekloof Formation, *Tropidostoma* zone [98].

**Phylogenetic relation:** *Charassognathus gracilis* is the oldest member of Cynodontia [98] and forms a clade with *Dvinia prima* and *Procynosuchus delaharpeae* [70].

1. ***Gorynychus masyutinae***

**Where:** Kotelnich locality, Kotel’nichskii District, Kirov Region, Russian Federation [99].

**When:** Kotelnich locality, Vanyushonki Member (lowest), Kotelnich subassemblage [99], equivalent to the *Pristerognathus* zone (see Fig. 2).

**Phylogenetic relation:** Phylogenetic analysis identified *Gorynychus* as a non-lycosuchid, non-scylacosaurid therocephalian and sister taxon to Eutherocephalia [99].

**Amniote chrone 12 (Am12)**

1. ***Ictidosuchoides longiceps***

**Where:** Various localities of South Africa [95].

**When:** *Tropidostoma, Cistecephalus,* and upper *Daptocephalus* zones [92].

**Phylogenetic relation:** *Ictidosuchoides longiceps* is closely related to *Ictidosuchoides primaevus* and *Ictidosuchops rubidgei* within the eutherocephalian Baurioidea [70, 92].

1. ***Hofmeyria atavus***

**Where:** Kookfontein, Victoria West, Northern Cape, and Graaff-Reinet Commonage, Eastern Cape, South Africa [96].

**When:** *Tropidostoma and Cistecephalus* zones [92].

**Phylogenetic relation:** *Hofmeyria* is a member of the family Hofmeyriidae [70] within the eutherocephalian Whaitsiaoidea [92].

1. ***Mupashi migrator***

**Where:** Locality L58, Luangwa Basin, Zambia [100].

**When:** Upper Madumabisa Mudstone Formation, equivalent to the *Cistecephalus* zone [100].

**Phylogenetic relation:** *Mupashi migrator* was found to be closely related to the Russian *Karenites ornamentatus* within the eutherocephalian Baurioidea [70, 100].

1. ***Lycideops longiceps***

**Where:** Thaba Nchu Commonage, Free State, South Africa [101].

**When:** *Daptocephalus* zone [92].

**Phylogenetic relation:** *L. longiceps* is an eutherocephalian, member of the Baurioidea [70, 100].

1. ***Tetracynodon tenuis***

**Where:** Graaff-Reinet District, Eastern Cape Province, South Africa [102].

**When:** *Daptocephalus* zone [92].

**Phylogenetic relation:** *Tetracynodon tenuis* is an eutherocephalian, member of the Baurioidea [70, 100].

1. ***Ictidosuchops rubidgei***

**Where:** Dornkloof, Eastern Cape, South Africa [103].

**When:** Lower *Daptocephalus* zone [92].

**Phylogenetic relation:** *Ictidosuchops rubidgei* is closely related to the eutherocephalians *Ictidosuchoides primaevus* and *Ictidosuchoides longiceps* within the Baurioidea [70, 92].

1. ***Theriognathus microps***

**Where:** South Africa, Tanzania, and Zambia [104].

**When:** Lower *Daptocephalus* zone [92].

**Phylogenetic relation:** *Theriognathus microps* is a member of the eutherocephalian Whaitsiaoidea [92].

1. ***Ictidochampsa platyceps***

**Where:** New Bethesda Commonage, Eastern Cape, South Africa [105].

**When:** Lower *Daptocephalus* zone [92].

**Phylogenetic relation:** *Ictidochampsa platyceps* is a member of the eutherocephalian Whaitsiaoidea [92].

1. ***Ophidostoma tatarinovi***

**Where:** Farm Good Luck, near Fraserburg District, Northern Cape Province, South Africa [92].

**When:** Upper *Cistecephalus* zone [92].

**Phylogenetic relation:** *Ophidostoma tatarinovi* is a member of the eutherocephalian Whaitsiaoidea [92].

1. ***Mirotenthes digitipes***

**Where:** South Africa [106]:

1. UCMP Locality V3695, Murraysburg, Western Cape.
2. Farm Good Luck, near Fraserburg District, Northern Cape Province

**When:** Lower *Daptocephalus* zone [92].

**Phylogenetic relation:** *Mirotenthes digitipes* is a member of the family Hofmeyriidae [70] within the eutherocephalian Whaitsiaoidea [92].

1. ***Annatherapsidus petri***

**Where:** Sokolki, Savvatii, and Blumental 3 localities, Kotlasskii District, Arkhangelsk Region, Russian Federation [87].

**When:** Collection PIN 2005, *Scutosaurus* Assemblage Zone [87], equivalent to the lower *Daptocephalus* (see Fig. 2).

**Phylogenetic relation:** *Annatherapsidus petri* is a member of the eutherocephalian family Akidnognathidae [70]*.*

1. ***Shiguaignathus wangi***

**Where:** Locality DQS 28, Nei Mongol, China [107].

**When:** Member III of the Naobaogou Formation, Late Permian [107].

**Phylogenetic relation:** *Shiguaignathus wangi* is a member of the eutherocephalian family Akidnognathidae [70] and is closely related to *Annatherapsidus petri.* However, its age is not well-constrained. Thus, it is not further considered here.

1. ***Promoschorhynchus platyrhinus***

**Where:** Various localities of South Africa [108].

**When:** *Daptocephalus* zone [92].

**Phylogenetic relation:** *Promoschorhynchus platyrhinus* is a member of the eutherocephalian family Akidnognathidae [70]*.*

1. ***Cerdosuchoides brevidens***

**Where:** East of Wapadsberg, Cradock District, Eastern Cape Province, South Africa [109].

**When:** *Daptocephalus* zone [92].

**Phylogenetic relation:** *Cerdosuchoides* *brevidens* is a member of the eutherocephalian family Akidnognathidae and is closely related to *Moschorhinus kitchingi* [70]*.*

1. ***Moschorhinus kitchingi***

**Where:** Various localities of South Africa [110].

**When:** *Daptocephalus* zone [92].

**Phylogenetic relation:** *Moschorhinus kitchingi* is a member of the eutherocephalian family Akidnognathidae and is closely related to *Cerdosuchoides* *brevidens* [70]*.*

1. ***Euchambersia mirabilis***

**Where:** Farm Vanwyksfontein, Northern Cape, South Africa [111].

**When:** *Cistecephalus* zone [92].

**Phylogenetic relation:** *Euchambersia mirabilis* is a member of the eutherocephalian family Akidnognathidae and is closely related to *Moschorhinus kitchingi* and *Cerdosuchoides* *brevidens* [70]*.*

1. ***Chthonosaurus velocidens***

**Where:** Pronkino locality, Sorochinskii District, Orenburg Region, Russian Federation [87].

**When:** Collection PIN 521, *Scutosaurus* Assemblage Zone [87], equivalent to the lower *Daptocephalus* (see Fig. 2).

**Phylogenetic relation:** *Chthonosaurus velocidens* is an eutherocephalian closely related to *Ichibengops munyamadziensis* [70, 92].

1. ***Ichibengops munyamadziensis***

**Where:** Locality L56, Luangwa Basin, Zambia [112].

**When:** Upper Madumabisa Mudstone Formation, equivalent to the *Cistecephalus* zone [100].

**Phylogenetic relation:** *Ichibengops munyamadziensis* is an eutherocephalian closely related to *Chthonosaurus velocidens* [70, 92].

1. ***Dvinia prima***

**Where:** Russian Federation [87].

**When:** Sokolki locality, Collection PIN 2005, *Scutosaurus* Assemblage Zone [87], equivalent to the lower *Daptocephalus* (see Fig. 2).

**Phylogenetic relation:** *D. prima* is a basal cynodont that forms a clade with *Charassognathus gracilis* and *Procynosuchus delaharpeae* [70].

1. ***Procynosuchus delaharpeae***

**Where:** South Africa, Tanzania and Germany [113].

**When:** *Procynosuchus* is from the *Tropidostoma* to *Daptocephalus* zones [12].

**Phylogenetic relation:** *Procynosuchus delaharpeae* is a basal cynodont that forms a clade with *Charassognathus gracilis* and *D. prima* [70].

1. ***Akidnognathus parvus***

**Where:** Eastern Cape, South Africa [114].

**When:** *Cistecephalus zone* [114].

**Phylogenetic relation:** *Akidnognathus parvus* is a basal member of the eutherocephalian family Akidnognathidae [99].

**References**

1. Kammerer, C. F. Systematics of the Anteosauria (Therapsida: Dinocephalia). *J Syst Palaeontol* **9**, 261–304 (2011).
2. Paleobiology Database. Search for *Syodon biarmicum.* Access on May 16, 2020. <https://paleobiodb.org/classic/basicTaxonInfo?taxon_no=357010>
3. Naugolnykh, S. V. Fossil flora and stratigraphy of the terrigenous Kungurian beds (Lower Permian) of the basin of the Barda River (Urals, Perm Krai). *Stratigr Geol Correl* **22**, 680–707 (2014).
4. Lucas, S. G. Permian tetrapod biochronology, correlation and evolutionary events. *Geol Soc Spec Publ* **450**, 405–444 (2017).
5. Cisneros, J. C. *et al.* Carnivorous dinocephalian from the Middle Permian of Brazil and tetrapod dispersal in Pangaea. *PNAS* **109**, 1584–1588 (2012).
6. Ivakhnenko, M. F. Cranial morphology and evolution of Permian Dinomorpha (Eotherapsida) of Eastern Europe. *Paleontolog J* **42**, 859–995 (2008).
7. Liu, J. Osteology, ontogeny, and phylogenetic position of *Sinophoneus yumenensis* (Therapsida, Dinocephalia) from the Middle Permian Dashankou Fauna of China. *J Vertebr Paleontol* **33**, 1394–1407 (2013).
8. Rubidge, B. S. *Australosyodon*, the first primitive anteosaurid dinocephalian from the Upper Permian of Gondwana. *Palaeontology* **37**, 579–594 (1994).
9. Barbolini, N., Rubidge, B. & Bamford, M. K. A new approach to biostratigraphy in the Karoo retroarc foreland system: Utilising restricted-range palynomorphs and their first appearance datums for correlation. *J Afr Earth Sci* **140**, 114–133 (2018).
10. Ivakhnenko, M. F. Primitive Late Permian dinocephalian titanosuchids of Eastern Europe. *Paleontolog J* **29**, 120–129 (1995).
11. Rubidge, B. S. A new primitive dinocephalian mammal-like reptile from the Permian of southern Africa. *Palaeontology* **34**, 547–559 (1991).
12. Rubidge, B. S. Advances in Nonmarine Karoo Biostratigraphy: Significance for Understanding Basin Development in *Origin and Evolution of the Cape Mountains and Karoo Basin* (eds. Linol, B. & de Wit M. J.) 141–149 (Springer, 2016).
13. Paleobiology Database. Search for *Anteosaurus magnificus*. Access on May 16, 2020. <https://paleobiodb.org/classic/basicTaxonInfo?taxon_no=362285>
14. Day, M. O. Middle Permian continental biodiversity changes as reflected in the Beaufort Group of South Africa: a bio-and lithostratigraphic review of the *Eodicynodon*, *Tapinocephalus*, and *Pristerognathus* assemblage zones. PhD Thesis, Witwatersrand University; <http://hdl.handle.net10539/14014> (2013)
15. Carroll, R. L. *Vertebrate Paleontology and Evolution* (Freeman and Company, New York, 1988).
16. Day, M. O. *et al.* When and how did the terrestrial mid-Permian mass extinction occur? Evidence from the tetrapod record of the Karoo Basin, South Africa. *Proc R Soc Lond B Biol Sci* **282**, 20150834 (2015).
17. Güven, S., Rubidge, B. S. & Abdala, F. Cranial morphology and taxonomy of South African Tapinocephalidae (Therapsida: Dinocephalia): the case of *Avenantia* and *Riebeeckosaurus*. *Palaeontologia Africana* **48**, 24–33 (2013).
18. Güven, S., Rubidge, B. S. & Abdala, F. Taxonomy of tapinocephalid Dinocephalia from the South African Karoo Basin. Research abstract in the 16th Biennial Conference of the Palaeontological Association of Southern Africa, Cape Town. *Palaeontologia Africana* **47**, 29 (2012).
19. Rubidge, B. S. & Hopson, J. A. A primitive anomodont therapsid from the base of the Beaufort Group (Upper Permian) of South Africa. *Zool J Linn Soc* **117**, 115–139 (1996).
20. Castanhinha, R. Bringing dicynodonts back to life: paleobiology and anatomy of a new emydopoid genus from the Upper Permian of Mozambique. *PLoS ONE* **8**, e80974, <https://doi.org/10.1371/journal.pone.0080974> (2013).
21. Kammerer, C. F., Angielczyk, K. D. & Fröbisch, D. A comprehensive taxonomic revision of *Dicynodon* (Therapsida, Anomodontia) and its implications for Dicynodont phylogeny, biogeography, and biostratigraphy. *J Vertebr Paleontol* **31** (Suppl. to No 6), 1–158 (2011).
22. Fröbisch, J. & Reisz, R. R. The postcranial anatomy of *Suminia getmanovi* (Synapsida: Anomodontia), the earliest known arboreal tetrapod. *Zool J Linn Soc* **162**, 661–698 (2011).
23. Rubidge, B. S. The cranial morphology of a new species of the genus *Eodicynodon* (Therapsida, Dicynodontia). *Navorsinge van die Nasionale Museum Bloemfontein* **7**, 29–42 (1990).
24. Angielczyk, K. D., Rubidge, B. S., Day, M. O. & Lin, F. A reevaluation of *Brachyprosopus broomi* and *Chelydontops altidentalis*, dicynodonts (Therapsida, Anomodontia) from the middle Permian *Tapinocephalus* Assemblage Zone of the Karoo Basin, South Africa. *J Vertebr Paleontol* **36**, e1078342 (2016).
25. Paleobiology Database. Search for *Biseridens qilianicus*. Access on May 16, 2020. <https://paleobiodb.org/classic/basicTaxonInfo?taxon_no=137794>
26. Modesto, S., Rubidge, B. & Welman, J. The most basal anomodont therapsid and the primacy of Gondwana in the evolution of anomodonts. *Proc R Soc Lond B Biol Sci* **266**, 331–337 (1999).
27. Modesto, S., Rubidge, B., Visser, I. & Welman, J. A new basal dicynodont from the Upper Permian of South Africa. *Palaeontology* **46**, 211–223 (2003).
28. Kammerer, C.F. & Smith, R. M. H. An early geikiid dicynodont from the *Tropidostoma* Assemblage Zone (late Permian) of South Africa. *PeerJ* **5**, e2913 (2017).
29. Sidor, C. A. *et al.* Tetrapod fauna of the lowermost Usili Formation (Songea Group, Ruhuhu Basin) of Southern Tanzania, with a new burnetiid record. *J Vertebr Paleontol* **30**, 696–703 (2010).
30. Boos, A. D. S., Kammerer, C. F., Schultz, C. L., Soares, M. B. & Ilha, A. L. R. A new dicynodont (Therapsida: Anomodontia) from the Permian of Southern Brazil and its implications for bidentalian origins. *PLoS ONE* **11**, e0155000 (2016).
31. Cisneros, J. C., Abdala, F., Rubidge, B. S., Dentzien-Dias, P. C., & Bueno, A. O. Dental occlusion in a 260-million-year-old therapsid with saber canines from the Permian of Brazil. *Science* **331**, 1603–1605 (2011).
32. Paleobiology Database Search for *Oudenodon*. Access on May 16, 2020. <https://paleobiodb.org/classic/basicTaxonInfo?taxon_no=39052>
33. Paleobiology Database. Search for *Tropidostoma dubium*. Access on May 16, 2020. <https://paleobiodb.org/classic/basicTaxonInfo?taxon_no=331367>
34. Paleobiology Database. Search for *Rhachiocephalus magnus*. Access on May 16, 2020. <https://paleobiodb.org/classic/basicTaxonInfo?taxon_no=338940>
35. Kammerer, C. F., Bandyopadhyay, S. & Ray, S. A new taxon of cistecephalid dicynodont from the upper Permian Kundaram Formation of India. *Pap Palaeontol* **2**, 569–584 (2016).
36. Fröbisch, J. & Reisz, R. R. A new species of *Emydops* (Synapsida, Anomodontia) and a discussion of dental variability and pathology in dicynodonts. *J Vertebr Paleontol* **28**, 770–787 (2008).
37. Kammerer, C. F., Angielczyk, K. D., & Fröbisch, J. Redescription of *Digalodon rubidgei*, an emydopoid dicynodont (Therapsida, Anomodontia) from the Late Permian of South Africa. *Mitt Mus Nat Berl Foss Rec* **18**, 43–55 (2015).
38. Viglietti, P. A. *et al.* The *Daptocephalus* Assemblage Zone (Lopingian), South Africa: a proposed biostratigraphy based on a new compilation of stratigraphic ranges. *J Afr Earth Sci* **113**, 153e164 (2016).
39. Paleobiology Database. Search for *Cistecephalus microrhinus*. Access on May 16, 2020. <https://paleobiodb.org/classic/basicTaxonInfo?taxon_no=56851>
40. Paleobiology Database. Search for *Cistecephaloides boonstrai*. Access on May 16, 2020. <https://paleobiodb.org/classic/basicTaxonInfo?taxon_no=345907>
41. Fröbisch, J. The cranial anatomy of *Kombuisia frerensis* Hotton (Synapsida, Dicynodontia) and a new phylogeny of anomodont therapsids. *Zool J Linn Soc* **150**, 117–144 (2007).
42. Angielczyk, K. D. Permian and Triassic dicynodont (Therapsida: Anomodontia) faunas of the Luangwa Basin, Zambia: taxonomic update and implications for dicynodont biogeography and biostratigraphy in *The Early Evolutionary History of Synapsida* (eds. Kammerer, C. F., Angielczyk, K. D. & Fröbisch, J.) 93–138 (Springer, Dordrecht, 2014).
43. Paleobiology Database 3. Search for *Kawingasaurus fossilis*. Access on May 16, 2020. <https://paleobiodb.org/classic/basicTaxonInfo?taxon_no=346140>
44. Angielczyk, K. D. *et al.* New dicynodonts (Therapsida, Anomodontia) an updated tetrapod stratigraphy of the Permian Ruhuhu Formation (Songea Group, Ruhuhu Basin) of southern Tanzania. *J Vertebr Paleontol* **34**, 1408–1426 (2014).
45. Paleobiology Database. Search for *Odontocyclops whaitsi*. Access on May 16, 2020. <https://paleobiodb.org/classic/basicTaxonInfo?taxon_no=339818>
46. Paleobiology Database. Search for *Aulacephalodon bainii*. Access on May 16, 2020. <https://paleobiodb.org/classic/basicTaxonInfo?taxon_no=333234>
47. Kammerer, C. F., Angielczyk, K. D. & Fröbisch, J. Redescription of the geikiid *Pelanomodon* (Therapsida, Dicynodontia), with a reconsideration of ‘*Propelanomodon*’. *J Vertebr Paleontol* **36**, e1030408 (2016).
48. Maisch, M. W. & Gebauer, E. V. I. Reappraisal of *Geikia locusticeps* (Therapsida: Dicynodontia) from the Upper Permian of Tanzania. *Palaeontology* **48**, 309–324 (2005).
49. Paleobiology Database. Search for *Rhachiocephalus magnus*. Access on May 16, 2020. <https://paleobiodb.org/classic/basicTaxonInfo?taxon_no=338940>
50. Paleobiology Database. Search for *Rhachiocephalus behemoth*. Access on May 16, 2020. <https://paleobiodb.org/classic/basicTaxonInfo?taxon_no=345574>
51. Paleobiology Database. Search for *Kitchinganomodon crassus*. Access on May 16, 2020. <https://paleobiodb.org/classic/basicTaxonInfo?taxon_no=340227>
52. Sennikov, A. G. & Golubev, V. K. Sequence of Permian tetrapod faunas of Eastern Europe and the Permian–Triassic ecological crisis. *Paleontolog J* **51**, 600–611 (2017).
53. Maisch, M. W. A new basal lystrosaurid dicynodont from the Upper Permian of South Africa. *Palaeontology* **45**, 343–359 (2002).
54. Kurkin, A. A. New Late Permian dicynodonts from the Vyazniki Assemblage of terrestrial tetrapods of Eastern Europe. *Paleontolog J* **35**, 53–59 (2001).
55. Day, M. O., Rubidge, B. S. & Abdala, F. A new mid-Permian burnetiamorph therapsid from the Main Karoo Basin of South Africa and a phylogenetic review of Burnetiamorpha. *Acta Palaeontol Pol* **61**, 701–719; <https://doi.org/10.4202/app.00296.2016> (2016).
56. Kammerer, C. F. Two unrecognized burnetiamorph specimens from historic Karoo collections. *Palaeontologia Africana* **50**, 64–75 (2016).
57. Rubidge, B. S., Sidor, C. A. & Modesto, S. P. A new burnetiamorph (Therapsida: Biarmosuchia) from the Middle Permian of South Africa. *J Paleontol* **80**, 740–749 (2006).
58. Rubidge, B. S. & Kitching, J. W. A new burnetiamorph (Therapsida: Biarmosuchia) from the lower Beaufort Group of South Africa. *Palaeontology* **46**, 199–210 (2003).
59. Sidor, C. A., Hopson, J. A. & Keyser, A. W. A new burnetiamorph therapsid from the Teekloof Formation, Permian, of South Africa. *J Vertebr Paleontol* **24**, 938–950 (2004).
60. Sidor, C. A. The first biarmosuchian from the upper Madumabisa Mudstone Formation (Luangwa Basin) of Zambia. *Palaeontologia Africana* **49**, 1–7 (2015).
61. Sidor, C. A. & Smith, R. M. H. A second burnetiamorph therapsid from the Permian Teekloof Formation of South Africa and its associated fauna. *J Vertebr Paleontol* **27**, 420–430 (2007).
62. Sidor, C. A. The naris and palate of *Lycaenodon longiceps* (Therapsida: Biarmosuchia), with comments on their early evolution in the Therapsida. *J Paleontol* **77**, 977–984 (2003).
63. Smith, R. M., Rubidge, B. S. & Sidor, C. A. A new burnetiid (Therapsida: Biarmosuchia) from the Upper Permian of South Africa and its biogeographic implications. *J Vertebr Paleontol* **26**, 331–343 (2006).
64. Rubidge, B. S. & Sidor, C. A. On the cranial morphology of the basal therapsids *Burnetia* and *Proburnetia* (Therapsida: Burnetiidae). *J Vertebr Paleontol* **22**, 257–267 (2002).
65. Kruger, A., Rubidge, B. S., Abdala, F., Chindebvu, E. G., & Jacobs, L. L. *Lende chiweta*, a new therapsid from Malawi, and its influence on burnetiamorph phylogeny and biogeography. *J Vertebr Paleontol* **35**, e1008698 (2015).
66. Sidor, C. A. & Welman, J. A second specimen of *Lemurosaurus pricei* (Therapsida: Burnetiamorpha). *J Vertebr Paleontol* **23**, 631–642 (2003).
67. Paleobiology Database. Search for *Ictidorhinus martinsi*. Access on May 16, 2020. <https://paleobiodb.org/classic/basicTaxonInfo?taxon_no=335891>
68. Sidor, C. A. & Rubidge, B. S. *Herpetoskylax hopsoni*, a new biarmosuchian (Therapsida: Biarmosuchia) from the Beaufort Group of South Africa in *Amniote Paleobiology: Perspectives on the Evolution of Mammals, Birds, and Reptiles* (eds. Carrano, M. T., Gaudin, T. J., Blob, R. W. & Wible, J. R.) 76–113 (Chicago Press, Chicago and London, 2006).
69. Kammerer, C. F., Smith, R. M. H., Day, M. O. & Rubidge, B. S. New information on the morphology and stratigraphic range of the Mid-Permian gorgonopsian *Eriphostoma microdon* Broom, 1911. *Pap Palaeontol* **1**, 201–221 (2014).
70. Kammerer, C. F. & Masyutin, V. Gorgonopsian therapsids (*Nochnitsa gen. nov.* and *Viatkogorgon*) from the Permian Kotelnich locality of Russia. *PeerJ* **6**, e4954 (2018).
71. Paleobiology Database. Search for *Sauroctonus progressus*. Access on May 16, 2020. <https://paleobiodb.org/classic/basicTaxonInfo?taxon_no=341026>
72. Gebauer, E. V. I. Re-assessment of the taxonomic position of the specimen GPIT/RE/7113 (*Sauroctonus parringtoni* comb. nov., Gorgonopsia) in *Early Early Evolutionary History of the Synapsida* (eds. Kammerer, C. F., Angielczyk, K. D. & Fröbisch, J.) 185–207 (Springer Netherlands, 2014).
73. Paleobiology Database. Search for *Gorgonops torvus*. Access on May 17, 2020. <https://paleobiodb.org/classic/basicTaxonInfo?taxon_no=56786>
74. Bendel, E.-M., Kammerer, C. F., Kardjilov, N., Fernandez, V. & Fröbisch, J. Cranial anatomy of the gorgonopsian *Cynariops robustus* based on CT-reconstruction. *PLoS ONE* **13**, e0207367 (2018).
75. Kammerer, C. F. Systematics of the Rubidgeinae (Therapsida: Gorgonopsia). *PeerJ* **4**, e1608 (2016).
76. Paleobiology Database. Search for *Aelurognathus tigriceps*. Access on May 17, 2020. <https://paleobiodb.org/classic/basicTaxonInfo?taxon_no=335407>
77. Kammerer, C. F. Anatomy and relationships of the South African gorgonopsian *Arctops* (Therapsida, Theriodontia). *Pap Palaeontol* **3**, 583–611 (2017).
78. Paleofile Database. Search for *Arctops*. Access on May 18, 2020. <http://www.paleofile.com/Theriodontia/Arctops.asp>
79. Paleofile Database. Search for *Lycaenops*. Access on May 18, 2020. <http://www.paleofile.com/Theriodontia/Lycaenops.asp#Lycaenops>
80. Paleobiology Database. Search for *Smilesaurus ferox*. Access on May 17, 2020. <https://paleobiodb.org/classic/basicTaxonInfo?taxon_no=335437>
81. Paleobiology Database. Search for *Lycaenops ornatus*. Access on May 17, 2020. <https://paleobiodb.org/classic/basicTaxonInfo?taxon_no=341016>
82. Kammerer, C. F. Cranial osteology of *Arctognathus curvimola*, a short-snouted gorgonopsian from the Late Permian of South Africa. *Pap Palaeontol* **1**, 41–58 (2015).
83. Paleofile Database . Search for *Sycosaurus*. Access on May 18, 2020. <http://www.paleofile.com/Theriodontia/Sycosaurus.asp>
84. Paleobiology Database. Search for *Dinogorgon rubidgei*. Access on May 17, 2020. <https://paleobiodb.org/classic/basicTaxonInfo?taxon_no=335451>
85. Paleobiology Database. Search for *Rubidgea atrox*. Access on February 13, 2020. <https://paleobiodb.org/classic/basicTaxonInfo?taxon_no=335397>
86. Kammerer, C. F. Rediscovery of the holotype of *Clelandina major* Broom, 1948 (Gorgonopsia: Rubidgeinae) with implications for the identity of this species. *Palaeontologia Africana* **52**, 85–88 (2017).
87. Ivakhnenko, M. F. Permian and Triassic Therocephals (Eutherapsida) of Eastern Europe. *Paleontolog J* **45**, 981–1144 (2011).
88. Abdala, F., Rubidge, B. S. & Van Den Heever, J. The oldest Therocephalians (Therapsida, Eutheriodontia) and the early diversification of Therapsida. *Palaeontology* **51**, 1011–1024 (2008).
89. Abdala, F., Kammerer, C. F., Day, M. O., Jirah, S. & Rubidge, B. S. Adult morphology of the therocephalian *Simorhinella baini* from the middle Permian of South Africa and the taxonomy, paleobiogeography, and temporal distribution of the Lycosuchidae. *J Paleontol* **88**, 1139–1153 (2014).
90. Paleofile Database. Search for *Crapartinella croucheri*. Access on May 18, 2020. <http://www.paleofile.com/Theriodontia/Crapartinella.asp>
91. Paleobiology Database. Search for *Blattoidealestes gracilis*. Access on May 17, 2020. <https://paleobiodb.org/classic/basicTaxonInfo?taxon_no=378307>
92. Huttenlocker, A. K. & Smith, R. M. S. New whaitsioids (Therapsida: Therocephalia) from the Teekloof Formation of South Africa and therocephalian diversity during the end-Guadalupian extinction. *PeerJ* **5**, e3868 (2017).
93. Paleobiology Database. Search for *Choerosaurus dejageri*. Access on May 17, 2020. <https://paleobiodb.org/classic/basicTaxonInfo?taxon_no=270392>
94. Paleobiology Database. Search for *Ictidosuchus primaevus*. Access on May 17, 2020. <https://paleobiodb.org/classic/basicTaxonInfo?taxon_no=56835>
95. Paleobiology Database. Search for *Ictidosuchoides longiceps*. Access on May 17, 2020. <https://paleobiodb.org/classic/basicTaxonInfo?taxon_no=376352>
96. Paleobiology Database. Search for *Hofmeyria atavus*. Access on May 17, 2020. <https://paleobiodb.org/classic/basicTaxonInfo?taxon_no=367363>
97. Paleofile Database. Search for *Ictidostoma hemburyi*. Access on May 18, 2020. <http://www.paleofile.com/Theriodontia/Ictidostoma.asp>
98. Botha, J., Abdala, F. & Smith, R. The oldest cynodont: new clues on the origin and early diversification of the Cynodontia. *Zool J Linn Soc* **149**, 477–492 (2007).
99. Kammerer, C. F. & Masyutin, V. A new therocephalian (*Gorynychus masyutinae gen. et sp. nov*.) from the Permian Kotelnich locality, Kirov Region, Russia. *PeerJ* **6**, e4933 (2018).
100. Huttenlocker, A. K. & Sidor, C. A. The first karenitid (Therapsida, Therocephalia) from the upper Permian of Gondwana and the biogeography of Permo-Triassic therocephalians. *J Vertebr Paleontol* **36**, e1111897 (2016).
101. Paleobiology Database. Search for *Lycideops longiceps*. Access on May 17, 2020. <https://paleobiodb.org/classic/basicTaxonInfo?taxon_no=270393>
102. Paleobiology Database. Search for *Tetracynodon tenuis*. Access on May 16, 2020. <https://paleobiodb.org/classic/basicTaxonInfo?taxon_no=270394>
103. Paleobiology Database. Search for *Ictidosuchops rubidgei*. Access on May 16, 2020. <https://paleobiodb.org/classic/basicTaxonInfo?taxon_no=346931>
104. Paleobiology Database. Search for *Theriognathus microps*. Access on May 17, 2020. <https://paleobiodb.org/classic/basicTaxonInfo?taxon_no=343308>
105. Paleobiology Database. Search for *Ictidochampsa platyceps*. Access on May 17, 2020. <https://paleobiodb.org/classic/basicTaxonInfo?taxon_no=345741>
106. Paleobiology Database. Search for *Mirotenthes digitipes*. Access on May 17, 2020. <https://paleobiodb.org/classic/basicTaxonInfo?taxon_no=377034>
107. Liu, J. & Abdala, F. The tetrapod fauna of the upper Permian Naobaogou Formation of China: 1. *Shiguaignathus wangi gen. et sp. nov*., the first akidnognathid therocephalian from China. *PeerJ* **5**, e4150 (2017).
108. Paleobiology Database. Search for *Promoschorhynchus platyrhinus*. Access on May 17, 2020. <https://paleobiodb.org/classic/basicTaxonInfo?taxon_no=343201>
109. Paleofile Database. Search for *Cerdosuchoides brevidens*. Access on May 18, 2020. <http://www.paleofile.com/Theriodontia/Cerdosuchoides.asp>
110. Paleobiology Database. Search for *Moschorhinus kitchingi*. Access on May 17, 2020. <https://paleobiodb.org/classic/basicTaxonInfo?taxon_no=178202>
111. Paleobiology Database. Search for *Euchambersia mirabilis*. Access on May 17, 2020. <https://paleobiodb.org/classic/basicTaxonInfo?taxon_no=377640>
112. Huttenlocker, A. K., Sidor, C. A. & Angielczyk, K. D. A new eutherocephalian (Therapsida, Therocephalia) from the upper Permian Madumabisa Mudstone Formation (Luangwa Basin) of Zambia. *J Vertebr Paleontol* **35**, e969400 (2015).
113. Paleobiology Database. Search for *Procynosuchus delaharpeae*. Access on May 17, 2020. <https://paleobiodb.org/classic/basicTaxonInfo?taxon_no=323823>
114. Paleobiology Database. Search for *Akidnognathus parvus*. Access on May 17, 2020. <https://paleobiodb.org/classic/basicTaxonInfo?taxon_no=377639>


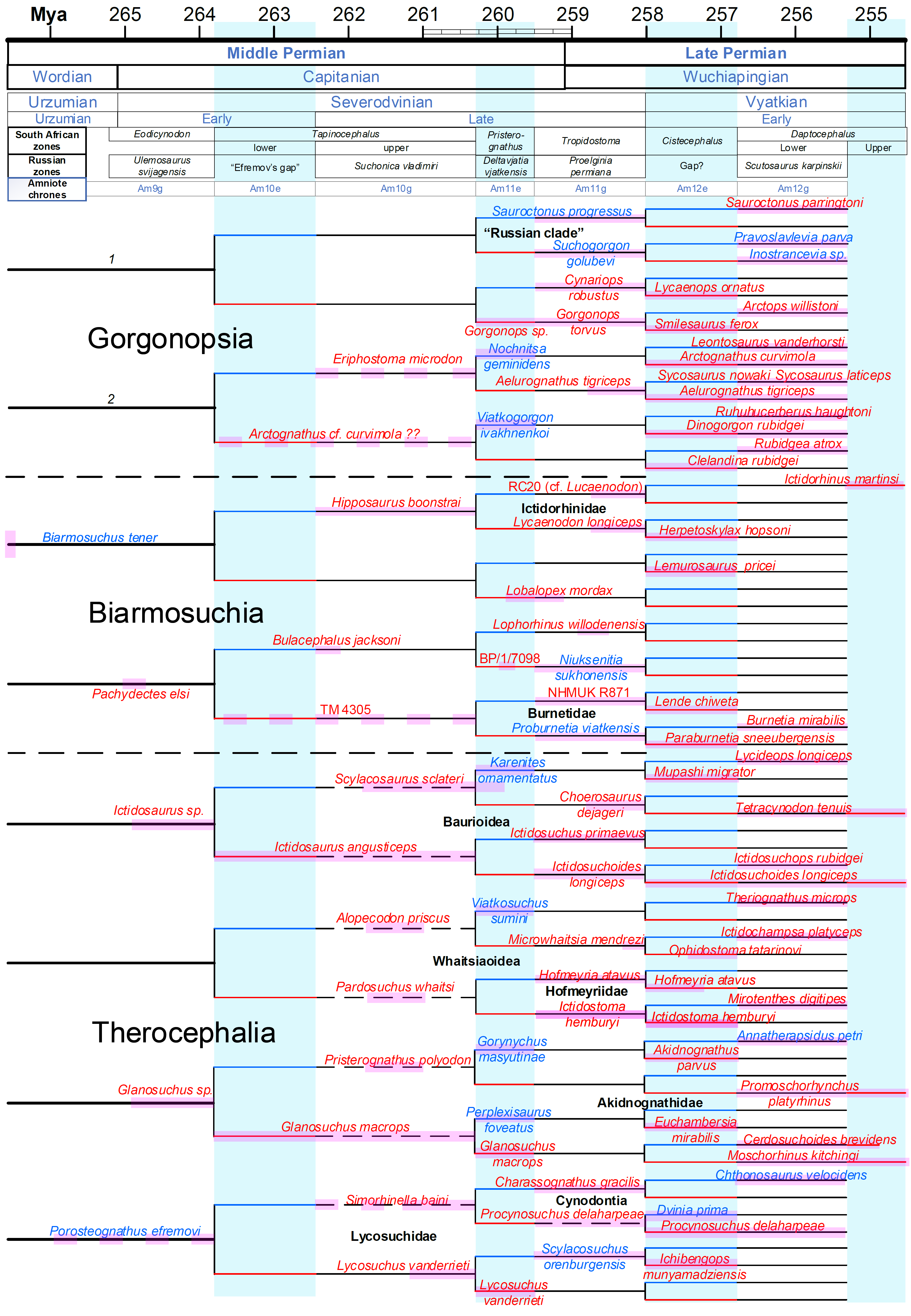


**Figure S1.**


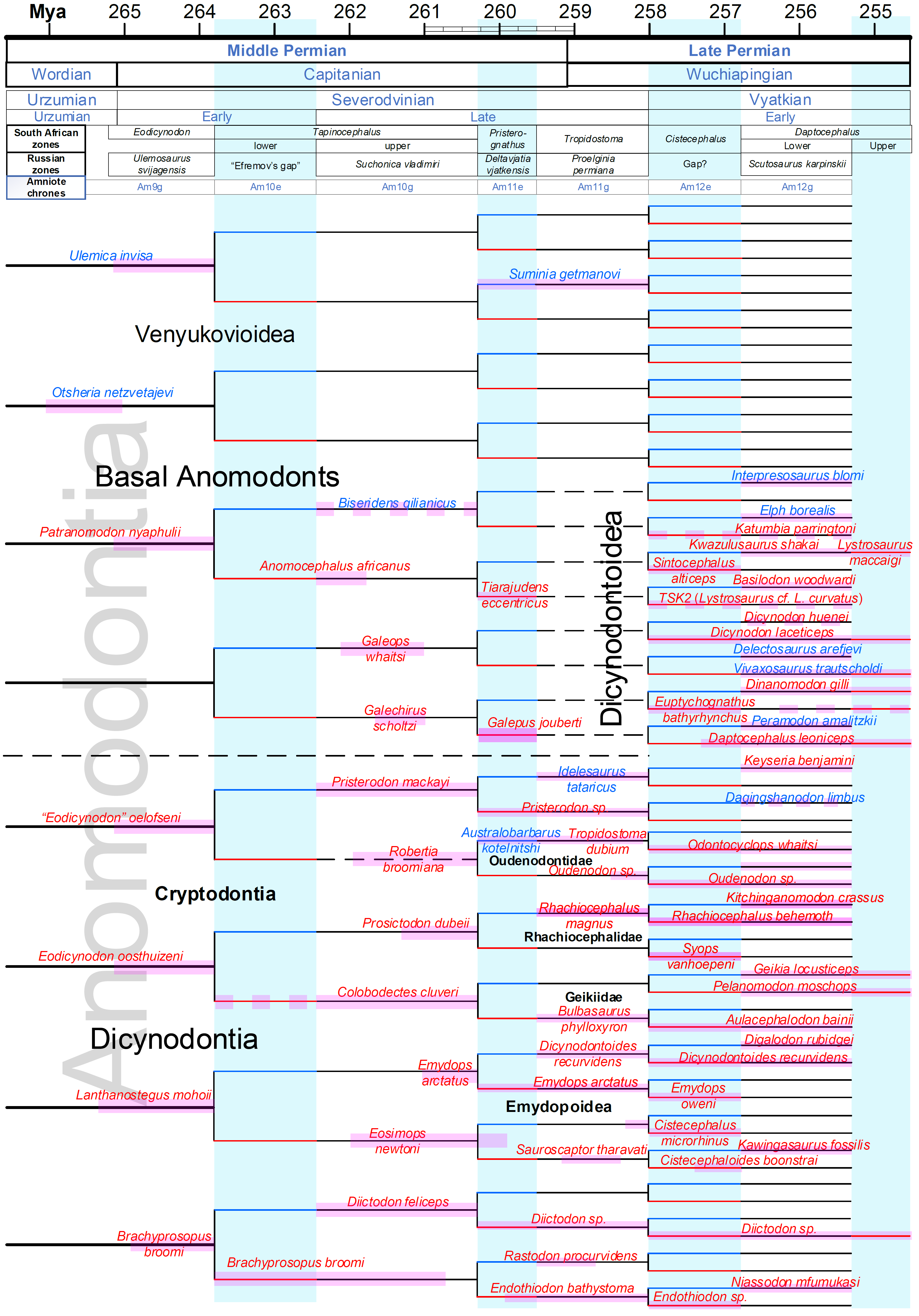


**Figure S2.**


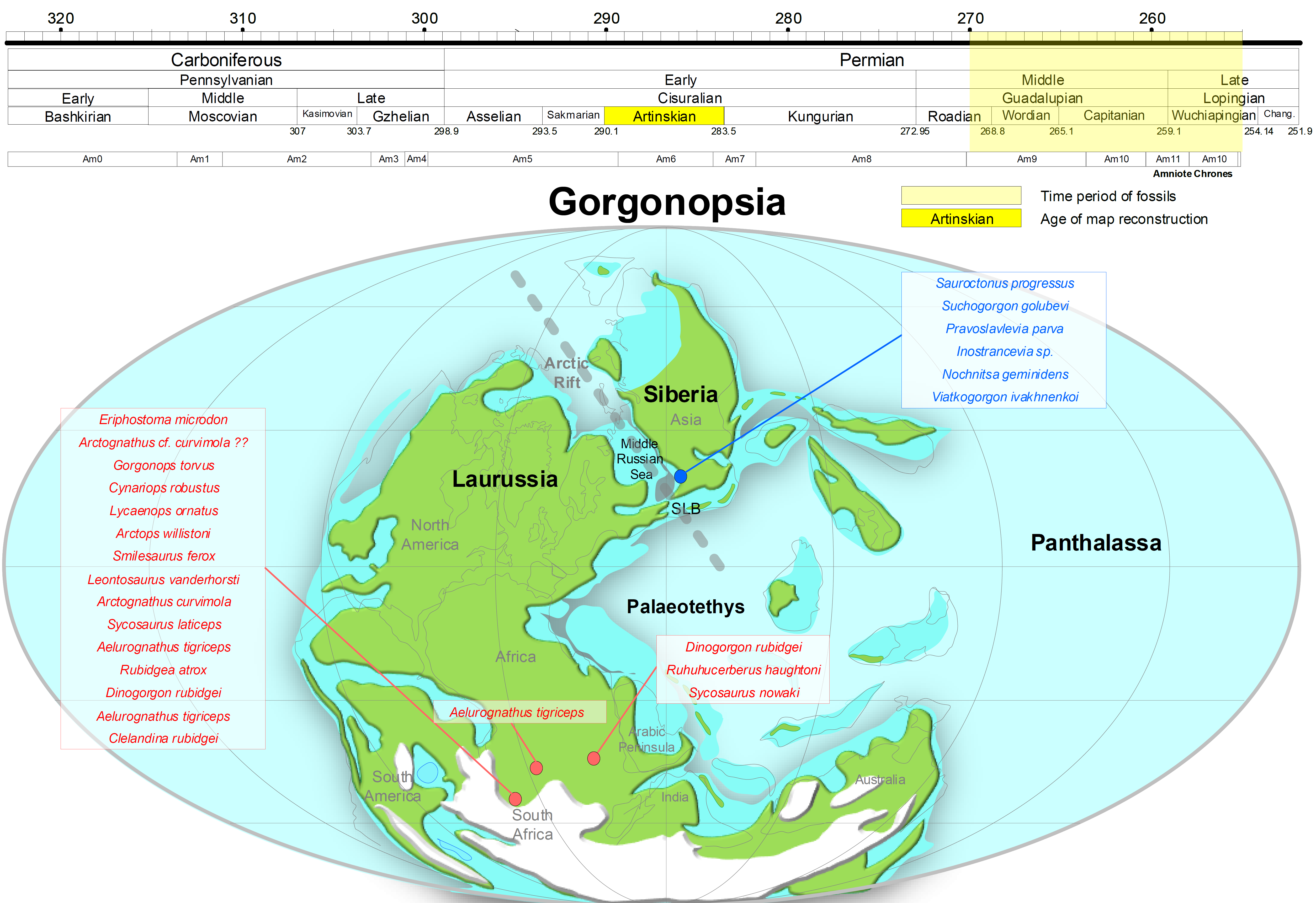


**Figure S3.**


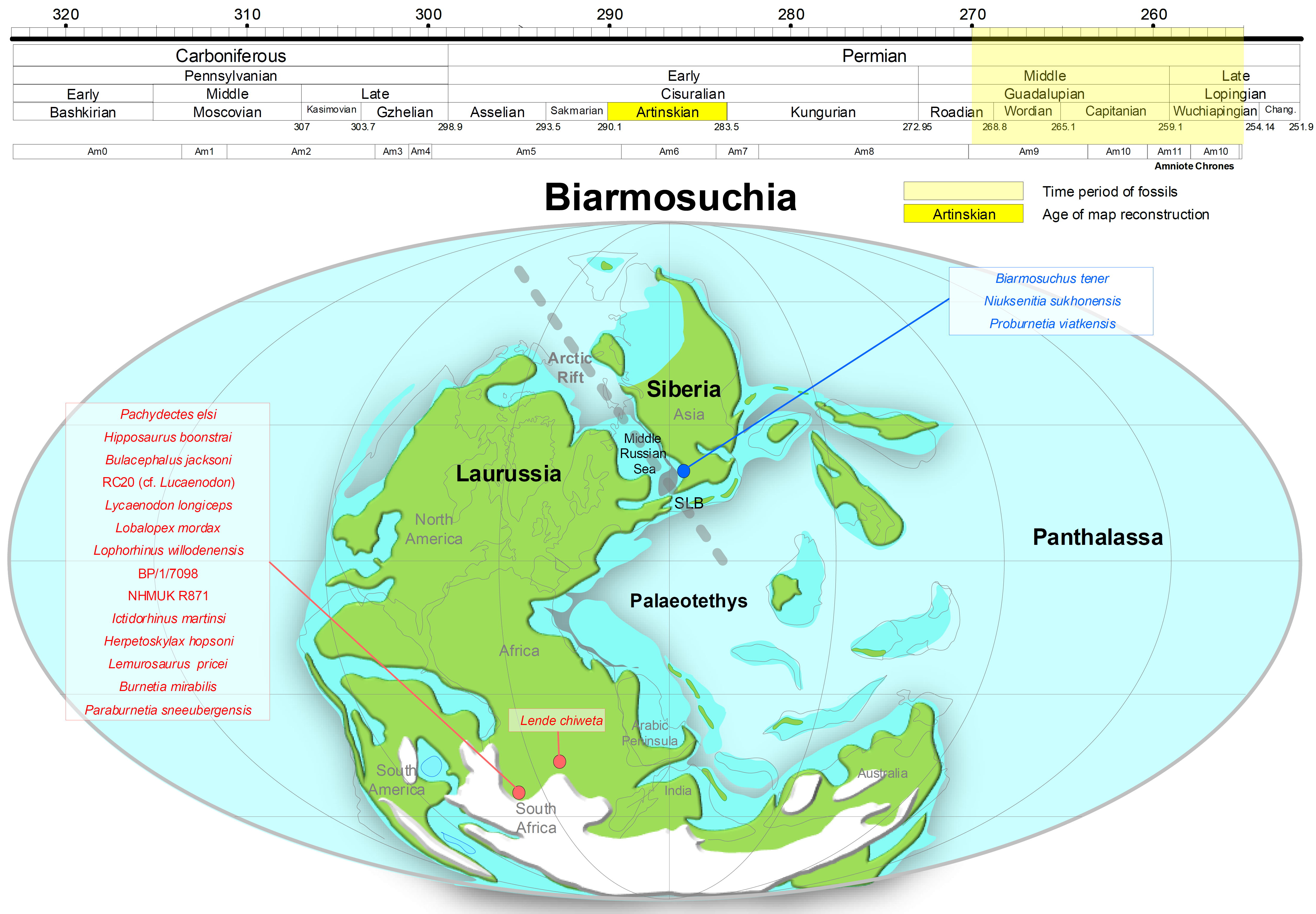


**Figure S4.**


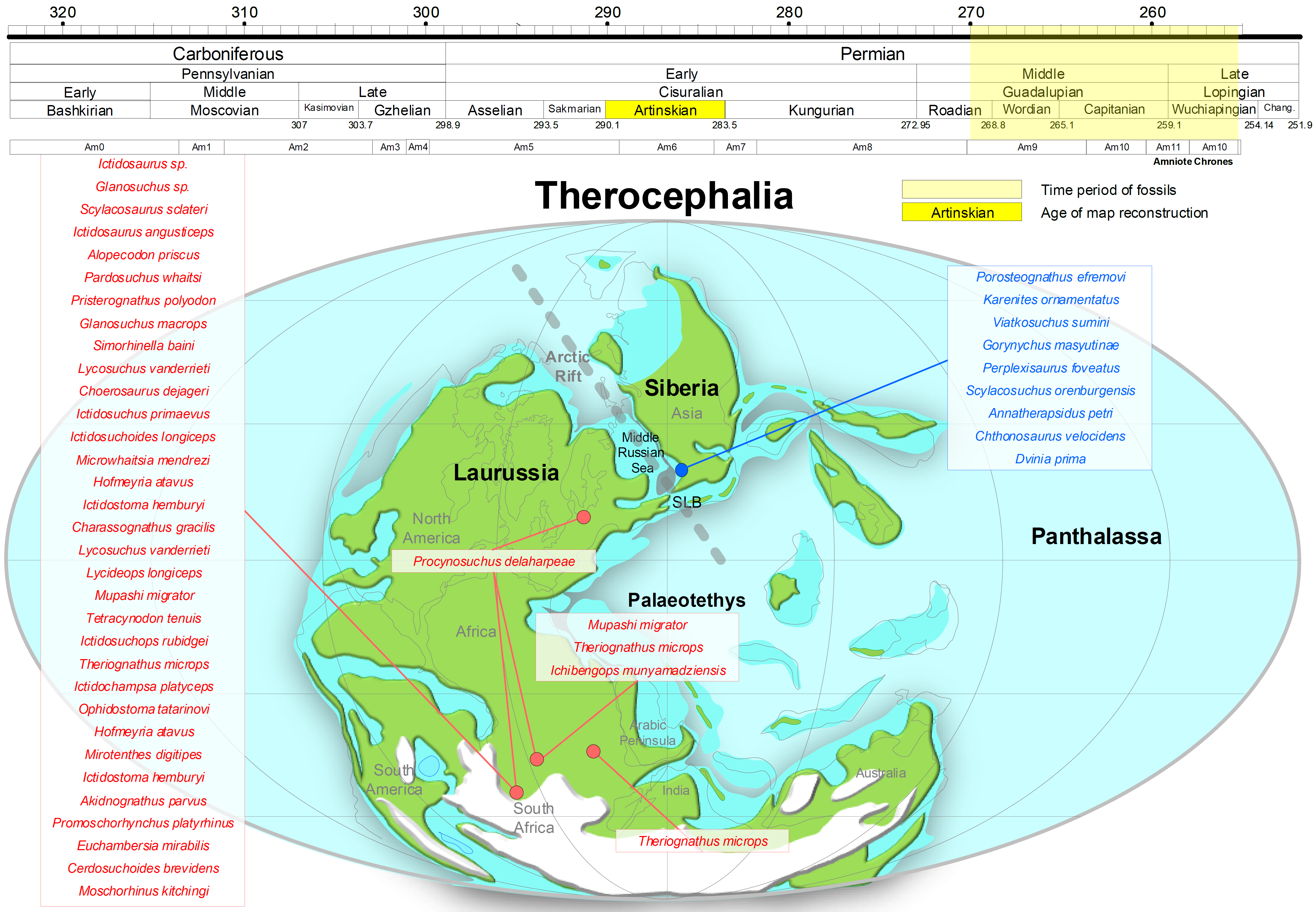


**Figure S5.**


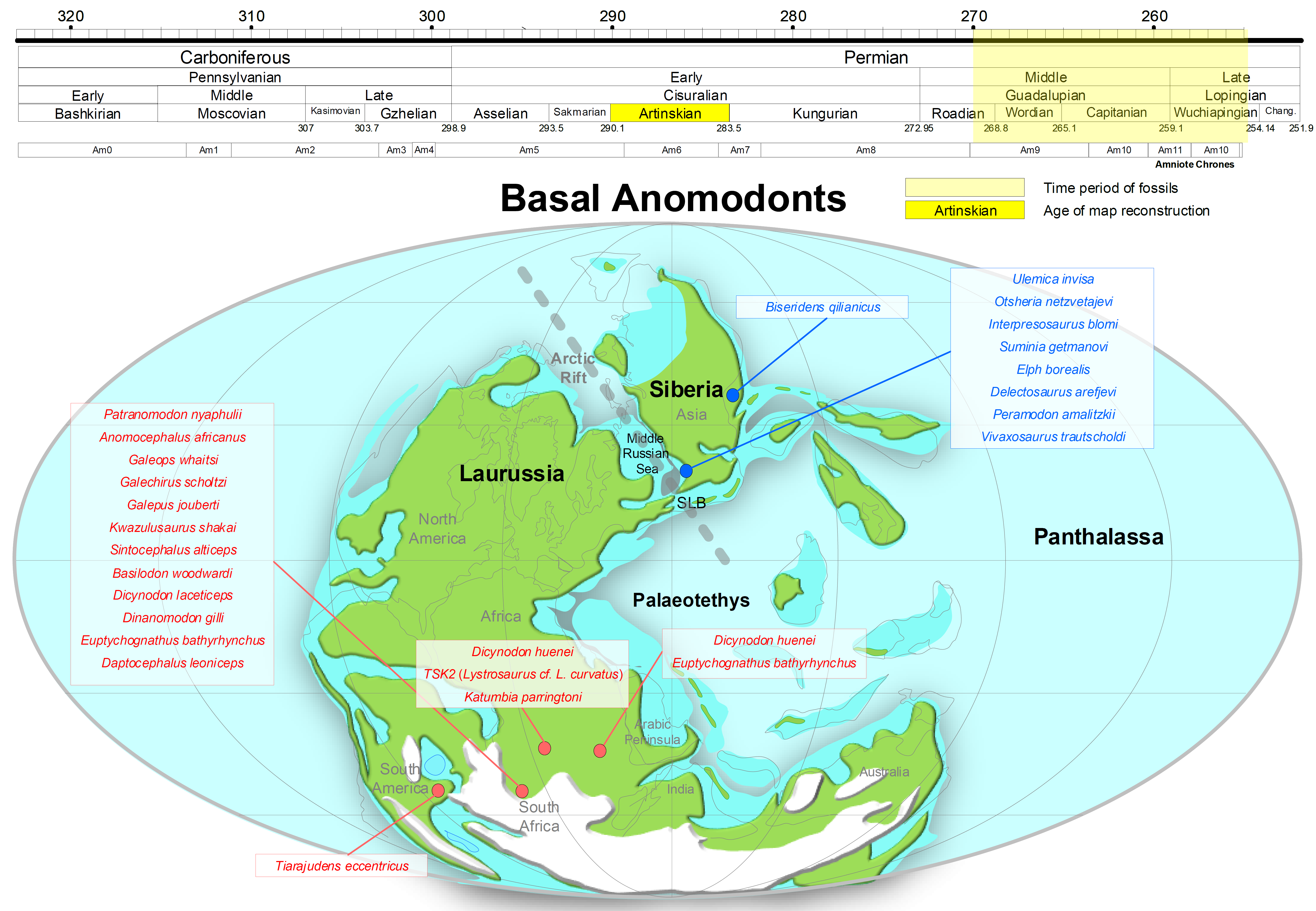


**Figure S6.**


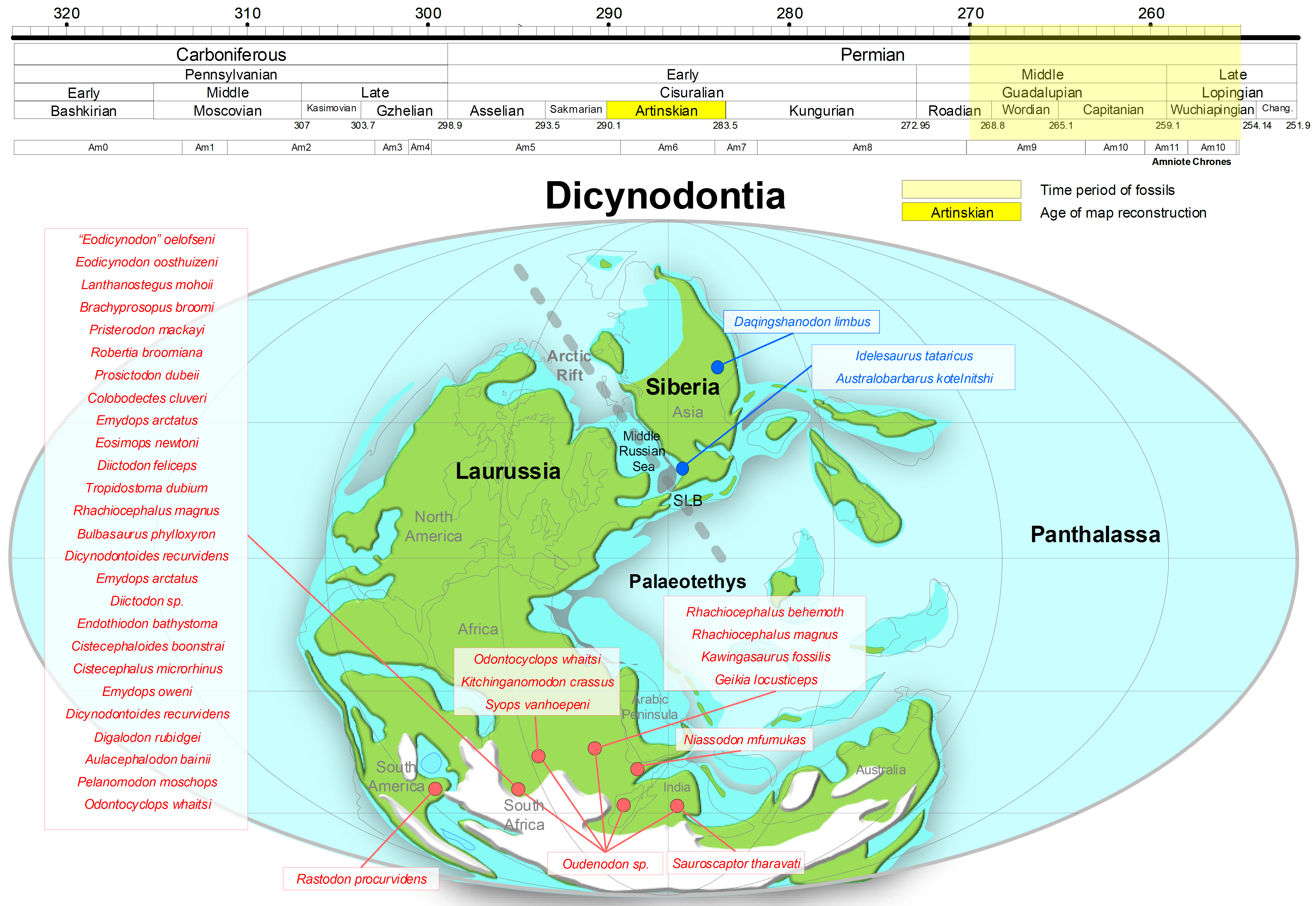


**Figure S7.**


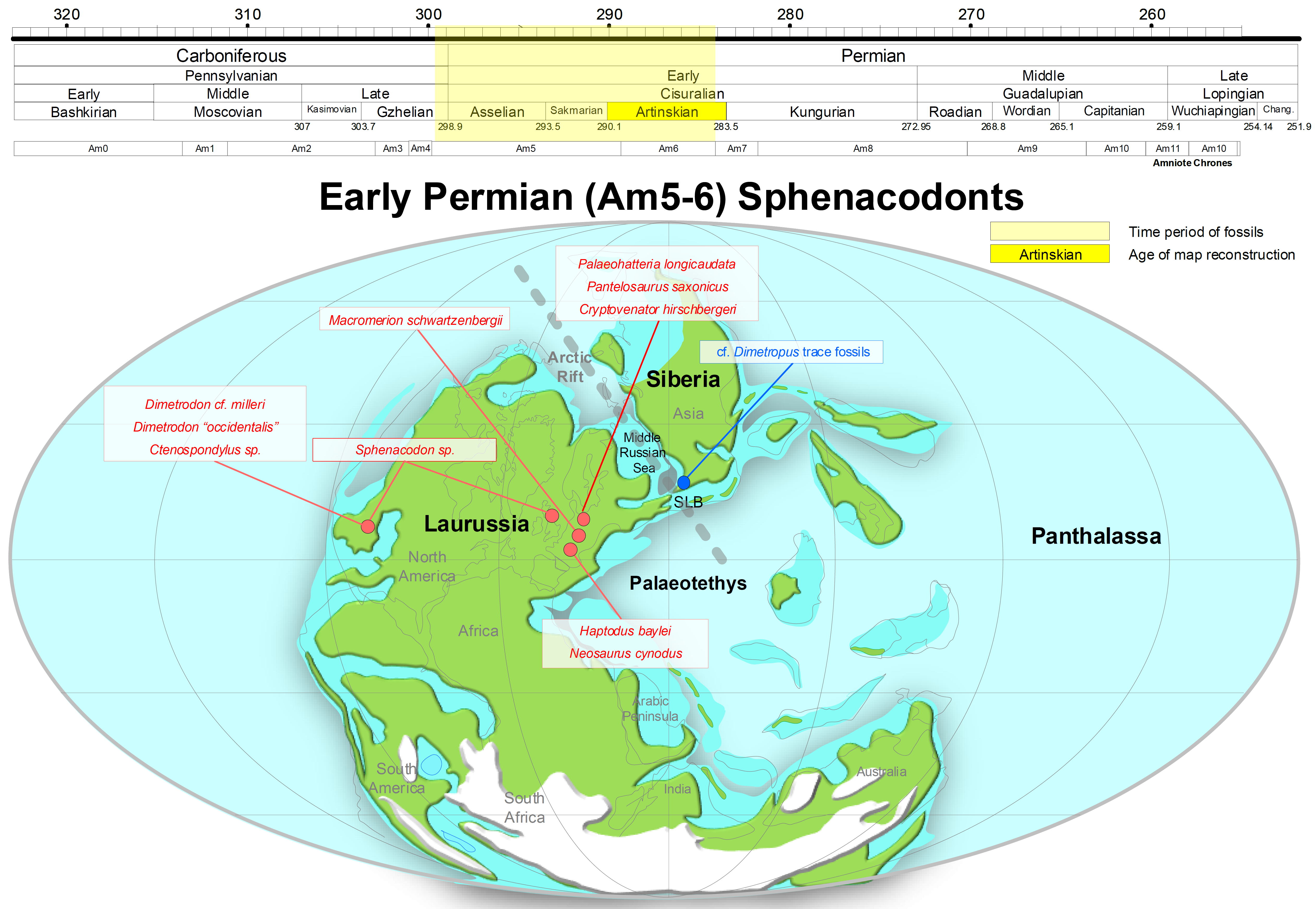


**Figure S8.**

**Supplementary figure legends**

**Figure S1.** Vicariant clado-stratigraphic pattern of the therapsid groups Gorgonopsia, Biarmosuchia, and Therocephalia. See the legends of Fig. 1 and 3 for more explanations.

**Figure S2.** Vicariant clado-stratigraphic pattern of the therapsid group Anomodontia. See the legends of Fig. 1 and 3 for more explanations.

**Figure S3:** Distribution of Gorgonopsia upon a palaeogeographical map of Pangaea. Map modified from ref. [1].

**Figure S4:** Distribution of Biarmosuchia upon a palaeogeographical map of Pangaea. Map modified from ref. [1].

**Figure S5:** Distribution of Therocephalia upon a palaeogeographical map of Pangaea. Map modified from ref. [1].

**Figure S6:** Distribution of basal anomodonts upon a palaeogeographical map of Pangaea. Map modified from ref. [1].

**Figure S7:** Distribution of the anomodont group Dicynodontia upon a palaeogeographical map of Pangaea. Map modified from ref. [1].

**Figure S8:** Distribution of Early Permian pelycosaurians upon a palaeogeographical map of Pangaea. Map modified from ref. [1].

**Figure References**

- - 1. Ziegler, A. M., Hulver, M. L. & Rowley, D. B. Permian world topography and climate in Late glacial and postglacial environmental changes: Quaternary, Carboniferous–Permian and Proterozoic (ed. Martini I. P.) 111–146 (Oxford University Press, 1997).
